# Stereoselective Epoxidation of Triterpenic Allylic Alcohols and Cytotoxicity Evaluation of Synthesized Compounds

**DOI:** 10.3390/molecules28020550

**Published:** 2023-01-05

**Authors:** Gulnaz Krainova, Yulia Beloglazova, Maksim Dmitriev, Victoria Grishko

**Affiliations:** 1 Institute of Technical Chemistry, Perm Federal Research Center, Ural Branch of the Russian Academy of Sciences, Perm 614013, Russia; 2 Department of Organic Chemistry, Perm State University, Perm 614990, Russia

**Keywords:** triterpenoids, α-substituted α,β-enones, allylic alcohols, epoxidation, stereoselectivity, in silico, in vitro, cytotoxic activity

## Abstract

The epoxidation process of semi-synthetic triterpenoids 2-methyl-3-oxo-19β,28-epoxy- 18α-olean-1-ene, and its allylic alcohol derivatives were examined. 1,2α-epoxide, as the main product, was found to be formed from the starting enone exposed to *m*-chloroperbenzoic acid (mCPBA). In the case of hydroxy-directed mCPBA-oxidation of triterpenic allyl alcohols and their 3α-alkyl-substituted derivatives, inversion of C1 and C2 asymmetric centers with the formation of 1,2β-epoxyalcohols took place. The synthesis of 2,3α-epoxides was fulfilled from 2,3-dialkyl-substituted C(3) allyl alcohols by the action of pyridinium chlorochromate under [1,3]-oxidative rearrangement conditions. The transformations brought about enabled chiral oleanane derivatives with an oxygen-containing substituent at the C1, C2, and C3 atoms to be obtained. The study also provides information on in silico PASS prediction of pharmacological effects and in vitro evaluation of the cytotoxic activity of the synthesized compounds.

## 1. Introduction

Biologically active secondary plant metabolites, in particular pentacyclic triterpenoids with different carbon skeletons (dammarane, lupane, oleanane, ursane, etc.), have been widely used as promising candidates for developing drugs against various pathologies, especially metabolic and neurological disorders, and infectious and cardiovascular diseases [1,2,3,4,5,6]. Although a huge number of native and semi-synthetic triterpenoids with significant in vitro biological activity, an extremely low bioavailability inhibits the progress of hydrophobic triterpenoids as drug candidates. Usually, the relative bioavailability of a drug candidate is a function of the presence of both lipophilic and hydrophilic fragments within its structure, which determines the extent of interaction of the organic medicinal agent with lipid and/or aqueous phases [7]. Generally, introducing additional hydrophilic functional groups into triterpenic molecules with a non-polar lipophilic ring system is believed to render the nature of these compounds more hydrophilic and to play a key role in manifesting biological activity (e.g., in the case of polyoxygenated derivatives) [8,9,10]. Oxidative transformations of triterpenoids are most often focused on ring A, which already has a 3-hydroxyl group as a synthetic handle. At the same time, plant cytochrome P450 monooxygenases decorate basic pentacyclic triterpenoids (α-amyrin, β-amyrin, and lupeol) by the regioselective introduction of hydroxyl, ketone, aldehyde, carboxyl, or epoxy groups, at the typical C12, C13, C24, C28, and C30 positions [11]. There has also been shown a possibility of site-selective oxidation of pentacyclic triterpenoids at С1, C2, C7, C11, C15, C16, C28, C29, C30 positions by microbial biotransformation [12,13] as well as hydroxylation at C2, C6, C15, C16, C20, C21, C22, C23 positions using chemical C-H oxidation [9,14,15]. 

Although the A ring of pentacyclic triterpenoids is the main target for functionalization in many synthetic schemes, the examples of obtaining simultaneously oxidized C1, C2, and C3 atoms are limited [16]. At the same time, semi-synthetic 28/30-ester derivatives of 1α,2β,3β-trihydroxy-18β-olean-12-en-28-oic and 1α,2β,3β-trihydroxy-11-oxo-18β-Olean-12-en-30-oic acids, as well as a native compound 1β,2β,3β-trihydoxy-18β-urs-12-ene-23-oic-rhamnoside, which had effectively inhibited Gram-positive bacteria growth by regulating the metabolism and virulence gene expression, have been described recently [17,18,19]. Moreover, there have been synthesized new examples of antibacterial 1α,2α-epoxy-3β-hydroxy 18β-oleanolic acid ester derivatives that presumably regulate the metabolism, hemolysis, and β-lactamase gene expression [20]. The above findings prompted us to pay more attention to the transformation of the α-alkyl-substituted enone (**2**) previously synthesized from the triterpenoid allobetulone (**1**) in a few synthetic steps [21]. Introducing new oxygen-containing groups at the C1 and C2 positions of the enone (**2**) simultaneously can be attained through an epoxy group. Here, we have investigated the possibility of regio- and stereoselective epoxidation of the 2-methyl-3-oxo-19β,28-epoxy-18α-olean-1-ene (**2**) and its 3-alkylated derivatives (allylic alcohols). In addition, the in silico PASS prediction of pharmacological effects and in vitro evaluation of the cytotoxic activity of the synthesized compounds were conducted.

## 2. Results and Discussion

**Scheme 1:**
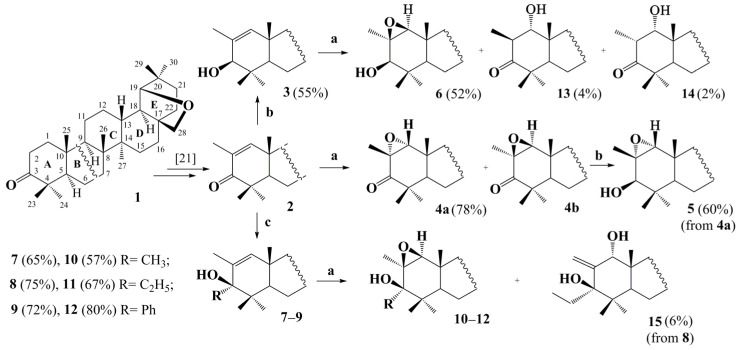
Synthesis and mCPBA-mediated epoxidation of 3β-hydroxy- (**3**) and 3α-alkyl/aryl-3β-hydroxy derivatives **7**–**9**. Reaction conditions: (**a**) mCPBA, CH_2_Cl_2_, rt; (**b**) NaBH_4_, MeOH, rt; (**c**) RMgX, (C_2_H_5_)_2_O, THF,110 °C.

There have been numerous examples of successful strategies for using polycyclic epoxides as intermediates in the synthesis of new biologically active compounds. Epoxidation of all *trans*-fused unsaturated steroids is generally accepted to proceed with the predominant formation of α-epoxide as a single product [22]. Introducing the oxirane fragment into triterpenic di-, tri-, or tetrasubstituted alkenes most often also proceeds stereoselectively and give rise to forming a least hindered isomeric α-epoxide as a single product [23,24,25,26,27,28,29]. Concurrently, some examples of epoxidizing triterpenic trisubstituted alkenes have been described with mCPBA resulting in a mixture of isomeric epoxides [25,30,31,32]. On the other hand, taking into account the structure of the starting enone **2** with *trans*-fused A/B, B/C, and C/D rings and the *syn*-stereodirecting effect of the allylic 3β-hydroxyl group in the case of triterpenic 2-methyl-1-alkenes **3** and **7**–**9**, the epoxidation process of the alkenes investigated here (Scheme 1) may involve an attack of the achiral mCPBA oxidant from both α- and β-sides.

**Figure 1:**
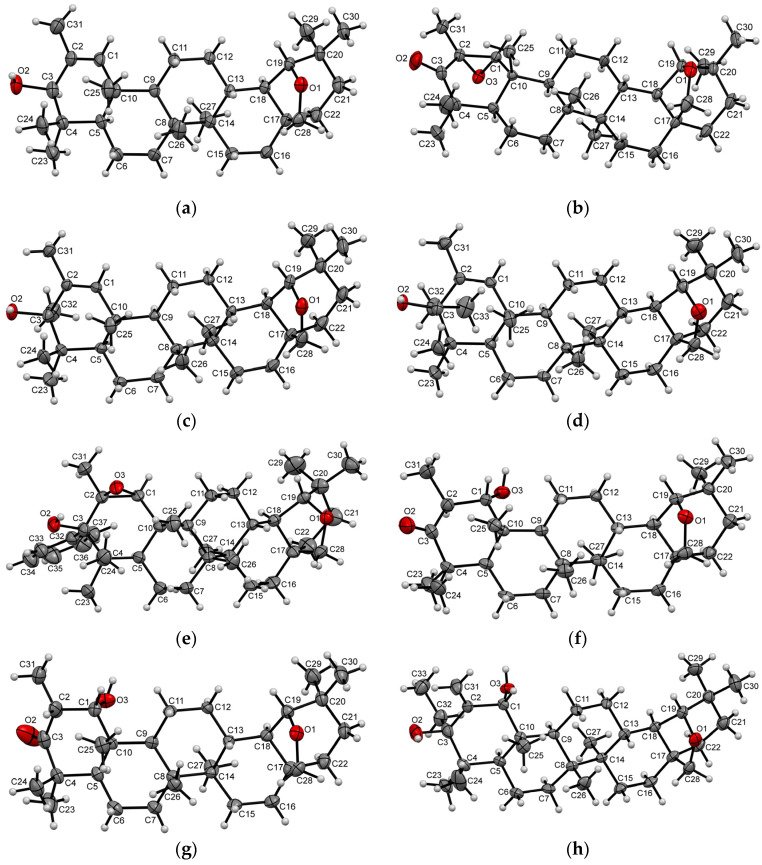
Molecular structures of compounds **3** (**a**), **4a** (**b**), **7** (**c**), **8** (**d**), **12** (**e**), **13** (**f**), **14** (**g**), **15** (**h**) with atoms represented as thermal vibration ellipsoids, with 50% probability.

The transformation procedure for allobetulone (**1**) into 2-methyl-3-oxo-19β,28-epoxy-18α-olean-1-ene (**2**) was described by us earlier [21]. To expand the range of the substrates under study, we carried out the reduction of α,β-unsaturated ketone **2** using NaBH_4_, as well as its reductive alkylation using an appropriate Grignard reagent with the formation of a secondary **3** or tertiary **7**–**9** allylic alcohols (up to 75% yield), respectively. The ^13^C NMR data confirmed the structures of the obtained allylic alcohols **3**, **7**–**9** by recording and identifying a typical signal given by a C3 atom bounded to the hydroxyl group at δ_C_ 77.56–82.59 ppm and followed by the characteristic signals of a trisubstituted double bond between C1 and C2 atoms (δ_C_ 130.35–142.98 and δ_C_ 133.72–154.93 ppm) accompanied by the signal of H1 olefinic proton at δ_H_ 5.53–5.90 ppm in the ^1^H NMR spectra. The structures of compounds **3**, **7**–**9** with the assignment of the absolute configuration of a new C3 asymmetric carbon center were finally confirmed by the X-ray crystallography technique of alcohols **3**, **7**, **8** (Figure 1a,c,d). Thus, the β-orientation of the 3-hydroxy group of compounds **3**, **7**–**9** agrees with the previously obtained data when, during the reductive alkylation of 3-oxotriterpenoids, the Grignard reagent ensured attack exclusively on the α-side to afford an α-oriented 3-alkyl/aryl substituent, due to the complexation of the Grignard reagent with the solvent THF and steric loading of axially oriented angular methyl groups at С4 and С10 atoms [33,34,35,36].

According to the ^1^H NMR spectrum, the mCPBA-mediated oxidation of triterpenic α,β-unsaturated enone **2** proceeded with the formation of a mixture of two epimeric epoxides (87:13). The signal of the H1 proton of the minor epoxide **4b** (δH 3.40 ppm) was observed in a low-field region of the ^1^H NMR spectrum of the mixture of epoxides **4a**,**b** as compared with the signal of main isomer **4a** (δH 3.38 ppm). The major product **4a** was obtained in ~80% yield after column chromatography, and its structure with a traditionally α-oriented 1,2-oxirane ring was confirmed by the single crystal X-ray diffraction analysis (Figure 1b). Thus, the β-oriented bulky angular methyl groups at the C4 and C10 atoms on the front side of triterpenoid **2** had mainly sterically prevented the attack of peracid from the β-face. Reducing epoxide **4a** by NaBH_4_ led to the corresponding 3β-hydroxy 1,2α-epoxide **5** (60% yield), the shift of the 1H proton signal in the ^1^H NMR spectrum of which was recorded at δ_H_ 3.06 ppm.

The reaction of mCPBA with secondary allylic alcohol **3** (the product of the reductive conversion of ketone **2**) led to forming epoxide **6** (52% yield) with a β-oriented oxirane ring, whose NMR signal of 1H proton appeared in a higher field region at 2.99 ppm. In this case, the possibility of the diastereofacial selectivity of the epoxidation process on the syn-side to the hydroxyl group provided a peracid approach from the sterically hindered β-side due to forming an intermolecular hydrogen bond between alcohol and mCPBA [37]. P. Kočovský showed [38] an increased steric hindrance (e.g., an axial alkyl group in a vicinal position to the hydroxy group) to impair, to some extent, the hydroxyl-directed *syn*-epoxidation of cyclic allylic alcohols with peracid, leading to forming, in some cases, a mixture of diastereoisomeric epoxy alcohols. According to NMR spectroscopy data, during mCPBA-mediated oxidation of allylic alcohols **3**, **7**–**9**, the corresponding epoxides **6**, **10**–**12** were obtained as the sole diastereoisomeric product of the reaction. Stereochemistry of the epoxidation process was confirmed using the data of a set of two-dimensional NMR spectra (^1^H–^13^C HMBC, ^1^H–^13^C HSQC, NOESY) for compound **10** and the result of X-ray diffraction analysis of epoxide **12** (Figure 1e). The correlations observed in the two-dimensional spectra confirmed the structure of the epoxide **10** and enabled the relative configuration of the C1 and C2 atoms to be defined. For example, the obvious NOE correlation between protons Н1 and Н5, Н1 and Н32, Н5 and Н32, Н6 and Н32, Н24 and Н31, Н31 and Н32, H5 and H9 in compound **10** favored the β-orientation of the oxirane ring (See Supporting Information). Comparing the ^1^H NMR spectra enabled the proton at the C1 atom of the discussed epoxides **6**, **10**–**12** to be determined as being equally α-oriented: it resonated similarly in the δH 2.72–2.99 ppm region of spectra of the epoxides **6**, **10** and **11**, and only in case of epoxide **12**, this signal had a downfield shift caused by the phenyl substituent at the С3 atom. Thus, the 3β-hydroxyl substituent turned out to be a highly effective *syn*-stereo-directing group for the semi-synthetic triterpenoids under the study, and the direction of the 2,3-epoxidation process was insensitive to the gem-dimethyl group located in a vicinal position to the hydroxyl group. The ability of the C3 geminal alkyl/aryl substituent to influence the reaction evinced the relatively bulky substituent to be more favorable for the epoxidation process, as evidenced by a higher yield in the epoxide series (CH_3_ < C_2_H_5_ < C_6_H_5_).

**Scheme 2:**
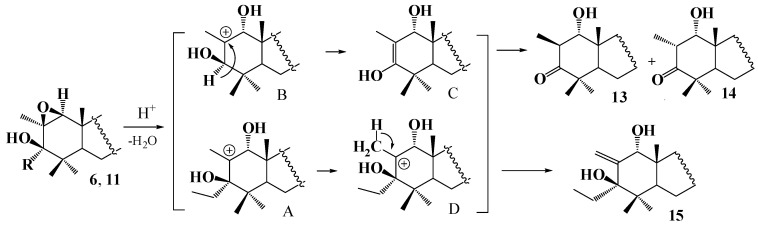
Possible reaction mechanism for isomerization of epoxides **6** and **11** to β-hydroxyketones **13**, **14** and allyl alcohol **15**.

The unexpected side products **13**–**15** (yields 2–6%) were also isolated from the reaction mixtures obtained by epoxidation of allylic alcohols **3** and **8** (Scheme 1). The structures **13**–**15** were explicitly verified by X-ray diffraction analysis, confirming the presence of a 1α-hydroxyl group combined with a 3-oxo moiety or a 2,31-*exo*-methylene group (Figure 1f,g,h). With the hints taken into account from the earlier reports on the possibility of the ring-opening of the epoxides, which proceeded through the *anti*-dihydroxylation stage [39] under the action of benzoic acid formed as a by-product during mCPBA-epoxidation, as well as the formation of β-hydroxyketones or allyl alcohols as a result of the isomerization of polyfunctional epoxides [40], the reaction pathways for transforming epoxides **6** and **11** to 1α-hydroxy derivatives **13**–**15** have been proposed (Scheme 2).

A possible mechanism for forming compounds **13**–**15** includes the stage of the acid-catalyzed ring-opening of epoxides **6**, **11** with forming 1α,2β-diol intermediate followed by the C2 dehydration to a carbocation A; the next stage involves a hydride shift for forming enol C, acid-catalyzed enolization of which yields β-hydroxyketones **13** and **14**. In the case of intermediate D, the shift of a proton from the methyl group to C2 proceeds with forming allyl alcohol **15**. Thus, isolating the compounds **13**–**15** may indirectly indicate the use of acid catalysis for opening the studied epoxides **6**, **10**–**12** as being suitable for preparing 1,3-dihydroxy but not 1,2,3-trihydroxytriterpenic derivatives.

**Scheme 3:**
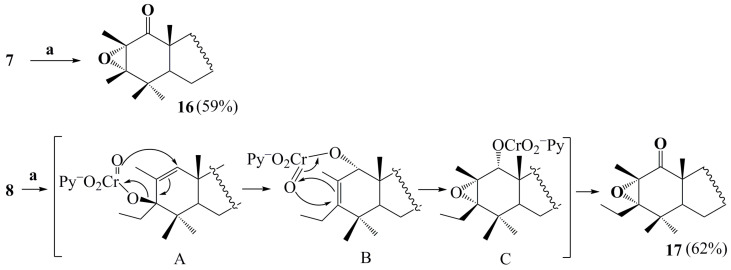
Synthesis and plausible mechanism for the formation of 2,3α-epoxides **16** and **17**. Reaction conditions: (**a**) CrO_3_·Py·HCl (PCC), CH_2_Cl_2_, rt.

**Figure 2:**
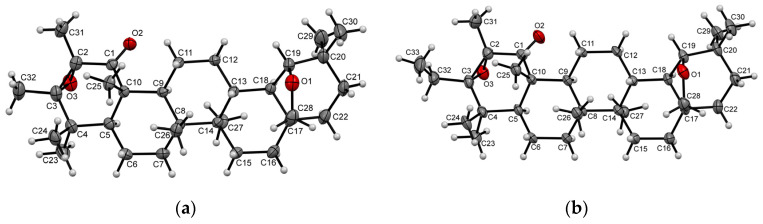
Molecular structures of compounds **16** (**a**) and **17** (**b**) with atoms represented as thermal vibration ellipsoids, with 50% probability.

An alternative synthesis of oxidized C1 derivatives from allyl alcohols was also tested. Tertiary allylic alcohols are known as tending to undergo [1,3]-oxidative rearrangement, being often used to obtain biologically active compounds or their key intermediates. Oxochromium (VI) based reagents are the most commonly used reagent systems for the [1,3]-oxidative transposition, in particular, pyridinium chlorochromate (PCC), which enables the desired products to be obtained in high yields under mild conditions [41,42]. The β-substituted α,β-unsaturated ketones are preferentially registered as the rearrangement products of the [1,3]-transposition reaction [42]. On the other hand, there have been described examples of forming β-substituted α,β-epoxy carbonyl compounds with the participation of a Collins reagent or PCC [43,44,45]. Among the studied tertiary alcohols **7**–**9**, only alcohols **7** and **8** are involved in the [1,3]-transposition reaction: the corresponding 2,3α-epoxy rearrangement products **16** and **17** were isolated as single reaction products (~60% yield) (Scheme 3). The formation of epoxides **16** and **17** having an oxo group at position C1 was evidenced by the absence of a signal of olefinic H1 proton in the ^1^H NMR spectra and also by the disappearance of signals of the trisubstituted double 1,2-bond and a signal of a C3 carbon atom bound to the hydroxyl group against the background of the presence of a carbonyl signal of C1 atom at δ_C_ 208.84–208.93 ppm in the ^13^C NMR spectra. X-ray diffraction analysis of compounds **16** and **17** (Figure 2a,b) also supported the proposed structures with α-orientated oxirane moiety at C2 and C3 atoms. The oxidation of the alkyl allylic alcohols **7** and **8** by PCC, which yields epoxides **16** and **17,** most likely occurs as a result of the formation of a chromate ester as a key intermediate A. This reaction is followed by 3,3-sigmatropic rearrangement to 1-oxychromate B, then 2,3-epoxychromate C is formed, hydrolysis and subsequent oxidation of which lead to α-keto-epoxide. A plausible mechanism for forming 1-oxo-2,3α-epoxides **16** and **17** is similar to that previously described for diterpenoid methyl dihydroisopimarate [45] and is represented in Scheme 3. In the case of compound **9**, the reaction fails to occur, as the formation of the α-oriented 1-chromate derivative is sterically hindered due to the C3 aryl fragment. Thus, the characteristic tendency of triterpenoids to form sterically less hindered α-oriented oxirane ring structures persists in the case of [1,3]-oxidative transposition as well.

Ring A plays an important role in realizing anticancer, antiviral, anti-inflammatory, antibacterial, antifungal, and antiparasitic activities by pentacyclic triterpenoids [1,2,3,4,5,6,16,32,33,34,35,36]. At the same time, the modification of ring A with the 1,2-oxirane fragment most often improves the antibacterial, antifungal, and anticancer properties of triterpenoids [17,46,47]. Taking into account the presence of a pharmacophoric oxirane fragment [48], different biological activities could be expected manifested by novel triterpenic epoxides **4**–**6**, **10**–**12**, **16,** and **17**. To predict appropriate pharmacological effects of the synthesized derivatives **2**–**17**, there was used an openly accessible in silico tool, the PASS (prediction of activity spectra for substances) software [49]. The current version of the PASS program (2019) predicts 5066 pharmacological effects, mechanisms of action, side effects and toxic effects, influence on gene expression, etc., with an average invariant accuracy of 0.9645 prediction values of probable activity (P_a_) or probable inactivity (P_i_) [50]. Among the pharmacological effects calculated by the PASS program for the tested structures **2**–**17**, the probability coefficient (P_a_) of antineoplastic properties’ manifestation was the highest (P_a_ 0.911–0.983), especially against such cancer types as colorectal (P_a_ 0.837–0.914), colon (P_a_ 0.833–0.911), and lung (P_a_ 0.747–0.852) cancer (Table 1).

In the next stage, the MTT method [51] was used to test the cytotoxic activity of the synthesized compounds **3**, **4a**, **5**, **9**–**12,** and **17** against six human tumor cell lines, including hepatocellular carcinoma HEpG2, colorectal carcinoma HCT 116, melanoma MS, rhabdomyosarcoma RD TE32, non-small cell lung carcinoma A549, and estrogen-dependent breast adenocarcinoma MCF7. Table 2 shows that the tested compounds were generally non-toxic (IC_50_ > 200 μM) against most cell lines, including those of colorectal and lung carcinomas. Concurrently, on the breast cancer cell line MCF-7, there was a selective cytotoxic effect of the synthesized compounds, with allylic alcohol **9** and epoxides **11** and **12** being especially active against MCF-7 cells with an IC_50_ value of 37.08–45.88 μM. At the same time, epoxide **10** with a methyl substituent at position C3 was cytotoxically inactive. Structure-activity relationship (SAR) analysis revealed a threefold increase in the activity against MS cells resulting from the reduction of the oxo group of the starting α,β-unsaturated ketone **2** with the ensuing forming of the hydroxy derivative **3**. The opposite trend was noted after α-epoxidation of the double bond of compounds **2**, **3**: hydroxy-epoxide **5** was inactive against MS cells, while the activity of epoxide **4a** was slightly higher than that of compound **3**. The most notable increase in cytotoxicity was achieved by adding a phenyl group at position C3 of compounds **2** and **3**, when cytotoxicity reached the values of 45.27 and 37.08 μM for compounds **9** and **12**, respectively, as compared with the parent enone **2** with IC_50_ 60.94 μM.

## 3. Conclusions

Alkyl enones are valuable intermediates for the total synthesis of natural products or biologically active compounds. Herein, we have shown that employing inexpensive reagents enables carrying out the regio- and stereoselective transformations of 2-methyl-3-oxo-19β,28-epoxy-18α-olean-1-ene with the formation of various chiral hydroxy epoxides, epoxy-ketones, β-hydroxy ketones, or 1,3-dihydroxy derivatives bearing C1, C2, and C3 asymmetric centers in the triterpenic A-ring, which, if manipulated further, can furnish compounds of interest and useful.

The in silico PASS evaluation predicted the highest probability of antineoplastic properties of the new compounds. According to an in vitro study, allylic alcohol **9** and epoxides **11** and **12** have selective cytotoxicity against the breast cancer cell line MCF-7.

## 4. Materials and Methods

The IR spectra of the compounds dissolved in CHCl_3_ were recorded on a Bruker 66/S IFS Fourier spectrometer (Bruker, Ettlingen, Germany). The ^1^H, ^13^C, DEPT NMR spectra of the compounds dissolved in CDCl_3_ were recorded on a Bruker AVANCE II spectrometer (Bruker BioSpin GmbH, Rheinstetten, Germany) at 400 and 100 MHz, respectively. Structural assignments of compound **10** were also supported by 2D HMR (COSY ^1^H–^1^H, HSQC ^1^H–^13^C, HMBC ^1^H–^13^C, and NOESY ^1^H–^1^H) spectra. Chemical shifts (δ) were expressed in parts per million (ppm) relative to TMS as an internal standard. Optical rotation was measured on a Perkine Elmer 341 polarimeter (Perkin Elmer, Waltham, MA, USA) using the sodium D line (589 nm) as a light source for CHCl_3_ solutions. Mass spectra (MS) were determined on an Agilent 6890N/5975B chromatograph (Agilent Technologies, Wilmington, NC, USA) equipped with an HP-5ms UI capillary column (4 m × 0.25 mm, 0.25 µm; 70 eV electron impact). GC-MS analysis was performed for solutions of compounds in CH_2_Cl_2_ at 1–2 mg/mL concentration under the following conditions: an initial temperature of 100 °С, ramped to 300 °С at 40 °С/min, evaporator temperature of 310 °С, and the retention time of analyzed compounds was 3–7 min. Melting points were measured using an OptiMelt MPA100 (Stanford Research Systems, Sunnyvale, CA, USA) instrument at a heating rate of 1 °C/min. Column chromatography was carried out using 60 Å, 200–400 mesh particle size silica gel purchased from Macherey-Nagel (Duren, Germany) and the solvent mixtures of light petroleum (b.p. 40–60 °C) and ethyl acetate as an eluent. The reactions were monitored by TLC using Sorbfil plates (Sorbpolymer, Krasnodar, Russia). The solvents were purified and dried according to standard procedures [52].

The unit cell parameters and the X-ray diffraction intensities were measured on an Xcalibur Ruby diffractometer (Agilent Technologies, Cheadle, UK). Empirical absorption correction was introduced by a multi-scan method using the SCALE3 ABSPACK algorithm [53]. Using OLEX2 [54], the structures were solved with the SHELXS [55], SUPERFLIP [56], or SHELXT [57] programs and refined by the full-matrix least-squares minimization in anisotropic approximation for all non-hydrogen atoms with the SHELXL program. Hydrogen atoms bound to carbon were positioned geometrically and refined using the riding model. The OH groups’ hydrogen atoms were refined freely with isotropic displacement parameters. The contribution of the solvent electron density for **12** was removed using the SQUEEZE routine in PLATON [58].

### 4.1. General Procedure for Preparing Compounds 4, 6, 10–12, 13–15

To a solution of compound **2** (4.4 mmol), **3** (4.3 mmol), **7** (4.2 mmol), **8** (4.1 mmol), or **9** (3.7 mmol) in CH_2_Cl_2_ (20 mL) a sixfold excess of *m*-chloroperbenzoic acid was added. The reaction mixture was stirred at room temperature for 48 h. Completeness of the reaction was monitored by TLC. The reaction mixture was diluted with a 10% aqueous solution of NaOH. The reaction products were extracted with ethyl acetate (3 × 50 mL) and then washed with H_2_O to a neutral pH of the flushing waters. The organic layer was dried over anhydrous MgSO_4_, the solvent was evaporated, and the residue was purified by column chromatography on silica gel to obtain corresponding epoxides **4**, **6**, **10**–**12,** and compounds **13**–**15** as by-products. 

(1*R*,2*R*)-2-Methyl-3-oxo-(1,2),(19β,28)-diepoxy-18α*Н*-oleanane (**4a**): Yield: 78%, Rf value 0.44 (light petroleum (b.p. 40–60 °C)/ethyl acetate 5:1), colorless crystals, m.p. 250.0 °С (light petroleum (b.p. 40–60 °C)/ethyl acetate 7:1), [α]${}_{D}^{20}$+66.4 (c 0.5, CHCl_3_). IR ν (CHCl_3_) cm^−1^: 1698 (С=О). ^1^H NMR (400 MHz, CDCl_3_) δ: 0.81, 0.89, 0.93, 0.96, 0.98, 1.01, 1.08 (21H, 7s, CH_3_ × 7); 1.41 (3H, s, 3Н-31); 3.38 (1Н, s, Н-1); 3.45 and 3.77 (2Н, 2d, *J* = 8.0 Hz, 2Н-28); 3.53 (1Н, s, Н-19). ^1^H NMR (400 MHz, CDCl_3_) δ (**4b**): 3.40 (1Н, s, Н-1); 3.43 and 3.76 (2Н, 2d, *J* = 8.0 Hz, 2Н-28); 3.52 (1Н, s, Н-19). ^13^C NMR (100 MHz, CDCl_3_) δ: 13.31, 15.86, 16.07, 16.79, 19.13, 21.31, 21.90, 24.53, 26.12, 26.22, 26.43, 28.11, 28.78, 32.72, 33.02, 34.32, 36.27, 36.73, 38.38, 40.90, 41.02, 41.47, 43.81, 44.74, 46.74, 46.80, 61.28, 70.63, 71.26, 87.89, 213.34. GC-MS (*m/z*): 468.3 (M+).

(1*S*,2*R*,3*R*)-3β-Hydroxy-2-methyl-(1,2),(19β,28)-diepoxy-18α*Н*-oleanane (**6**): Yield: 52%, Rf value 0.15 (light petroleum (b.p. 40–60 °C) /ethyl acetate 5:1), colorless crystals, m.p. 213.6 °С (light petroleum (b.p. 40–60 °C)/ethyl acetate 7:1), [α]${}_{D}^{20}$+38.0 (c 0.5, CHCl_3_). IR ν (CHCl_3_) cm^−1^: 3409 (-ОН). ^1^H NMR (400 MHz, CDCl_3_) δ: 0.79, 0.82, 0.92, 0.93, 0.95, 0.99, 1.05 (21H, 7s, CH_3_ × 7); 1.40 (3H, s, 3Н-31); 2.99 (1Н, s, Н-1); 3.31 (1Н, s, Н-3); 3.45 and 3.77 (2Н, 2d, *J* = 8.0 Hz, 2Н-28); 3.54 (1Н, s, Н-19). ^13^C NMR (100 MHz, CDCl_3_) δ: 13.58, 14.70, 17.04, 17.50, 21.69, 21.94, 24.68, 24.70, 26.48, 26.59, 26.60, 28.96, 29.84, 32.90, 34.60, 34.63, 36.44, 36.93, 37.30, 37.98, 41.19, 41.65, 41.97, 46.95, 47.10, 55.30, 62.92, 71.30, 71.45, 79.73, 88.01. GC-MS (*m/z*): 470.3 (M+).

(1*S*,2*S*,3*R*)-3β-Hydroxy-2,3-dimethyl-(1,2),(19β,28)-diepoxy-18α*Н*-oleanane (**10**): Yield: 57%, Rf value 0.15 (light petroleum (b.p. 40–60 °C)/ethyl acetate 5:1), colorless crystals, m.p. 165.4 °С (light petroleum (b.p. 40–60 °C)/ethyl acetate 7:1), [α]${}_{D}^{20}$+7.4 (c 0.5, CHCl_3_). IR ν (CHCl_3_) cm^−1^: 3466 (-ОН). ^1^H NMR (400 MHz, CDCl_3_) δ: 0.81, 0.83, 0.85, 1.03, 1.08 (15H, 5s, CH_3_ × 5); 0.97 (6H, s, CH_3_ × 2); 1.02 and 1.66 (2H, 2m, 2H-15); 1.04 (1H, m, H-5); 1.08 and 1.57 (2H, 2m, 2H-12); 1.22 and 1.52 (2H, 2m, 2H-21); 1.28 and 1.41 (2H, 2m, 2H-22); 1.30 and 1.43 (2H, 2m, 2H-11); 1.37 (3H, s, 3Н-31); 1.40 and 1.44 (2H, 2m, 2H-7); 1.49 (1H, m, H-18); 1.51 (1H, m, H-13); 1.60 and 2.06 (2H, 2m, 2H-16); 1.70 (3H, s, 3Н-32); 1.85 and 1.88 (1Н, dd, *J* = 4.0, 14.0 Hz, Н-9); 2.07 and 2.09 (2H, 2m, 2H-6); 2.87 (1Н, s, Н-1); 3.47 and 3.80 (2Н, 2d, *J* = 8.0 Hz, 2Н-28); 3.56 (1Н, s, Н-19). ^13^C NMR (100 MHz, CDCl_3_) δ: 13.53 (C27), 15.95 (C26), 17.75 (C25), 20.50 (C31), 20.88 (C24), 23.28 (C32), 23.45 (C23), 24.47 (C30), 26.06 (C15), 26.27 (C16), 26.40 (C11), 26.49 (C12), 28.76 (C29), 32.22 (C6), 32.82 (C21), 34.00 (C7), 34.24 (C13), 36.26 (C20), 36.83 (C22), 37.86 (C10), 39.77 (C4), 40.84 (C14), 40.93 (C8), 41.51 (C17), 44.84 (C9), 44.96 (C5), 46.94 (C18), 63.88 (C2), 68.34 (C1), 71.26 (C28), 75.31 (C3), 88.00 (C19). GC-MS (*m/z*): 484.3 (M+).

(1*S*,2*S*,3*R*)-3-Ethyl-3β-hydroxy-2-methyl-(1,2),(19β,28)-diepoxy-18α*Н*-oleanane (**11**): Yield: 67%, Rf value 0.40 (light petroleum (b.p. 40–60 °C)/ethyl acetate 5:1), colorless crystals, m.p. 151.7 °С (light petroleum (b.p. 40–60 °C)/ethyl acetate 7:1), [α]${}_{D}^{20}$+15.8 (c 0.5, CHCl_3_). IR ν (CHCl_3_) cm^−1^: 3428 (-ОН). ^1^H NMR (400 MHz, CDCl_3_) δ: 0.73, 0.78, 0.81, 0.92, 0.93, 0.98, 1.03 (21H, 7s, CH_3_ × 7); 1.12 (3Н, t, *J* = 8.0 Hz, 3Н-33); 1.38 (3Н, s, 3Н-31); 2.72 (1Н, s, Н-1); 3.43 and 3.76 (2Н, 2d, *J* = 8.0 Hz, 2Н-28); 3.51 (1Н, s, Н-19). ^13^C NMR (100 MHz, CDCl_3_) δ: 9.44, 13.66, 16.02, 18.23, 21.35, 21.47, 23.39, 23.50, 24.49, 26.03, 26.26, 26.41, 26.48, 28.55, 28.80, 32.22, 32.77, 34.18, 36.27, 36.80, 37.97, 40.38, 40.80, 40.91, 41.52, 44.69, 44.93, 46.90, 62.85, 67.55, 71.29, 77.15, 88.01. GC-MS (*m/z*): 498.3 (M+).

(1*S*,2*S*,3*R*)-3β-Hydroxy-2-methyl-3-phenyl-(1,2),(19β,28)-diepoxy-18α*Н*-oleanane (**12**): Yield: 80%, Rf value 0.41 (light petroleum (b.p. 40–60 °C)/ethyl acetate 5:1), colorless crystals, m.p. 232.2 °С (light petroleum (b.p. 40–60 °C)/ethyl acetate 7:1), [α]${}_{D}^{20}$+18.0 (c 0.5, CHCl_3_). IR ν (CHCl_3_) cm^−1^: 3440 (-ОН). ^1^H NMR (400 MHz, CDCl_3_) δ: 0.31, 0.82, 0.95, 1.04, 1.09, 1.14 (18H, 6s, CH_3_ × 6); 1.10 (6H, 2s, CH_3_ × 2); 3.27 (1Н, s, Н-1); 3.46 and 3.78 (2Н, 2d, *J* = 8.0 Hz, 2Н-28); 3.58 (1Н, s, Н-19); 7.23 (3Н, m, Н-33, Н-37, Н-36); 7.33-7.37 (1Н, m, Н-34); 7.74 (1Н, d, *J* = 8.0 Hz, Н-35). ^13^C NMR (100 MHz, CDCl_3_) δ: 13.17, 15.32, 17.25, 17.27, 20.26, 20.84, 21.56, 24.52, 26.36 (2C), 26.43, 27.71, 28.81, 32.74, 34.25, 34.90, 36.29, 36.82, 37.27, 41.21, 41.30, 41.51, 41.93, 46.50, 46.87, 49.77, 67.22, 70.84, 71.32, 79.94, 87.84, 126.17, 126.65, 127.72, 127.89, 128.40, 142.79. GC-MS (*m/z*): 546.4 (M+).

(1*S*,2*S*)-1α-Hydroxy-2-methyl-3-oxo-19β,28-epoxy-18α*Н*-oleanane (**13**): Yield: 4%, Rf value 0.20 (light petroleum (b.p. 40–60 °C)/ethyl acetate 5:1), colorless crystals, m.p. 243.1 °С (light petroleum (b.p. 40–60 °C)/ethyl acetate 7:1), [α]${}_{D}^{20}$+3.0 (c 0.5, CHCl_3_). IR ν (CHCl_3_) cm^−1^: 1737 (С=О), 3447 (-ОН). ^1^H NMR (400 MHz, CDCl_3_) δ: 0.58, 0.79, 0.93, 0.98, 1.00, 1.04, 1.07 (21H, 7s, CH_3_ × 7); 1.19 (3H, d, *J* = 8.0 Hz, 3Н-31); 2.58 (1Н, dk, *J* = 8.0, 4.0 Hz, Н-2); 3.21 (1Н, d, *J* = 4.0 Hz, Н-1); 3.44 and 3.77 (2Н, 2d, *J* = 8.0 Hz, 2Н-28); 3.51 (1Н, s, Н-19). ^13^C NMR (100 MHz, CDCl_3_) δ: 13.28, 16.17, 16.78, 19.66, 21.37, 21.47, 24.49, 26.28, 26.37, 26.42, 28.32, 28.75, 29.61, 32.78, 33.48, 33.86, 34.49, 36.26, 36.81, 38.90, 41.05, 41.50, 41.51, 44.48, 45.50, 46.84, 53.73, 71.27, 79.10, 87.89, 215.40. GC-MS (*m/z*): 470.3 (M+).

(1*S*,2*R*)-1α-Hydroxy-2-methyl-3-oxo-19β,28-epoxy-18α*Н*-olean-1-еne (**14**): Yield: 2%, Rf value 0.28 (light petroleum (b.p. 40–60 °C)/ethyl acetate 5:1), colorless crystals, m.p. 192.9 °С (light petroleum (b.p. 40–60 °C)/ethyl acetate 7:1), IR ν (CHCl_3_) cm^−1^: 1732 (С=О), 3452 (-ОН). ^1^H NMR (400 MHz, CDCl_3_) δ: 0.78, 0.92, 0.93, 1.03, 1.15 (15H, 5s, CH_3_ × 5); and 1.05 (6H, 2s, CH_3_ × 2); 1.09 (3H, d, *J* = 8.0 Hz, 3Н-31); 3.16 (1Н, dk, *J* = 8.0, 4.0 Hz, Н-1); 3.77 (1Н, d, *J* = 4.0 Hz, Н-1); 3.44 and 3.77 (2Н, 2d, *J* = 8.0 Hz, 2Н-28); 3.51 (1Н, s, Н-19). ^13^C NMR (100 MHz, CDCl_3_) δ: 12.12, 13.58, 16.08, 16.35, 20.74, 21.34, 22.67, 24.53, 24.55, 26.23, 26.27, 26.49, 28.79, 29.69, 31.92, 32.75, 33.35, 34.25, 36.28, 36.77, 40.86, 41.31, 41.49, 41.65, 41.95, 46.91, 49.30, 71.28, 80.18, 88.00, 216.39. GC-MS (*m/z*): 452.3 (M-H_2_O).

(1*S*,3*S*)-1α,3β-Dihydroxy-3-ethyl-2-methylene-19β,28-epoxy-18α*Н*-oleanane (**15**): Yield: 6%, Rf value 0.32 (light petroleum (b.p. 40–60 °C)/ethyl acetate 5:1), colorless crystals, m.p. 180.7 °С (light petroleum (b.p. 40–60 °C)/ethyl acetate 7:1), [α]${}_{D}^{20}$+18.2 (c 0.5, CHCl_3_). IR ν (CHCl_3_) cm^−1^: 1687 (C=CH_2_), 3444 (-ОН). ^1^H NMR (400 MHz, CDCl_3_) δ: 0.73, 0.77, 0.78, 0.91, 0.92, 0.96, 0.98 (21H, 7s, CH_3_ × 7); 1.01 (3Н, t, *J* = 8.0 Hz, 3Н-33); 3.43 and 3.77 (2Н, 2d, *J* = 8.0 Hz, 2Н-28); 3.52 (1Н, s, Н-19); 3.92 (1Н, s, Н-1); 5.15 and 5.25 (2H, 2d, *J* = 4.0 Hz, 2Н-31). ^13^C NMR (100 MHz, CDCl_3_) δ: 9.01, 13.80, 15.67, 19.16, 19.44, 21.14, 23.39, 24.54, 26.23, 26.29, 26.41, 26.61, 28.81, 29.68, 32.22, 32.78, 34.42, 36.29, 36.79, 40.53, 40.86, 41.13, 41.52, 41.73, 43.00, 46.29, 46.90, 71.30, 80.04, 80.30, 88.00, 116.13, 148.66. GC-MS (*m/z*): 498.3 (M+).

### 4.2. General Procedure for Preparing Compounds 3 and 5

To a solution of compound **2** (4.4 mmol) or **4** (4.3 mmol) in MeOH (20 mL), the 10-fold excess of NaBH_4_ was added. The reaction mixture was stirred at room temperature for 40 min and for 5 min while boiled. Completeness of the reaction was monitored by TLC. Then MeOH was evaporated, and the resulting precipitate was diluted with 100 mL of 10% HCl. The products were extracted with ethyl acetate (3 × 50 mL) and then washed with H_2_O to a neutral pH of the flushing waters. The organic layer was dried over anhydrous MgSO_4_, the solvent was evaporated, and the residue was purified by column chromatography on silica gel to obtain corresponding compounds **3** and **5**.

(3*S*)-3β-Hydroxy-2-methyl-19β,28-epoxy-18α*Н*-olean-1-еne (**3**): Yield: 55%, Rf value 0.33 (light petroleum (b.p. 40–60 °C)/ethyl acetate 5:1), colorless crystals, m.p. 153.7 °С (light petroleum (b.p. 40–60 °C)/ethyl acetate 7:1), [α]${}_{D}^{20}$+67.0 (c 0.5, CHCl_3_). IR ν (CHCl_3_) cm^−1^: 3425 (-ОН). ^1^H NMR (400 MHz, CDCl_3_) δ: 0.79, 0.80, 0.90, 0.92, 0.96, 0.987, 0.995 (21H, 7s, CH_3_ × 7); 1.71 (3H, s, 3Н-31); 3.43 and 3.76 (2Н, 2d, *J* = 8.0 Hz, 2Н-28); 3.52 (1Н, s, Н-19); 3.73 (1H, s, H-3); 5.67 (1Н, s, H-1). ^13^C NMR (100 MHz, CDCl_3_) δ: 13.45, 16.33, 17.77, 17.84, 19.10, 20.26, 21.04, 24.53, 26.32, 26.33, 28.01, 28.81, 32.76, 34.16, 34.32, 36.27, 36.80, 37.51, 38.85, 41.01, 41.41, 41.49 (2C), 46.86, 47.71, 54.13, 71.29, 79.94, 87.92, 130.35, 134.24. GC-MS (*m/z*): 454.4 (M+).

(1*R*,2*S*,3*R*)-3β-Hydroxy-2-methyl-(1,2),(19β,28)-diepoxy-18α*Н*-oleanane (**5**): Yield: 60%, Rf value 0.28 (light petroleum (b.p. 40–60 °C) /ethyl acetate 5:1), colorless crystals, m.p. 192.9 °С (light petroleum (b.p. 40–60 °C)/ethyl acetate 7:1), [α]${}_{D}^{20}$+13.0 (c 0.5, CHCl_3_). IR ν (CHCl_3_) cm^−1^: 3437 (-ОН). ^1^H NMR (400 MHz, CDCl_3_) δ: 0.74, 0.79, 0.85, 0.927, 0.934, 0.985, 0.992 (21H, 7s, CH_3_ × 7); 1.44 (3H, s, 3Н-31); 3.06 (1Н, s, Н-1); 3.22 (1Н, s, Н-3); 3.44 and 3.76 (2Н, 2d, *J* = 8.0 Hz, 2Н-28); 3.52 (1Н, s, Н-19). ^13^C NMR (100 MHz, CDCl_3_) δ: 13.54, 15.96, 17.63, 21.26, 23.05, 23.39, 23.94, 24.53, 25.96, 26.04, 26.23, 26.41, 28.80, 32.22, 32.76, 34.14, 36.28, 36.75, 36.77, 37.87, 40.92, 40.96, 41.52, 41.61, 44.58, 46.86, 61.38, 69.96, 71.28, 77.07, 88.01. GC-MS (*m/z*): 470.4 (M+).

### 4.3. General Procedure for Preparing Compounds 7–9

Compound **2** (6.6 mmol) in small portions was added to a freshly prepared solution of CH_3_MgI (13.2 mmol), С_2_Н_5_MgBr (13.2 mmol), or С_6_Н_5_MgI (13.2 mmol) in anhydrous Et_2_O (20 mL), and then an anhydrous mixture of Et_2_O and THF in a ratio of 2:1 (15 mL) was dropwise added additionally. The reaction mixture was heated and stirred, and 1 h later, the solution was cooled to 20 °C, diluted dropwise with ice water (25 mL), then with a mixture of HCl: H_2_O (1:1, 20 mL) and stirred until the precipitate was completely dissolved (approximately 1 h). The reaction products were extracted with ethyl acetate (3 × 20 mL). The organic layer was separated and washed sequentially with a saturated solution of NaHSO_3_ and NaHCO_3_, then with a small amount of H_2_O, and dried over anhydrous MgSO_4_. The solvent was evaporated. The residue was purified by column chromatography on silica gel to obtain corresponding compounds **7**–**9**.

(3*S*)-3β-Hydroxy-2,3-dimethyl-19β,28-epoxy-18α*Н*-olean-1-еne (**7**): Yield: 65%, Rf value 0.31 (light petroleum (b.p. 40–60 °C)/ethyl acetate 5:1), colorless crystals, m.p. 106.1 °С (light petroleum (b.p. 40–60 °C)/ethyl acetate 7:1), [α]${}_{D}^{20}$+35.4 (c 0.5, CHCl_3_). IR ν (CHCl_3_) cm^−1^: 3456 (-ОН). ^1^H NMR (400 MHz, CDCl_3_) δ: 0.78, 0.84, 0.92, 0.95, 0.98 (15Н, 5s, CH_3_ × 5); 0.90 (6H, s, CH_3_ × 2); 1.22 (3H, s, 3Н-32); 1.71 (3H, s, 3Н-31); 3.43 and 3.76 (2Н, 2d, *J* = 8.0 Hz, 2Н-28); 3.52 (1Н, s, Н-19); 5.53 (1Н, s, H-1). ^13^C NMR (100 MHz, CDCl_3_) δ: 13.47, 16.51, 18.16, 18.77, 19.41, 20.70, 21.01, 23.46 (2C), 24.49, 26.21, 26.32, 26.35, 28.79, 32.75, 34.05, 34.65, 36.25, 36.80, 38.99, 39.90, 40.97, 41.27, 41.48, 46.88, 48.20, 51.43, 71.28, 77.56, 87.91, 132.36, 133.72. GC-MS (*m/z*): 468.3 (M+).

(3*S*)-3-Ethyl-3β-hydroxy-2-methyl-19β,28-epoxy-18α*Н*-olean-1-еne (**8**): Yield: 75%, Rf value 0.41 (light petroleum (b.p. 40–60 °C)/ethyl acetate 5:1), colorless crystals, m.p. 155.8 °С (light petroleum (b.p. 40–60 °C)/ethyl acetate 7:1), [α]${}_{D}^{20}$+30.4 (c 0.5, CHCl_3_). IR ν (CHCl_3_) cm^−1^: 3456 (-ОН). ^1^H NMR (400 MHz, CDCl_3_) δ: 0.79, 0.83, 0.89, 0.92, 0.93, 0.96, 0.99 (21H, 7s, CH_3_ × 7); 0.96 (3Н, t, *J* = 8.0 Hz, 3Н-33); 1.70 (3H, s, 3Н-31); 3.44 and 3.77 (2Н, 2d, *J* = 8.0 Hz, 2Н-28); 3.53 (1Н, s, Н-19); 5.67 (1Н, s, H-1). ^13^C NMR (100 MHz, CDCl_3_) δ: 11.35, 13.32, 16.49, 18.07, 19.25, 19.44, 21.14, 21.82, 23.29, 24.46, 26.26, 26.30, 26.32, 28.78, 30.08, 32.72, 34.03, 34.75, 36.22, 36.79, 38.91, 40.68, 40.96, 41.22, 41.45, 46.87, 48.27, 50.54, 71.25, 79.37, 87.86, 131.96, 134.10. GC-MS (*m/z*): 482.4 (M+).

(3*R*)-3β-Hydroxy-2-methyl-3-phenyl-19β,28-epoxy-18α*Н*-olean-1-еne (**9**): Yield: 72%, Rf value 0.46 (light petroleum (b.p. 40–60 °C)/ethyl acetate 5:1), colorless crystals, m.p. 83.5 °С (light petroleum (b.p. 40–60 °C)/ethyl acetate 7:1), [α]${}_{D}^{20}$+16.4 (c 0.5, CHCl_3_). IR ν (CHCl_3_) cm^−1^: 3432 (-ОН). ^1^H NMR (400 MHz, CDCl_3_) δ: 0.53, 0.81, 0.93, 0.94, 1.01, 1.02, 1.05 (21H, 7s, CH_3_ × 7); 1.48 (3H, s, 3Н-31); 3.44 and 3.77 (2Н, 2d, *J* = 8.0 Hz, 2Н-28); 3.56 (1Н, s, Н-19); 5.90 (1Н, s, H-1); 7.23 (1Н, m, Н-33); 7.27–7.30 (3Н, m, Н-34, Н-36, Н-37); 7.69 (1Н, dd, *J* = 1.2, 8.0 Hz, Н-35). ^13^C NMR (100 MHz, CDCl_3_) δ: 13.24, 16.53, 17.57, 19.30, 21.08, 21.82, 24.51, 26.26, 26.29, 26.31, 26.39, 28.80, 32.75, 34.10, 34.46, 36.27 (2С), 36.82, 38.84, 40.53, 41.08, 41.25, 41.49, 46.93, 47.80, 49.31, 71.29, 82.59, 87.90, 126.66, 126.97, 128.25, 131.00, 131.72, 134.30, 142.98, 154.93. GC-MS (*m/z*): 530.4 (M+).

### 4.4. General Procedure for Preparing Compounds 16 and 17

To a solution of compound **7** (4.2 mmol) or **8** (4.1 mmol) in CH_2_Cl_2_ (20 mL), a threefold excess of PCC was added. The reaction mixture was stirred at room temperature for 24 h. Completeness of the reaction was monitored by TLC. The solvent was distilled off, and the residue was diluted with water and extracted with ethyl acetate (3 × 50 mL). The organic layer was dried over anhydrous MgSO_4_, the solvent was evaporated, and the residue was purified by column chromatography on silica gel to obtain corresponding compounds **15** and **16**.

(2*S*,3*S*)-2,3-Dimethyl-(2,3),(19β,28)-diepoxy-18α*Н*-olean-1-one (**16**): Yield: 59%, Rf value 0.35 (light petroleum (b.p. 40–60 °C)/ethyl acetate, 5:1), colorless crystals, m.p. 172.5 °С (light petroleum (b.p. 40–60 °C)/ethyl acetate 7:1), [α]${}_{D}^{20}$+34.8 (c 0.5, CHCl_3_). IR ν (CHCl_3_) cm^−1^: 1716 (C=O). ^1^H NMR (400 MHz, CDCl_3_) δ: 0.79, 0.98, 1.06, 1.07, 1.10 (15H, 5s, CH_3_ × 5); 0.92 (6H, s, CH_3_ × 2); 1.33 (3H, s, 3Н-32); 1.41 (3H, s, 3Н-31); 3.43 and 3.75 (2Н, 2d, *J* = 8.0 Hz, 2Н-28); 3.54 (1Н, s, Н-19). ^13^C NMR (100 MHz, CDCl_3_) δ: 13.27, 14.06, 14.33, 15.60, 16.58, 19.46, 20.99, 24.54, 25.09, 26.12, 26.26, 26.41, 26.51, 28.79, 32.66, 32.78, 34.82, 36.28, 36.75, 37.00, 40.17, 40.41, 41.02, 41.49, 46.63, 46.66, 49.65, 63.74, 68.85, 71.35, 87.91, 208.84. GC-MS (*m/z*): 482.3 (M+).

(2*S*,3*S*)-3-Ethyl-2-methyl-(2,3),(19β,28)-diepoxy-18α*Н*-olean-1-one (**17**): Yield: 62%, Rf value 0.48 (light petroleum (b.p. 40–60 °C)/ethyl acetate 5:1), colorless crystals, m.p. 175.1 °С (light petroleum (b.p. 40–60 °C)/ethyl acetate 7:1), [α]${}_{D}^{20}$+43.2 (c 0.5, CHCl_3_). IR ν (CHCl_3_) cm^−1^: 1715 (С=О). ^1^H NMR (400 MHz, CDCl_3_) δ: 0.80, 0.92, 0.923, 0.99, 1.05, 1.148, 1.152 (21H, 7s, CH_3_ × 7); 1.08 (3Н, t, *J* = 8.0 Hz, 3Н-33); 1.44 (3H, s, 3Н-31); 3.43 and 3.76 (2Н, 2d, *J* = 8.0 Hz, 2Н-28); 3.54 (1Н, s, Н-19). ^13^C NMR (100 MHz, CDCl_3_) δ: 11.27, 13.26, 13.36, 14.77, 16.55, 19.42, 21.12, 23.58, 24.53, 25.54, 26.12, 26.25, 26.40, 26.50, 28.79, 32.61, 32.77, 34.81, 36.27, 36.75, 37.78, 40.13, 40.41, 41.02, 41.47, 46.62, 46.68, 49.58, 63.91, 71.35, 71.55, 87.89, 208.93. GC-MS (*m/z*): 496.4 (M+).

CCDC 2167086 (**3**), 2167084 (**4a**), 2167087 (**7**), 2167085 (**8**), 2167091 (**12**), 2167088 (**13**), 2169665 (**14**), 2167089 (**15**), 2167092 (**16**), and 2167090 (**17**) contain the supplementary crystallographic data for this paper. The data can be obtained free of charge from The Cambridge Crystallographic Data Centre via http://www.ccdc.cam.ac.uk/conts/retrieving.html (accessed on 3 January 2023).

### 4.5. Screening for Cytotoxic Activity of Compounds 3, 4a, 5, 7–12, 16, and 17

The cytotoxic activity of the tested compounds was determined by MTT assay [51] on HEpG2, HCT116, MS, RD TE32, A549, and MCF-7 cancer cell lines. The standardized ATCC cell lines were obtained from the N.N. Blokhin National Medical Research Center of Oncology (the Ministry of Health of the Russian Federation, Moscow, Russia). The cells were maintained in DMEM (MCF-7, HCT116, HEpG2) or RPMI 1640 (RD TE32, MS, A549) medium (PanEco, Moscow, Russia) with 10% fetal bovine serum (Biosera, Nuaille, France), 2 mM L-glutamine (PanEco, Moscow, Russia), and 1% penicillin/streptomycin (50 U/mL; 50 µg/mL) (PanEco, Russia). The cells were seeded in 96-well plates at a density of 1 × 10^4^ cells/well and incubated for 24 h in a humidified CO_2_ incubator (model 460-СЕ, Thermo Fisher Scientific, Waltham, MA, USA) at +37 °C and 5% CO_2_. The stock solutions (1 × 10^−2^ M) of the tested compounds were prepared by dissolving in DMSO and then added to the wells by a micromethod of serial twofold dilutions at the concentration range of 0.3125 to 100 µM. The cells were cultivated with the compounds for 72 h, then 20 µL of MTT solution (5 mg/mL) was added to each well, and the cells were incubated for 3 h. After incubation, the medium with the compounds was removed, and the formed formazan crystals were dissolved in 100 μL of DMSO. 1% DMSO was considered to be safe for the cells and used as a control. Doxorubicin (Tocris Bioscience, Bristol, UK) was used as a reference drug. The optical density of the DMSO solutions was measured using a microplate reader FLUOstar Optima at 544 nm (BMG Labtech, Ortenberg, Germany). The IC_50_ value was determined on the basis of dose-dependent curves with the use of the Prism 6.0 program (GraphPad Software, San Diego, CA, USA). All the experiments were reiterated thrice, and the findings were presented as a mean ± standard deviation (SD).

## Figures and Tables

**Table 1:** PASS-predicted biological activities of the compounds **2**–**17**.

Tested Compound	Estimated Activity, P_a_						
	Antineoplastic	Colorectal Cancer	Colon Cancer	Lung Cancer	Ovarian Cancer	Carcinoma	Thyroid Cancer
**2**	0.940	0.914	0.911	0.786	0.780	-	-
**3**	0.961	0.900	0.898	0.819	0.774	-	0.703
**4a, 4b**	0.983	0.902	0.900	0.819	0.758	0.742	-
**5**	0.967	0.898	0.898	0.845	0.820	0.778	0.710
**6**	0.967	0.898	0.895	0.845	0.820	0.778	0.778
**7**	0.911	0.886	0.884	0.786	0.710	-	-
**8**	0.911	0.886	0.884	0.769	-	-	-
**9**	0.911	0.870	0.868	0.747	0.744	-	-
**10**	0.959	0.899	0.896	0.827	0.748	0.736	-
**11**	0.960	0.901	0.898	0.826	0.755	0.726	-
**12**	0.954	0.837	0.833	0.756	-	-	-
**13, 14**	0.959	0.888	0.881	0.826	0.822	-	-
**15**	0.934	0.890	0.887	0.832	0.701	-	-
**16**	0.938	0.898	0.895	0.852	0.735	-	0.716
**17**	0.950	0.856	0.852	0.810	-	-	-

**Table 2:** In vitro cytotoxic activity of the compounds **2**, **3**, **4a**, **5**, **9**–**12,** and **17**.

Tested Compound	IC_50_ (Mean ± SD), μM					
	HEpG2	HCT116	MS	RD TE32	A549	MCF-7
**2** **	132.1 ± 15.61	>200	156.1 ± 24.83	>200	136.8 ± 48.1	60.94 ± 2.32
**3**	135.6 ± 13.21	128.2 ± 15.66	52.56 ± 4.58	145.3 ± 35.3	96.42 ± 12.96	115.8 ± 6.14
**4a**	129.7 ± 13.52	129.1 ± 15.16	44.02 ± 6.80	142.3 ± 24.8	39.17 ± 5.11	97.27 ± 10.93
**5**	>200	>200	>200	>200	>200	78.72 ± 13.55
**9**	>200	>200	>200	>200	>200	45.27 ± 4.18
**10**	>200	>200	>200	>200	>200	>200
**11**	>200	>200	>200	>200	>200	45.88 ± 7.43
**12**	>200	>200	>200	>200	>200	37.08 ± 5.05
**17**	>200	>200	>200	>200	>200	117.00 ± 4.24
**DOX** *	1.78 ± 0.31	1.96 ± 0.19	1.29 ± 0.16	1.27 ± 0.03	2.04 ± 0.22	0.14 ± 0.03

* DOX—doxorubicin. ** Data [21].

## Data Availability

The crystallographic data of the synthesized derivatives (CCDC: 2167086 (**3**), 2167084 (**4a**), 2167087 (**7**), 2167085 (**8**), 2167091 (**12**), 2167088 (**13**), 2169665 (**14**), 2167089 (**15**), 2167092 (**16**), 2167090 (**17**)) can be obtained free of charge from The Cambridge Crystallographic Data Centre.

## Supplementary Materials

The following supporting information can be downloaded at: https://www.mdpi.com/article/10.3390/molecules28020550/s1, NMR spectra of synthesized compounds; Table S1: crystal data and structure refinement for **3**; Table S2: crystal data and structure refinement for **4a**; Table S3: crystal data and structure refinement for **7**; Table S4: crystal data and structure refinement for **8**; Table S5: crystal data and structure refinement for **12**; Table S6: crystal data and structure refinement for **13**; Table S7: crystal data and structure refinement for **14**; Table S8: crystal data and structure refinement for **15**; Table S9: crystal data and structure refinement for **16**; Table S10: crystal data and structure refinement for **17**.

## Sample Availability

Samples of the compounds are not available from the authors.

## References

[1] Markov A.V., Zenkova M.A., Logashenko E.B. (2017). Modulation of tumour-related signaling pathways by natural pentacyclic triterpenoids and their semisynthetic derivatives. Curr. Med. Chem..

[2] Zhou M., Zhang R.H., Wang M., Xu G.B., Liao S.G. (2017). Prodrugs of triterpenoids and their derivatives. Eur. J. Med. Chem..

[3] Miao Q., Xu J., Lin A., Wu X., Wu L., Xie W. (2018). Recent advances for the synthesis of selenium-containing small molecules as potent antitumor agents. Curr. Med. Chem..

[4] Wu H.F., Morris-Natschke S.L., Xu X.D., Yang M.H., Cheng Y.Y., Yu S.S., Lee K.H. (2020). Recent advances in natural anti-HIV triterpenoids and analogs. Med. Res. Rev..

[5] Amiri S., Dastghaib S., Ahmadi M., Mehrbod P., Khadem F., Behrouj H., Aghanoori M.-R., Machaj F., Ghamsari M., Rosik J. (2020). Betulin and its derivatives as novel compounds with different pharmacological effects. Biotechnol. Adv..

[6] Şoica C., Voicu M., Ghiulai R., Dehelean C., Racoviceanu R., Trandafirescu C., Roșca O.-J., Nistor G., Mioc M., Mioc A. (2021). Natural compounds in sex hormone-dependent cancers: The role of triterpenes as therapeutic agents. Front. Endocrinol..

[7] Nadendla R.R. (2005). Principles of Organic Medicinal Chemistry.

[8] Urban M., Klinot J., Tislerova I., Biedermann D., Hajduch M., Cisarova I., Sarek J. (2006). Reactions of activated lupane oxo-compounds with diazomethane: An approach to new derivatives of cytotoxic triterpenes. Synthesis.

[9] Michaudel Q., Journot G., Regueiro-Ren A., Goswami A., Guo Z., Tully T.P., Zou L., Ramabhadran R.O., Houk K.N., Baran P.S. (2014). Improving physical properties via C-H oxidation: Chemical and enzymatic approaches. Angew. Chem. Int. Ed..

[10] Huang R.Z., Jin L., Wang C.G., Xu X.J., Du Y., Liao N., Ji M., Liao Z.X., Wang H.S. (2018). A pentacyclic triterpene derivative possessing polyhydroxyl ring A suppresses growth of HeLa cells by reactive oxygen species-dependent NF-κB pathway. Eur. J. Pharmacol..

[11] Guo H., Wang H., Huo Y.X. (2020). Engineering critical enzymes and pathways for improved triterpenoid biosynthesis in yeast. ACS Synth. Biol..

[12] Shah S., Tan H., Sultan S., Faridz M., Shah M., Nurfazilah S., Hussain M. (2014). Microbial-catalyzed biotransformation of multifunctional triterpenoids derived from phytonutrients. Int. J. Mol. Sci..

[13] Luchnikova N.A., Grishko V.V., Ivshina I.B. (2020). Biotransformation of oleanane and ursane triterpenic acids. Molecules.

[14] Berger M., Knittl-Frank C., Bauer S., Winter G., Maulide N. (2020). Application of relay C−H oxidation logic to polyhydroxylated oleanane triterpenoids. Chem.

[15] Mu T., Wei B., Zhu D., Yu B. (2020). Site-selective C-H hydroxylation of pentacyclic triterpenoids directed by transient chiral pyridine-imino groups. Nat. Commun..

[16] Borkova L., Hodon J., Urban M. (2018). Synthesis of betulinic acid derivatives with modified A-ring and their application as potential drug candidates. Asian J. Org. Chem..

[17] Huang L., Luo H., Li Q., Wang D., Zhang J., Hao X., Yang X. (2015). Pentacyclic triterpene derivatives possessing polyhydroxyl ring A inhibit Gram-positive bacteria growth by regulating metabolism and virulence genes expression. Eur. J. Med. Chem..

[18] Huang L.R., Hao X.J., Li Q.J., Wang D.P., Zhang J.X., Luo H., Yang X.S. (2016). 18β-glycyrrhetinic acid derivatives possessing a trihydroxylated a ring are potent gram-positive antibacterial agents. J. Nat. Prod..

[19] Motlhanka D., Houghton P., Miljkovic-Brake A., Habtemariam S. (2010). A novel pentacyclic triterpene glycoside from a resin of *Commiphora glandulosa* from Botswana. Afr. J. Pharm. Pharmacol..

[20] Liang Z.M., Wang X.H., Huang L.R., Li Q.J., Guan T.Q., Hao X.J., Luo H., Yang X.S. (2016). 1α,2α-Epoxy-3β-hydroxy oleanolic acid derivatives regulation of the metabolism, haemolysis and β-lactamase gene expression in vitro and their structure–microbicidal activity relationship. Bioorganic Med. Chem. Lett..

[21] Konysheva A.V., Zhukova A.E., Dmitriev M.V., Grishko V.V. (2018). Synthesis and intramolecular cyclization of a 2,3-seco-oleanane triterpenoid with an ethylketone fragment. Chem. Nat. Compd..

[22] Hanson J.R., Hitchcock P.B., Kiran I. (1999). The stereochemistry of epoxidation of steroidal 4,6-dienes. J. Chem. Res.—Part S.

[23] Kinot J., Krumpolc M., Vystrčil A. (1966). Triterpenes. IX. Reaction of isomeric 2,3-epoxides with Grignard reagent. Collect. Czechoslov. Chem. Commun..

[24] Kehrli A.R.H., Taylor D.A.H., Niven M. (1990). The synthesis of a 1α,2α,3α-triacetoxy limonoid. J. Chem. Soc. Perkin Trans..

[25] García-Granados A., López P.E., Melguizo E., Parra A., Simeó Y. (2004). Oxidation of several triterpenic diene and triene systems. Oxidative cleavage to obtain chiral intermediates for drimane and phenanthrene semi-synthesis. Tetrahedron.

[26] Amer H., Mereiter K., Stanetty C., Hofinger A., Czollner L., Beseda I., Jordis U., Kueenburg B., Claßen-Houben D., Kosma P. (2010). Synthesis and crystal structures of ring A modified glycyrrhetinic acid derivatives derived from 2,3-oxirane and 2,3-thiirane intermediates. Tetrahedron.

[27] Kazakova O.B., Khusnutdinova E.F., Korlyukov A.A. (2011). Stereospecific epoxidation of an olean-18(19)-ene-type triterpenoid. Chem. Nat. Compd..

[28] Mikhailova L.R., Budaev A.S., Spirikhin L.V., Baltina L.A. (2019). Oxidation of licorice-root triterpene-acid derivatives by m-chloroperbenzoic acid. Chem. Nat. Compd..

[29] Pakulski Z., Cmoch P., Korda A., Luboradzki R., Gwardiak K., Karczewski R. (2021). Rearrangements of the betulin core. Synthesis of terpenoids possessing the bicyclo[3.3.1]nonane fragment by rearrangement of lupane-type epoxides. J. Org. Chem..

[30] Paryzek Z. (1978). Epoxidation of lanost-9(11)-enes. The effect of a β-carbonyl group upon the stereochemistry of epoxidation. J. Chem. Soc. Perkin Trans..

[31] Kvasnica M., Tislerova I., Sarek J., Sejbal J., Cisarova I. (2005). Preparation of new oxidized 18-α-oleanane derivatives. Collect. Czech. Chem. Commun..

[32] Tolmacheva I.A., Shelepen’kina L.N., Shashkov A.S., Grishko V.V., Glushkov V.A., Tolstikov A.G. (2007). Reaction of 3-acetoxy-(2,3),(19β,28)-diepoxyoleanane with cyclic and linear amines. Chem. Nat. Compd..

[33] Csuk R., Nitsche C., Sczepek R., Schwarz S., Siewert B. (2013). Synthesis of antitumor-active betulinic acid-derived hydroxypropargylamines by copper-catalyzend mannich reactions. Arch. Pharm..

[34] Pereslavtseva A.V., Tolmacheva I.A., Slepukhin P.A., El’tsov O.S., Kucherov I.I., Eremin V.F., Grishko V.V. (2014). Synthesis of A-pentacyclic triterpene α,β-alkenenitriles. Chem. Nat. Compd..

[35] Konysheva A.V., Nebogatikov V.O., Tolmacheva I.A., Dmitriev M.V., Grishko V.V. (2017). Synthesis of cytotoxically active derivatives based on alkylated 2,3-seco-triterpenoids. Eur. J. Med. Chem..

[36] Konysheva A.V., Eroshenko D.V., Grishko V.V. (2019). Synthesis, Cyclization, and cytotoxic activity of 2,3-secolupane triterpenoids with an ethylketone fragment. Nat. Prod. Commun..

[37] Carey F.A., Sundberg R.J. (2007). Advanced Organic Chemistry Part A: Structure and Mechanisms.

[38] Kočovský P. (1994). Stereochemistry of epoxidation of allylic and homoallylic cyclohexene alcohols. J. Chem. Soc. Perkin Trans..

[39] Rosatella A.A., Afonso C.A.M. (2011). Brønsted acid-catalyzed dihydroxylation of olefins in aqueous medium. Adv. Synth. Catal..

[40] Jat J.L., Kumar G. (2019). Isomerization of Epoxides. Adv. Synth. Catal..

[41] Chandrasekaran S., Ganesh V., Knochel P. (2014). Oxidation adjacent to oxygen of alcohols by chromium reagents. Comprehensive Organic Synthesis.

[42] Luzzio F.A. (2012). 1,3-Oxidative transpositions of allylic alcohols in organic synthesis. Tetrahedron.

[43] Papillaud B., Tiffon F., Taran M., Miguel B.A.S., Delmond B. (1985). Part I. epoxydes diterpeniques: Synthese et reactivite d’epoxydes derives d’acides resiniques. Tetrahedron.

[44] Ley S.V., Madin A., Trost B.M. (1991). Oxidation adjacent to oxygen of alcohols by chromium reagents. Comprehensive Organic Synthesis.

[45] Kharitonov Y.V., Shul’ts E.E., Shakirov M.M. (2014). Synthetic transformations of higher terpenoids. XXXIII.*Preparation of 15,16-dihydroisopimaric acid and methyl dihydroisopimarate and their transformations. Chem. Nat. Compd..

[46] Singha R., Ghosh P. (2018). Phytochemical investigation of Sapium baccatum: Identification of 3α-hydroxy-1α, 2α-epoxy lupan. J. Indian Chem. Soc..

[47] Salomatina O.V., Sen’kova A.V., Moralev A.D., Savin I.A., Komarova N.I., Salakhutdinov N.F., Zenkova M.A., Markov A.V. (2022). Novel Epoxides of Soloxolone Methyl: An Effect of the Formation of Oxirane Ring and Stereoisomerism on Cytotoxic Profile, Anti-Metastatic and Anti-Inflammatory Activities In Vitro and In Vivo. Int. J. Mol. Sci..

[48] Marco-Contelles J., Molina M.T., Anjum S. (2004). Naturally occurring cyclohexane epoxides: Sources, biological activities and synthesis. Chem. Rev..

[49] Way2Drug Predictive Services PASS Online. http://www.pharmaexpert.ru/passonline/index.php.

[50] Poroikov V.V. (2020). Computer-aided drug design: From discovery of novel pharmaceutical agents to systems pharmacology. Biomed. Chem..

[51] Mosmann T. (1983). Rapid colorimetric assay for cellular growth and survival: Application to proliferation and cytotoxicity assays. J. Immunol. Methods.

[52] Keil B. (1963). Laboratory Technika Organicke Chemie.

[53] (2014). CrysAlisPro.

[54] Dolomanov O.V., Bourhis L.J., Gildea R.J., Howard J.A.K., Puschmann H. (2009). OLEX2: A complete structure solution, refinement and analysis program. J. Appl. Crystallogr..

[55] Sheldrick G.M. (2008). A short history of SHELX. Acta Crystallogr. Sect. A Found. Crystallogr..

[56] Palatinus L., Chapuis G. (2007). SUPERFLIP - A computer program for the solution of crystal structures by charge flipping in arbitrary dimensions. J. Appl. Crystallogr..

[57] Sheldrick G.M. (2015). Crystal structure refinement with SHELXL. Acta Crystallogr. Sect. C Struct. Chem..

[58] Spek A.L. (2015). PLATON SQUEEZE: A tool for the calculation of the disordered solvent contribution to the calculated structure factors. Acta Crystallogr. Sect. C Struct. Chem..

