# Supplementary material for: Stereoselective Epoxidation of Triterpenic Allylic Alcohols and Cytotoxicity Evaluation of Synthesized Compounds
Source: Molecules. 2023 Jan 5;28(2):550. doi: 10.3390/molecules28020550 (PMC9863255; doi:10.3390/molecules28020550)
Supplement: Supplementary file 1 [file molecules-28-00550-s001.zip › molecules-2110871-supplementary.pdf]

Supporting Information

Stereoselective Epoxidation of Triterpenic Allylic Alcohols and Cytotoxicity Evaluation of Synthesized Compounds

Krainova Gulnaz F. [a], Yulia A. Beloglazova [a], Maksim V. Dmitriev [b], Victoria V. Grishko [a,*]

[1]Institute of Technical Chemistry, Perm Federal Research Center, Ural Branch of the Russian Academy of Sciences, Acad. Korolev St. 3, 614013 Perm, Russia
[2]Department of Organic Chemistry, Perm State University, Bukirev St. 15, 614990 Perm, Russia;

*Corresponding author. Tel.: +7-342-2378265; fax: +7-342-2378262;
e-mail: *grishko.v@itcras.ru*

NMR and GC-MS spectra of synthesized compounds

^{1}H NMR (3*S*)-3β-hydroxy-2-methyl-19β,28-epoxy-18α*H*-olean-1-ene (3)

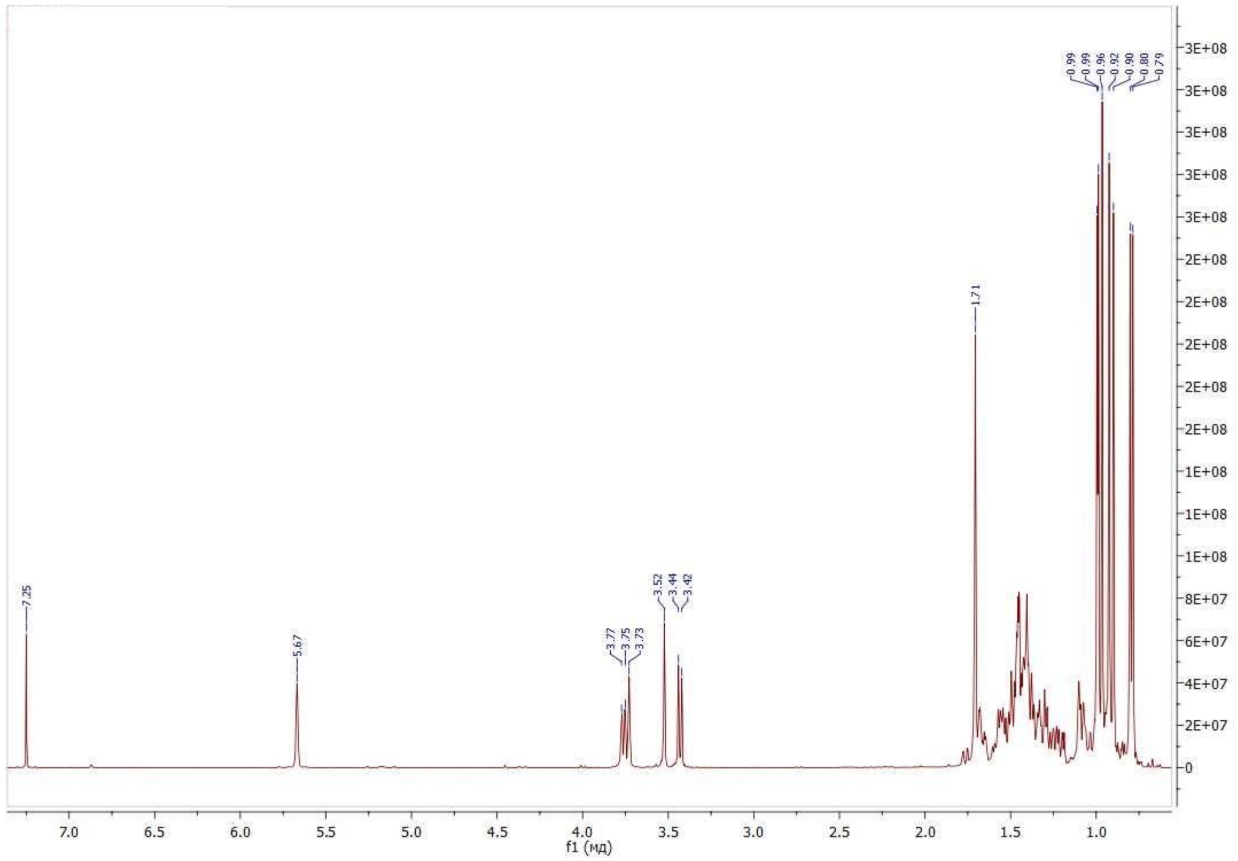

^{13}C NMR ($CDCl_3$) (3*S*)-3β-hydroxy-2-methyl-19β,28-epoxy-18α*H*-olean-1-ene (3)

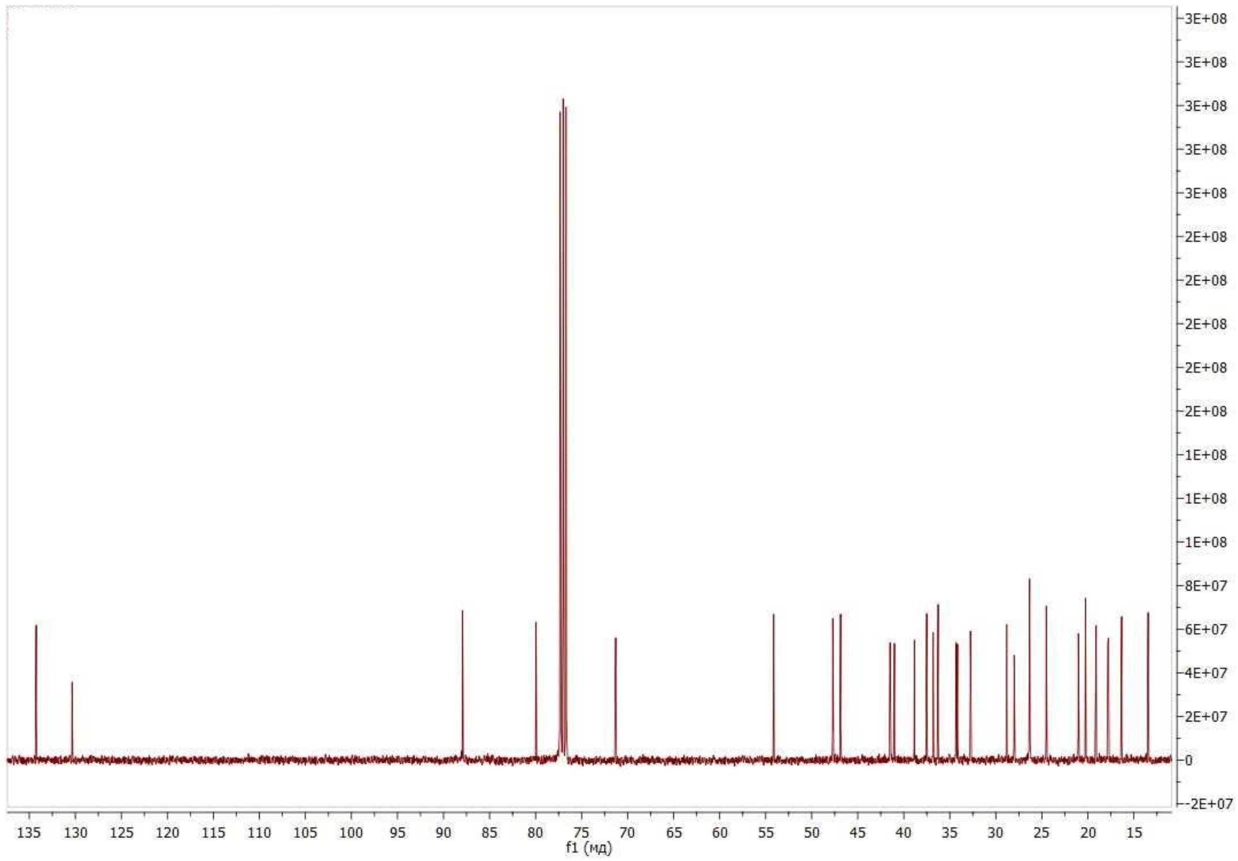

GC-MS (3*S*)-3β-hydroxy-2-methyl-19β,28-epoxy-18α*H*-olean-1-ene (3)

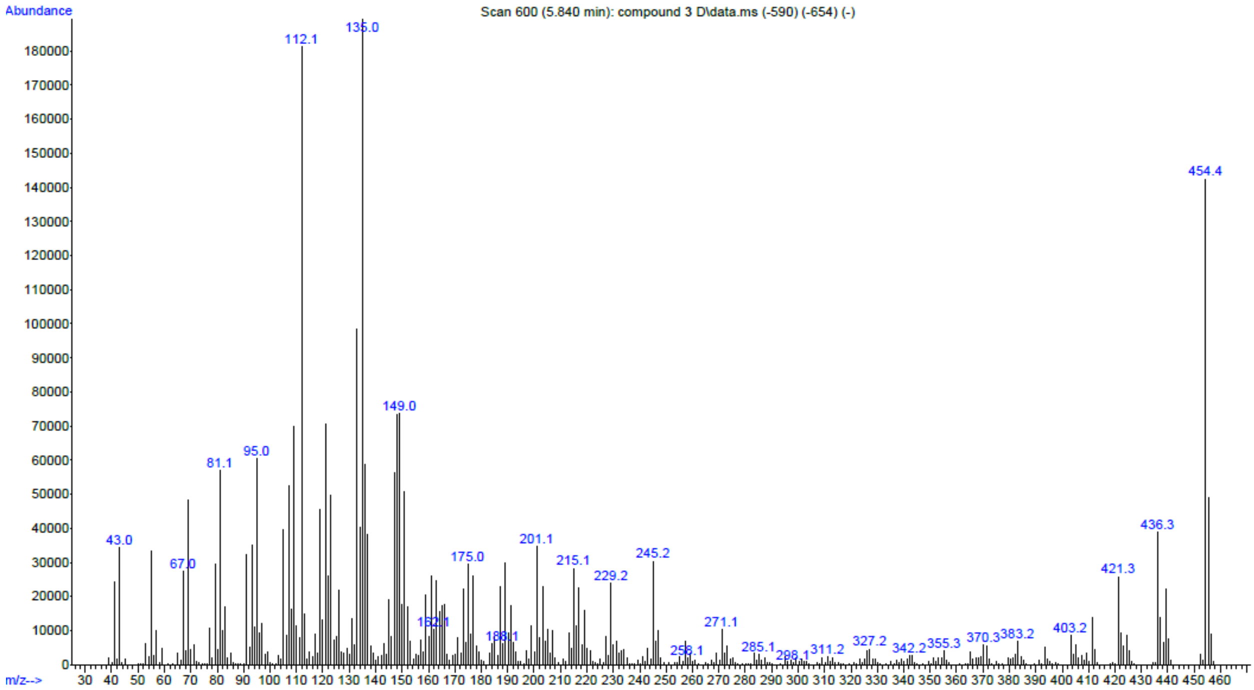

^{1}H NMR (1*R*,2*R*)-2-methyl-3-oxo-(1,2),(19β,28)-diepoxy-18α*H*-oleanane (4a)

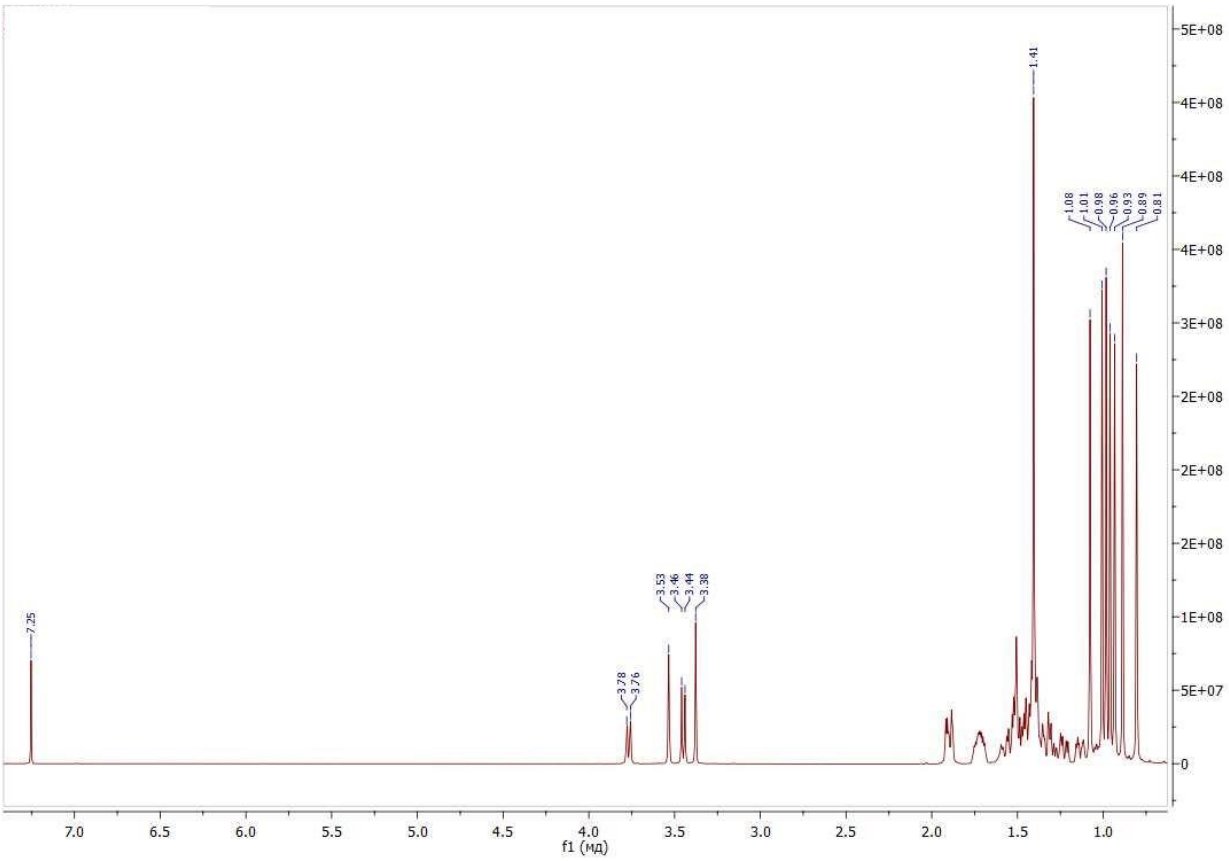

^{13}C NMR ($CDCl_3$) (1*R*,2*R*)-2-methyl-3-oxo-(1,2),(19β,28)-diepoxy-18α*H*-oleanane (4a)

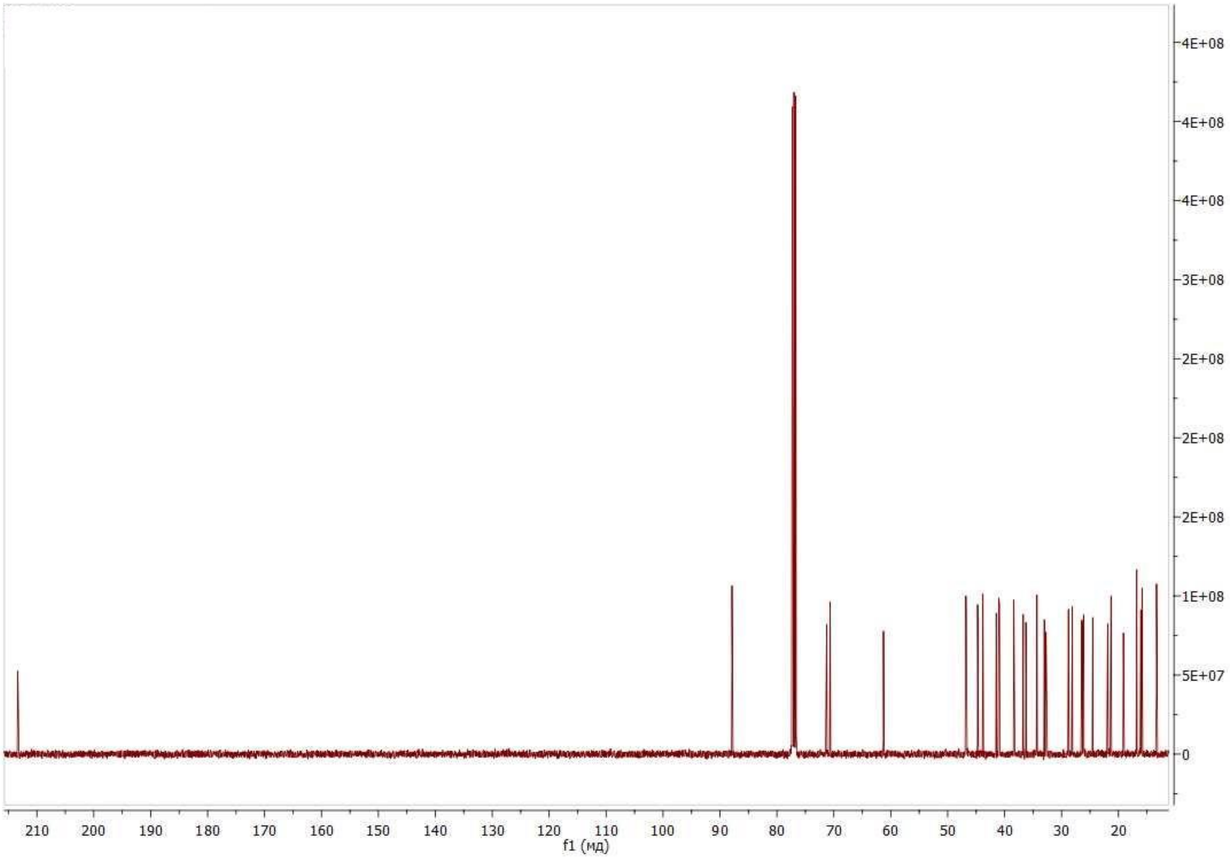

GC-MS (1*R*,2*R*)-2-methyl-3-oxo-(1,2),(19β,28)-diepoxy-18α*H*-oleanane (4a)

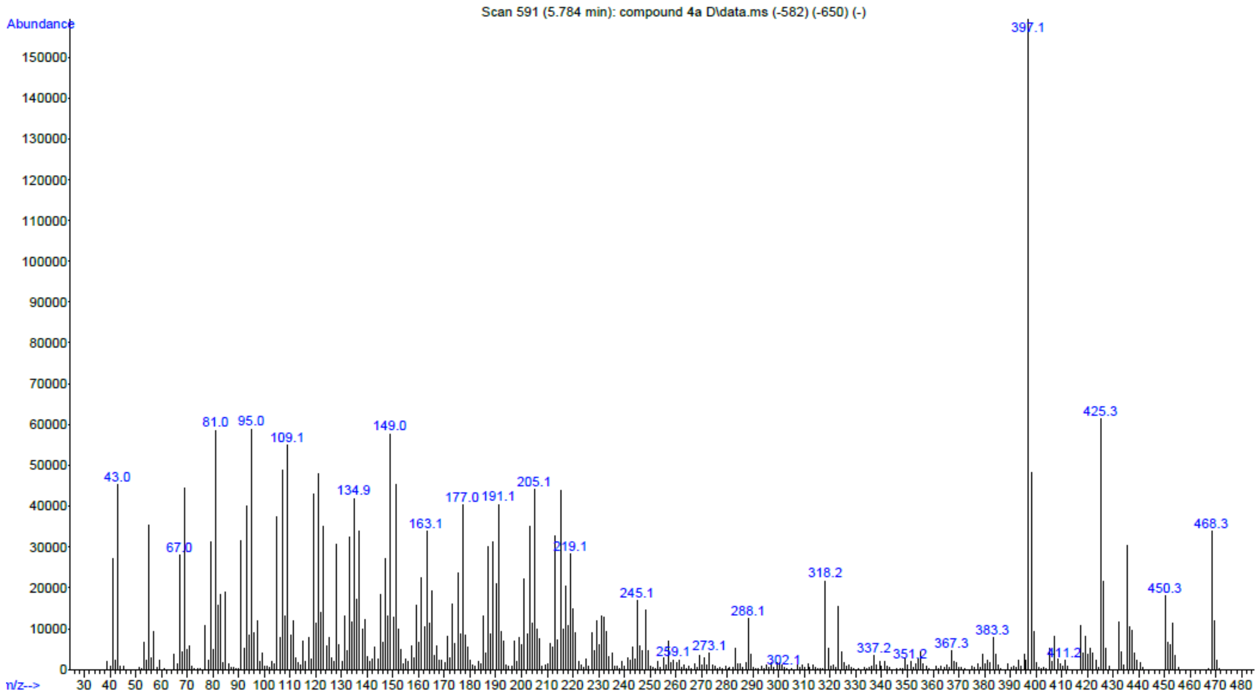

^{1}H NMR (1*R*,2*S*,3*R*)-3β-hydroxy-2-methyl-(1,2),(19β,28)-diepoxy-18α*H*-oleanane (5)

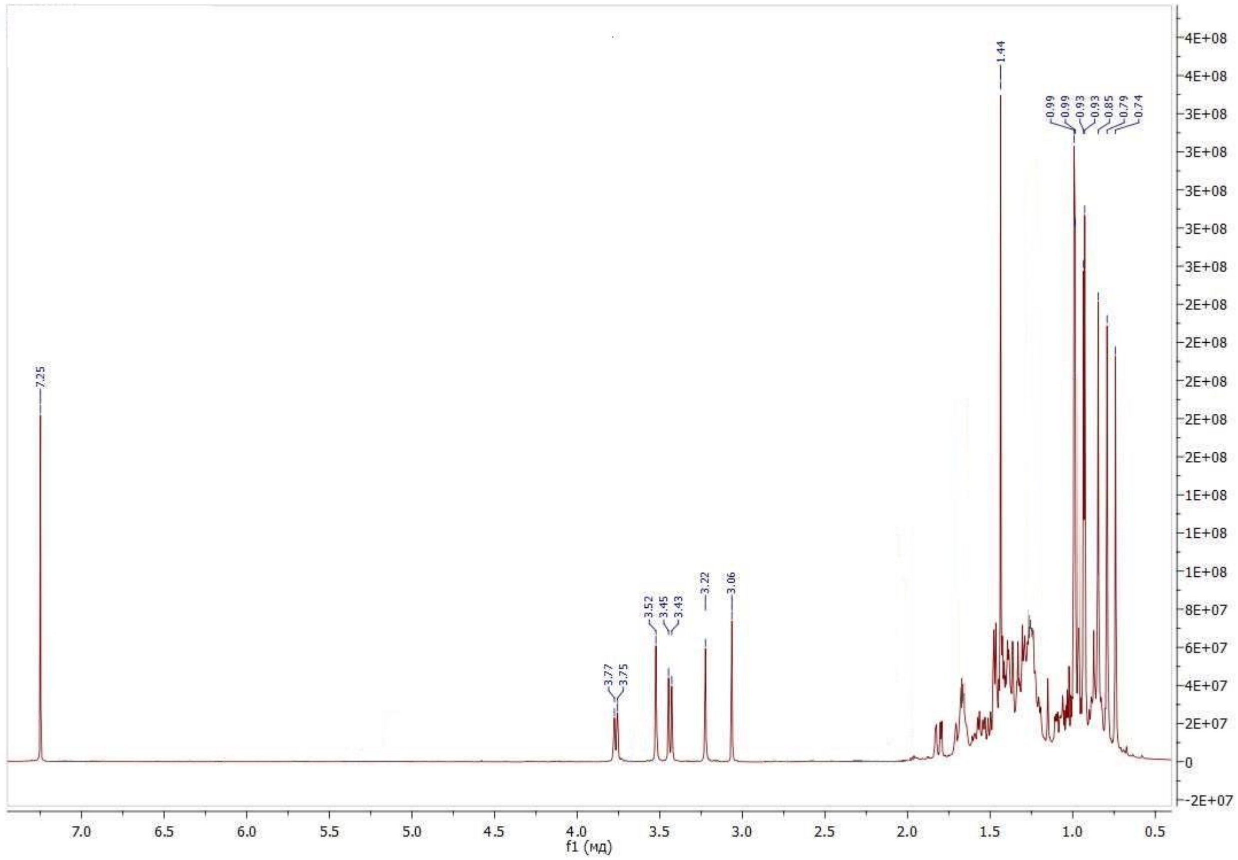

^{13}C NMR ($CDCl_3$) (1*R*,2*S*,3*R*)-3β-hydroxy-2-methyl-(1,2),(19β,28)-diepoxy-18α*H*-oleanane (5)

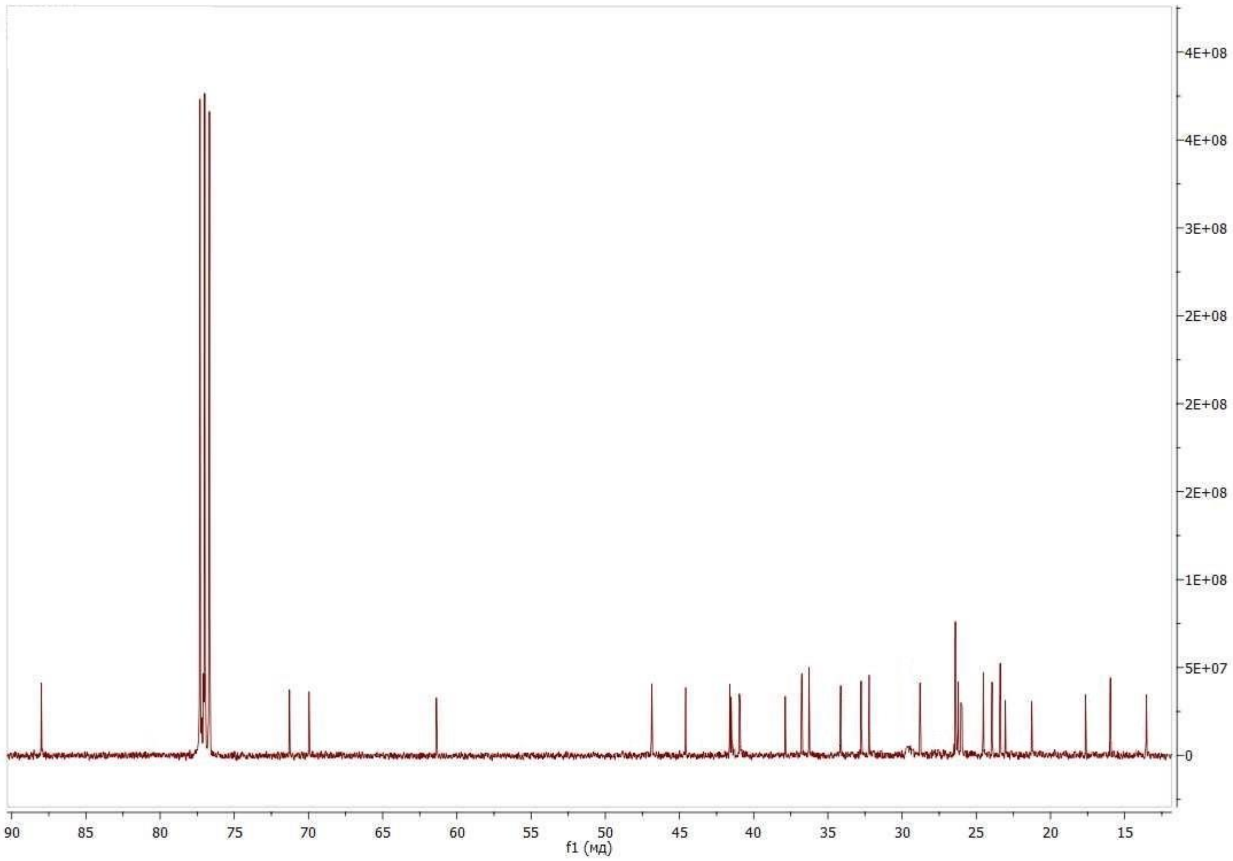

GC-MS (1*R*,2*S*,3*R*)-3β-hydroxy-2-methyl-(1,2),(19β,28)-diepoxy-18α*H*-oleanane (5)

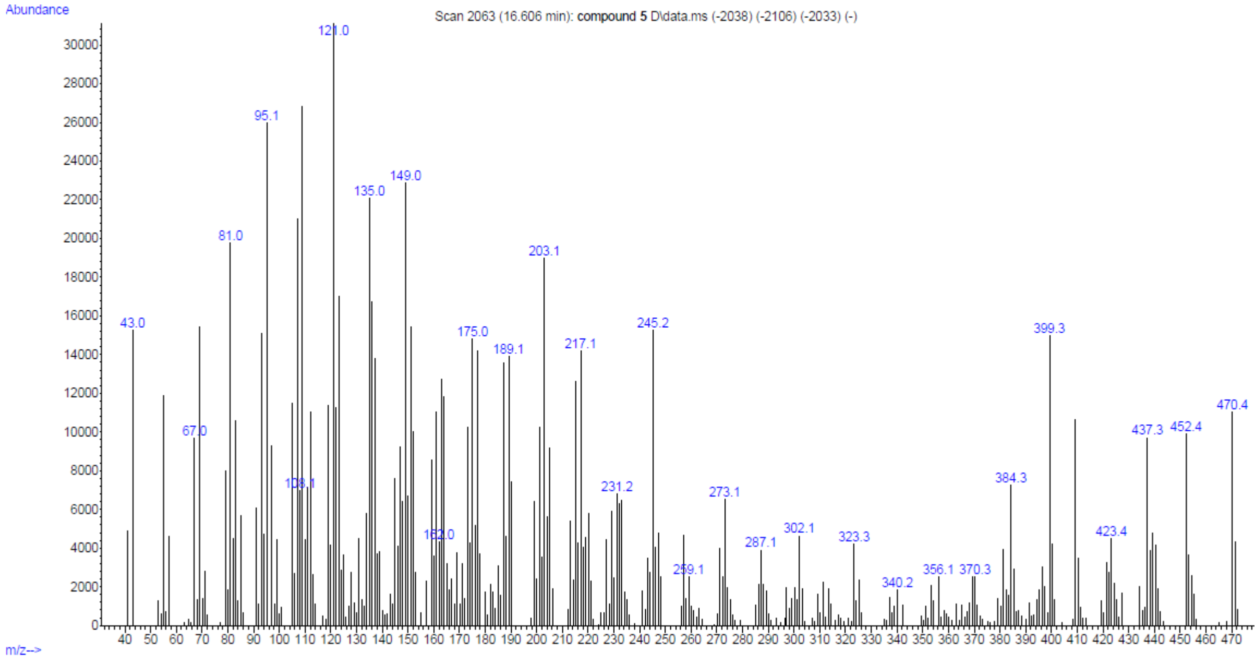

^{1}H NMR (1*S*,2*R*,3*R*)-3β-hydroxy-2-methyl-(1,2),(19β,28)-diepoxy-18α*H*-oleanane (6)

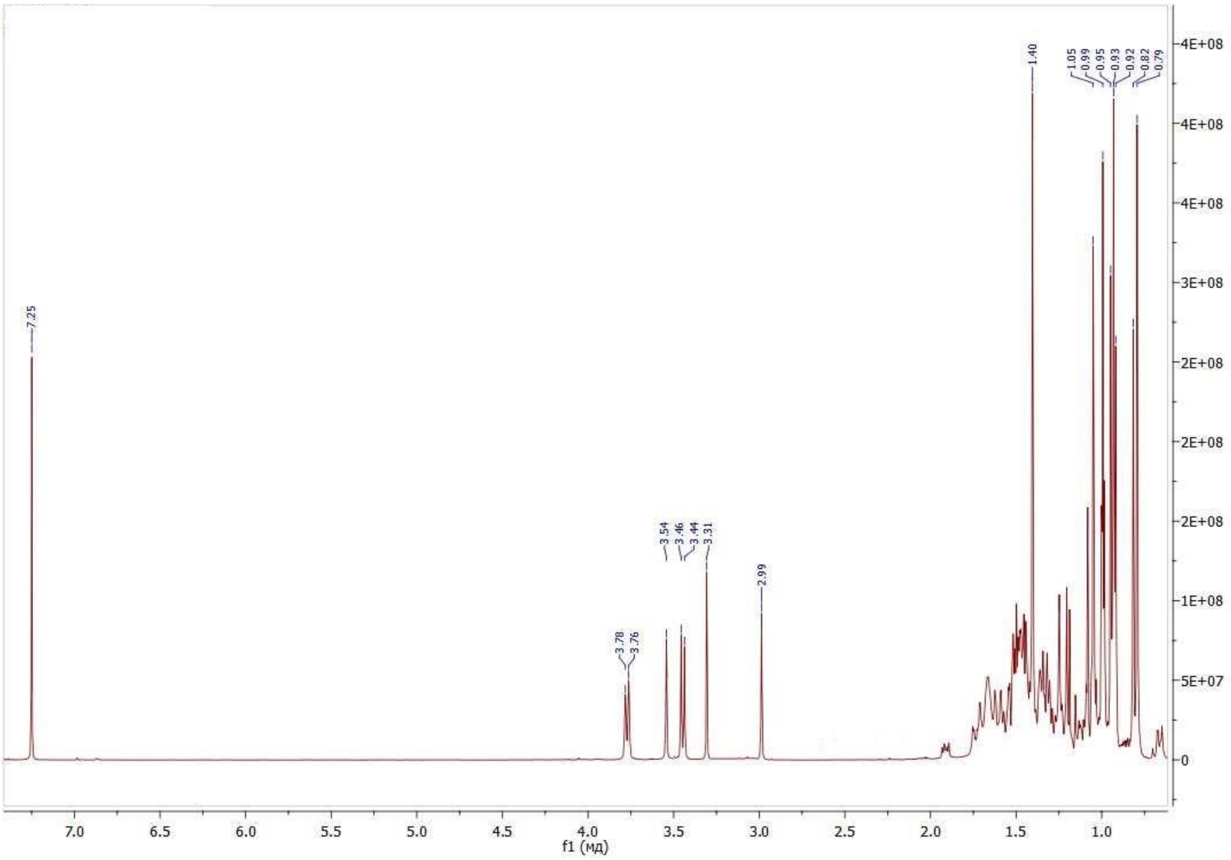

^{13}C NMR ($CDCl_3$) (1*S*,2*R*,3*R*)-3β-hydroxy-2-methyl-(1,2),(19β,28)-diepoxy-18α*H*-oleanane (6)

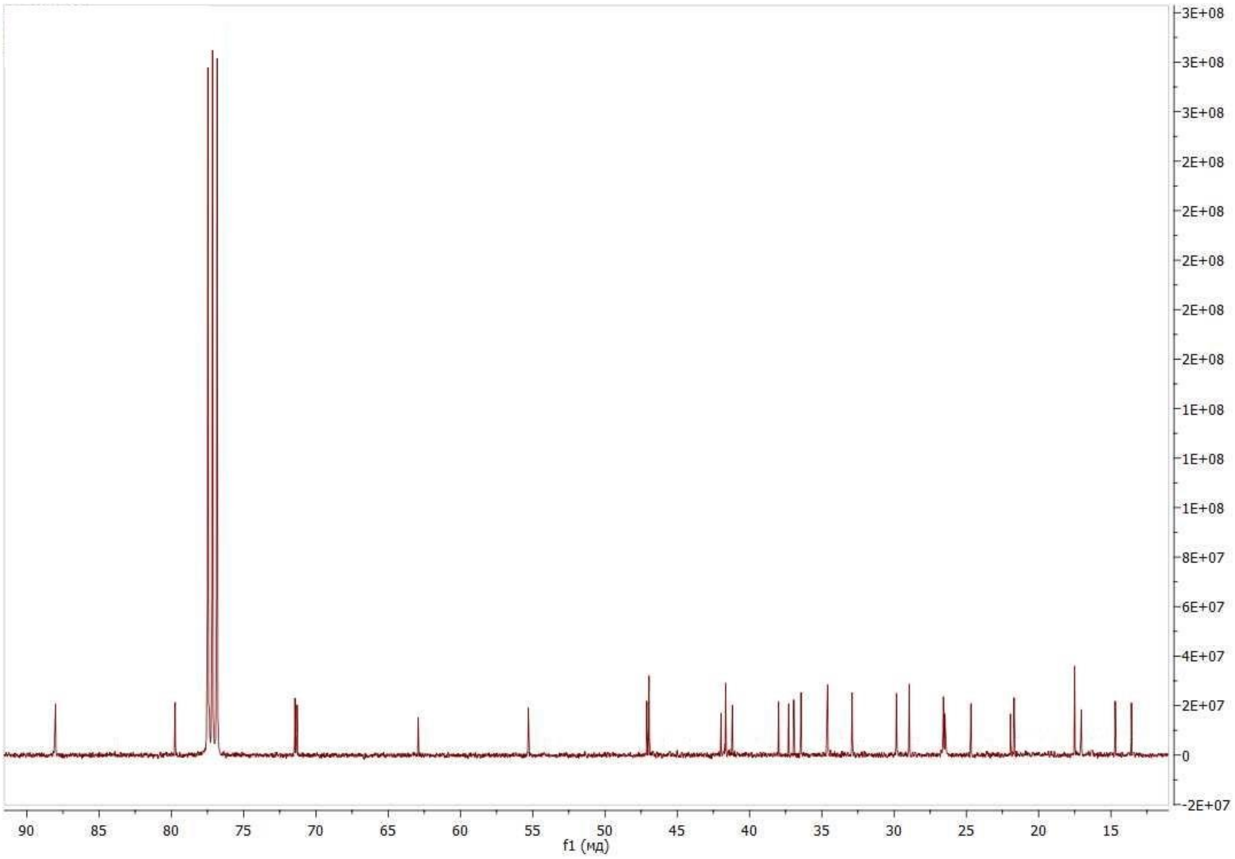

GC-MS (1*S*,2*R*,3*R*)-3β-hydroxy-2-methyl-(1,2),(19β,28)-diepoxy-18α*H*-oleanane (6)

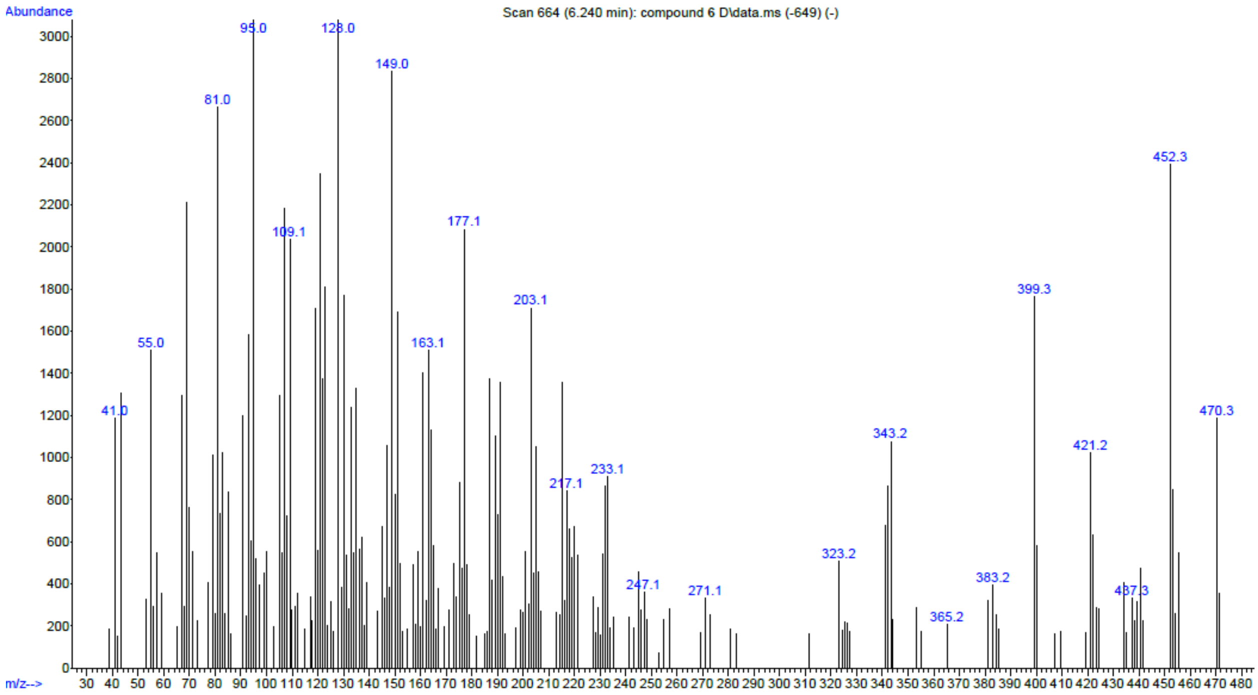

^{1}H NMR (3*S*)-3β-hydroxy-2,3-dimethyl-19β,28-epoxy-18α*H*-olean-1-ene (7)

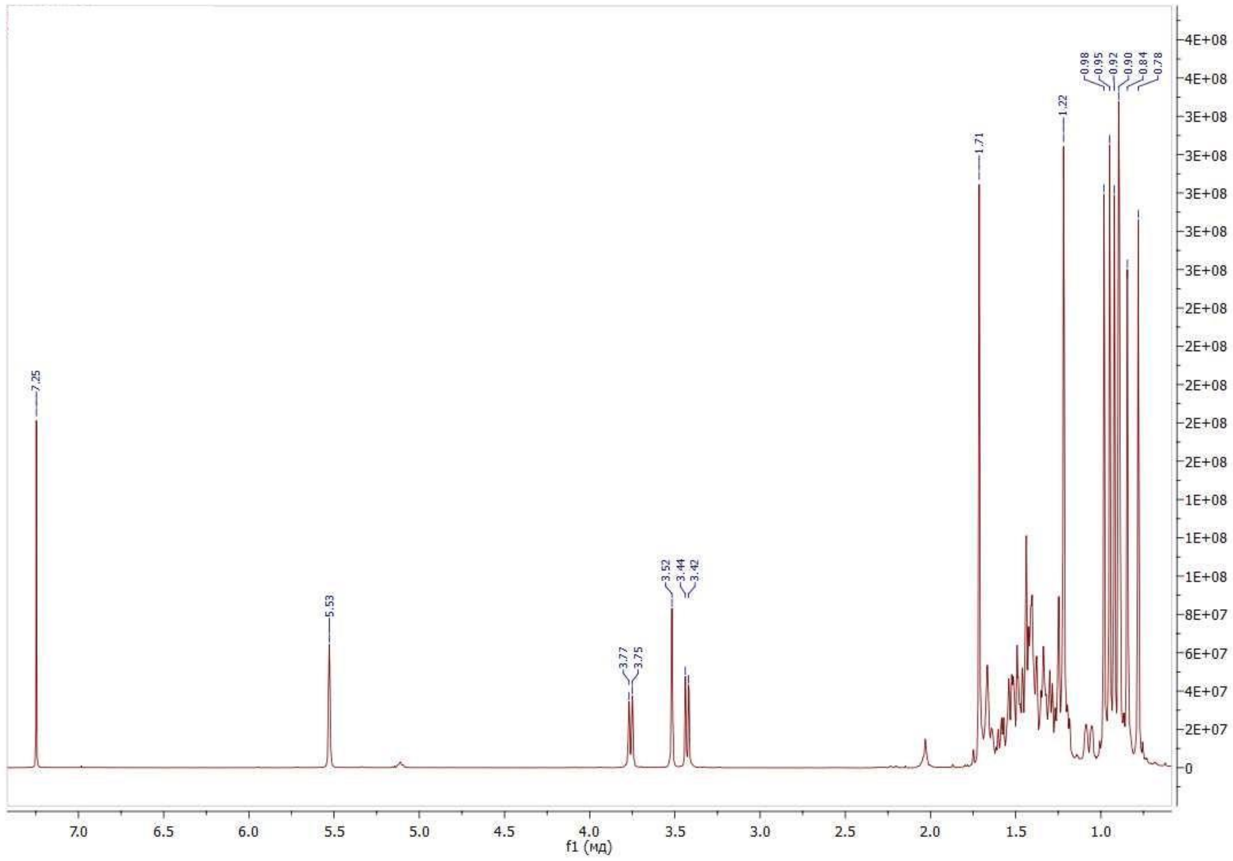

^{13}C NMR ($CDCl_3$) (3*S*)-3β-hydroxy-2,3-dimethyl-19β,28-epoxy-18α*H*-olean-1-ene (7)

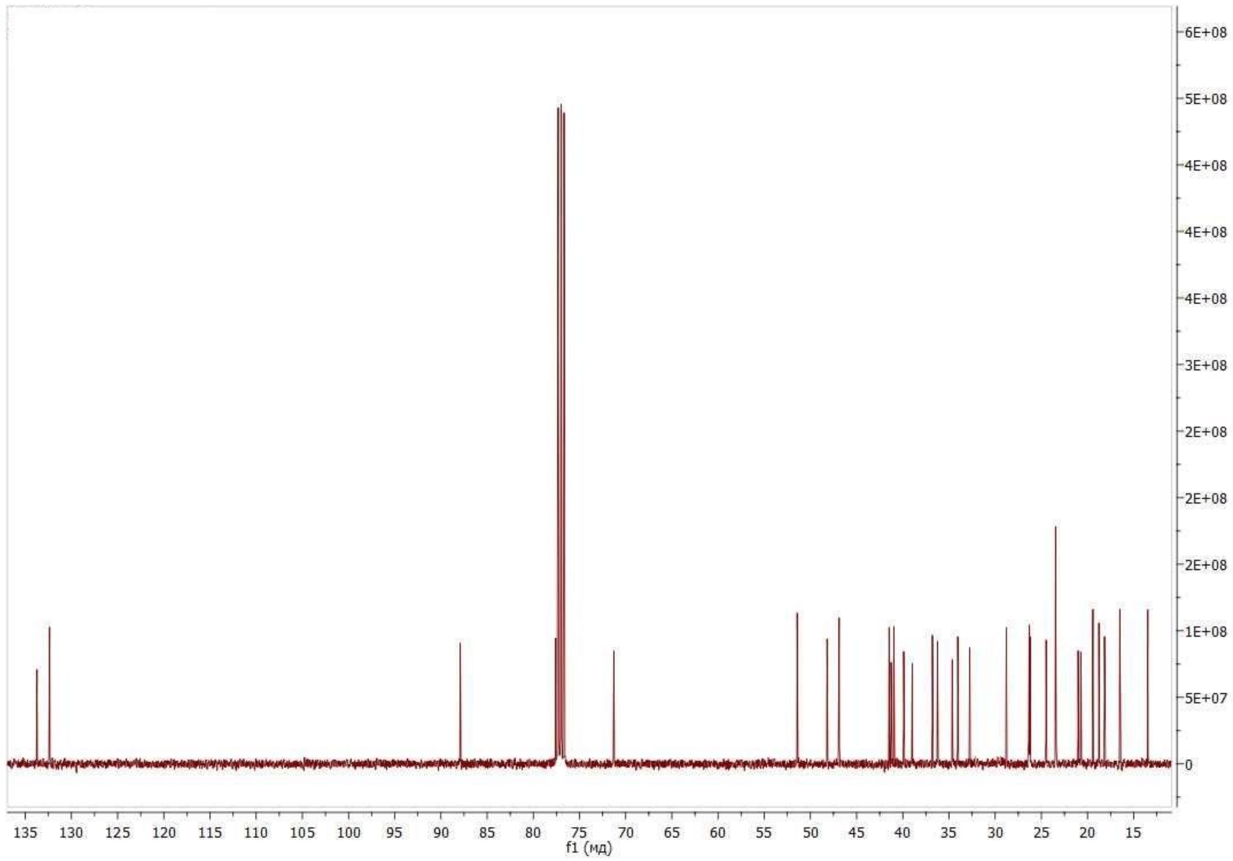

GC-MS (3*S*)-3β-hydroxy-2,3-dimethyl-19β,28-epoxy-18α*H*-olean-1-ene (7)

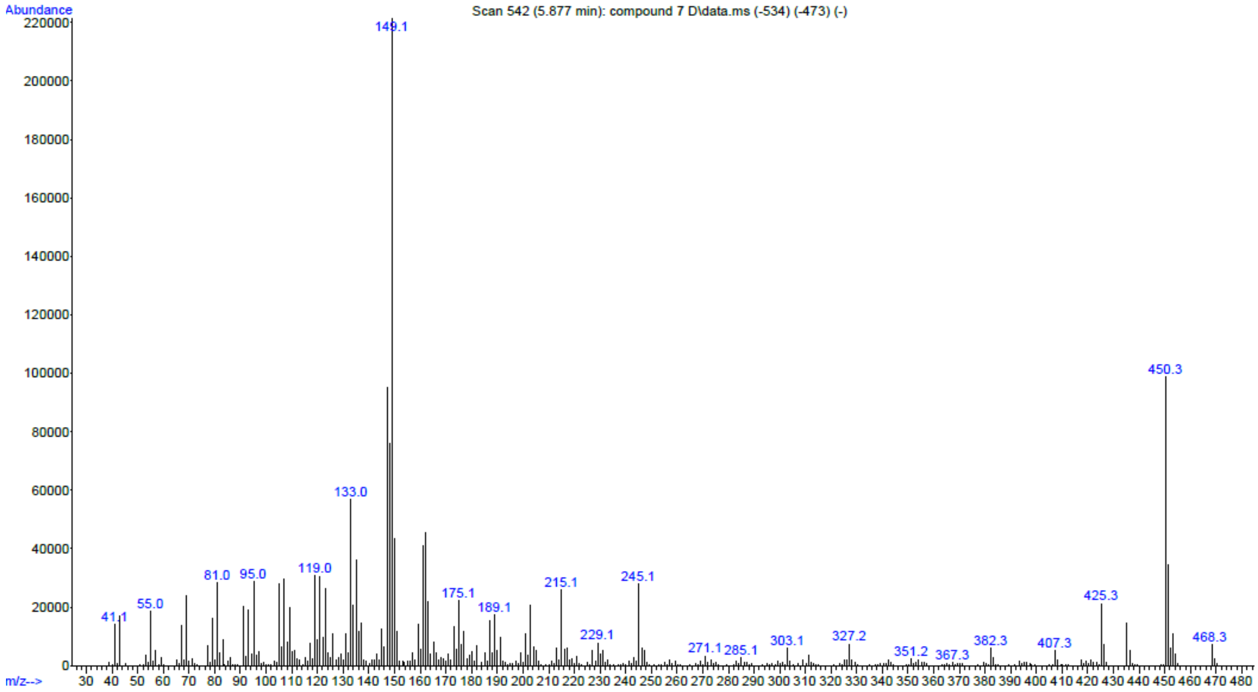

^{1}H NMR (3*S*)-3-ethyl-3β-hydroxy-2-methyl-19β,28-epoxy-18α*H*-olean-1-ene (8)

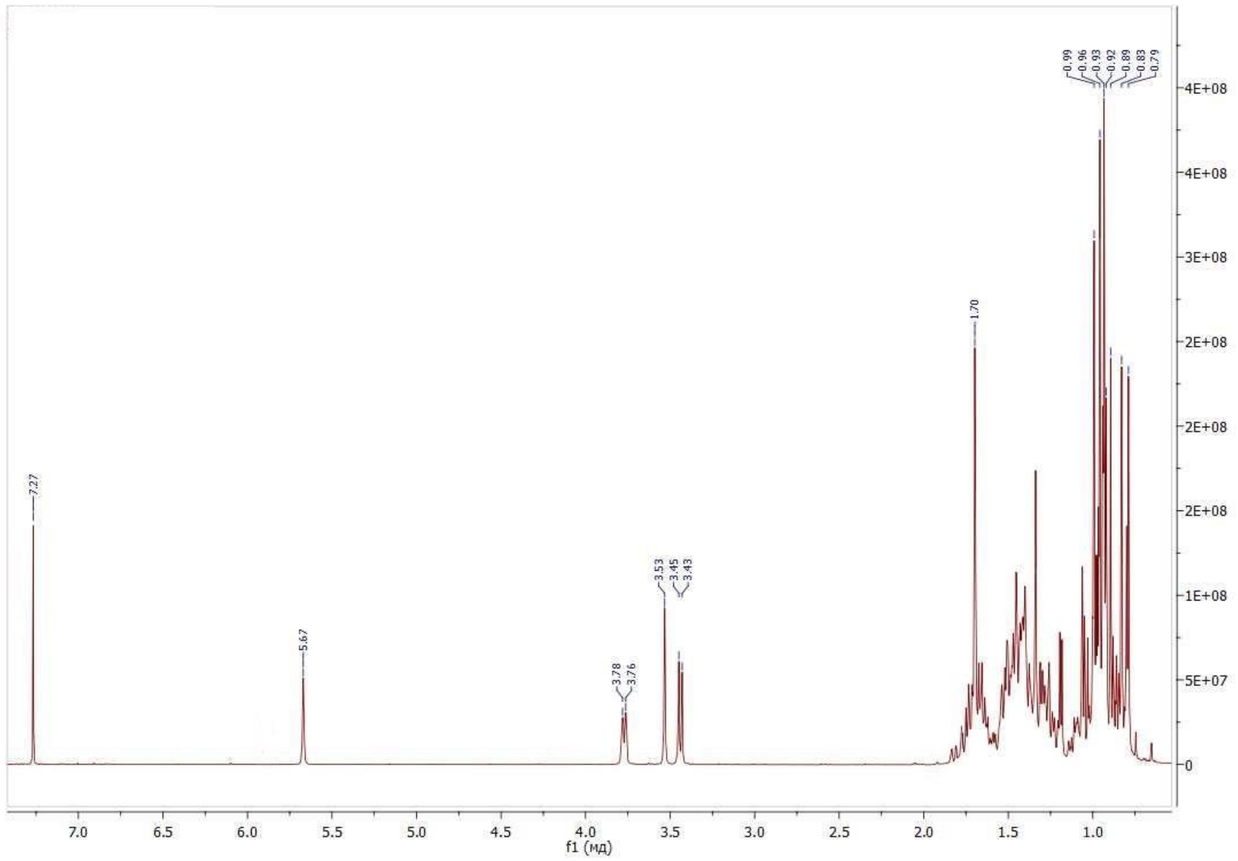

^{13}C NMR (CDCl₃) (3*S*)-3-ethyl-3β-hydroxy-2-methyl-19β,28-epoxy-18α*H*-olean-1-ene (8)

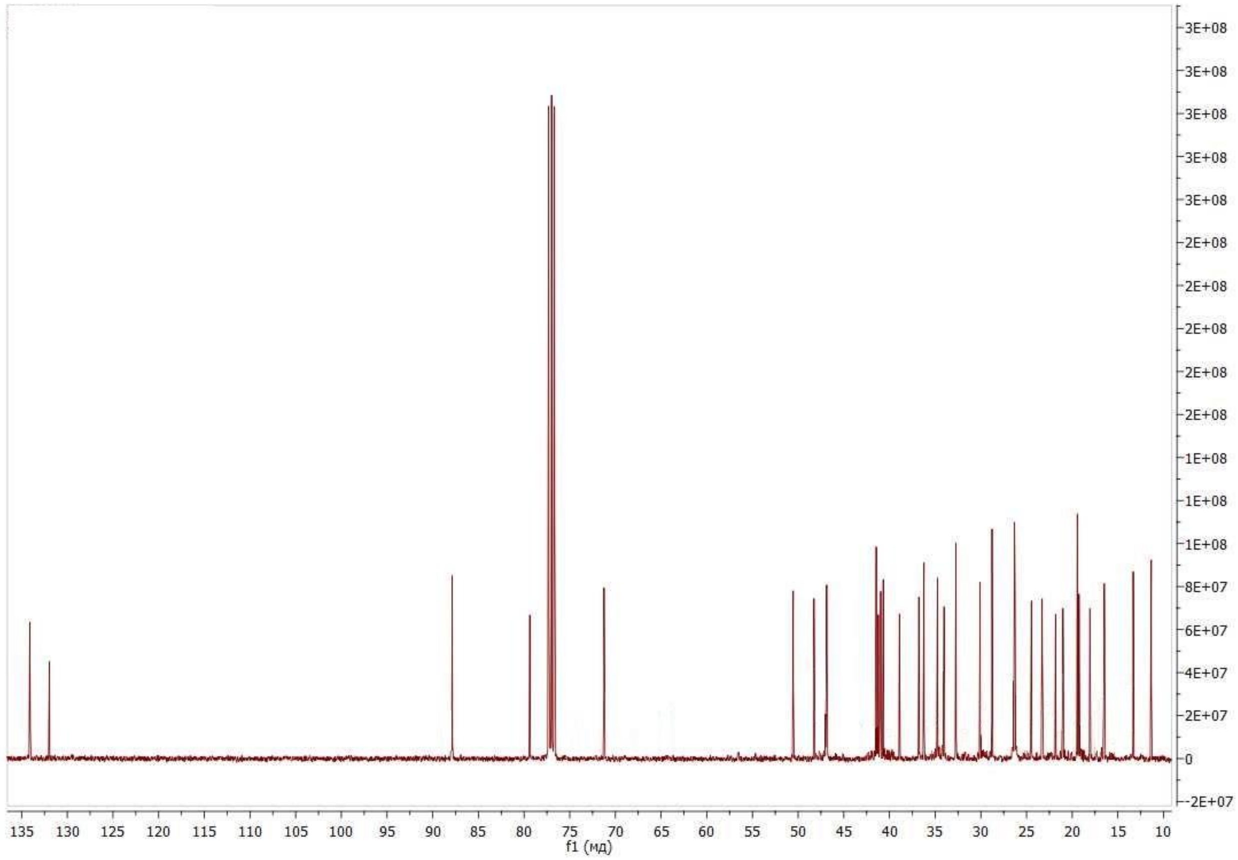

GC-MS (3*S*)-3-ethyl-3β-hydroxy-2-methyl-19β,28-epoxy-18α*H*-olean-1-ene (8)

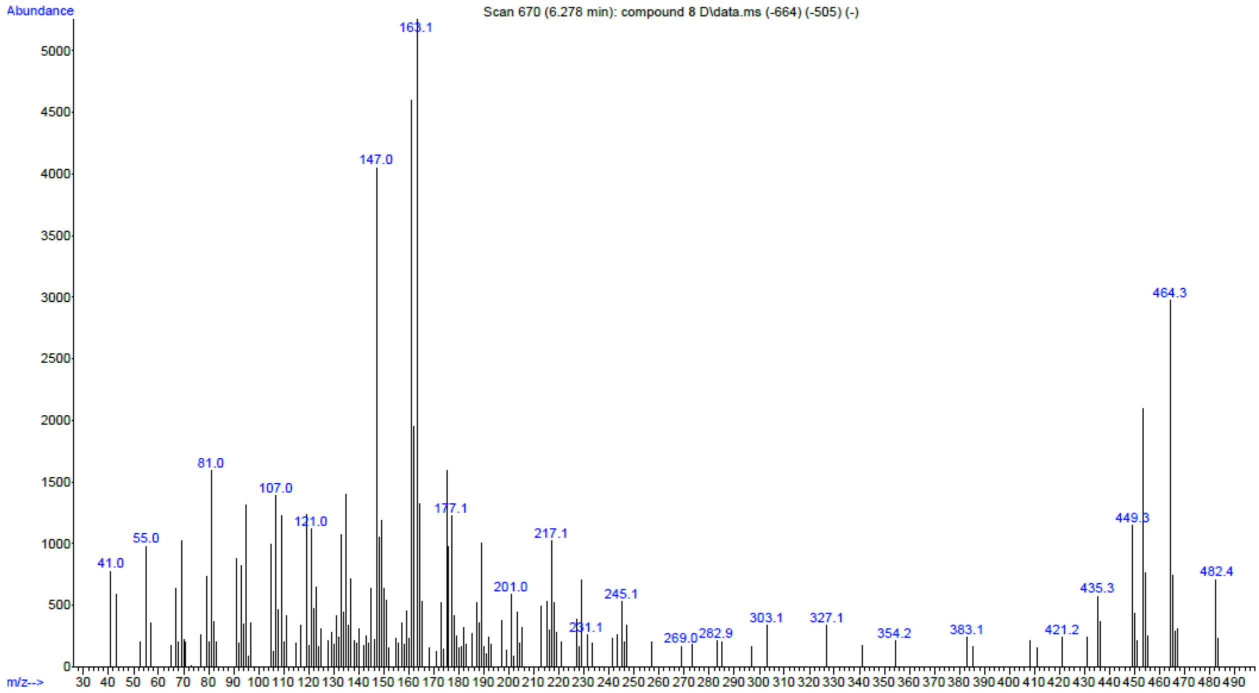

^{1}H NMR (3*R*)-3β-hydroxy-2-methyl-3-phenyl-19β,28-epoxy-18α*H*-olean-1-ene (9)

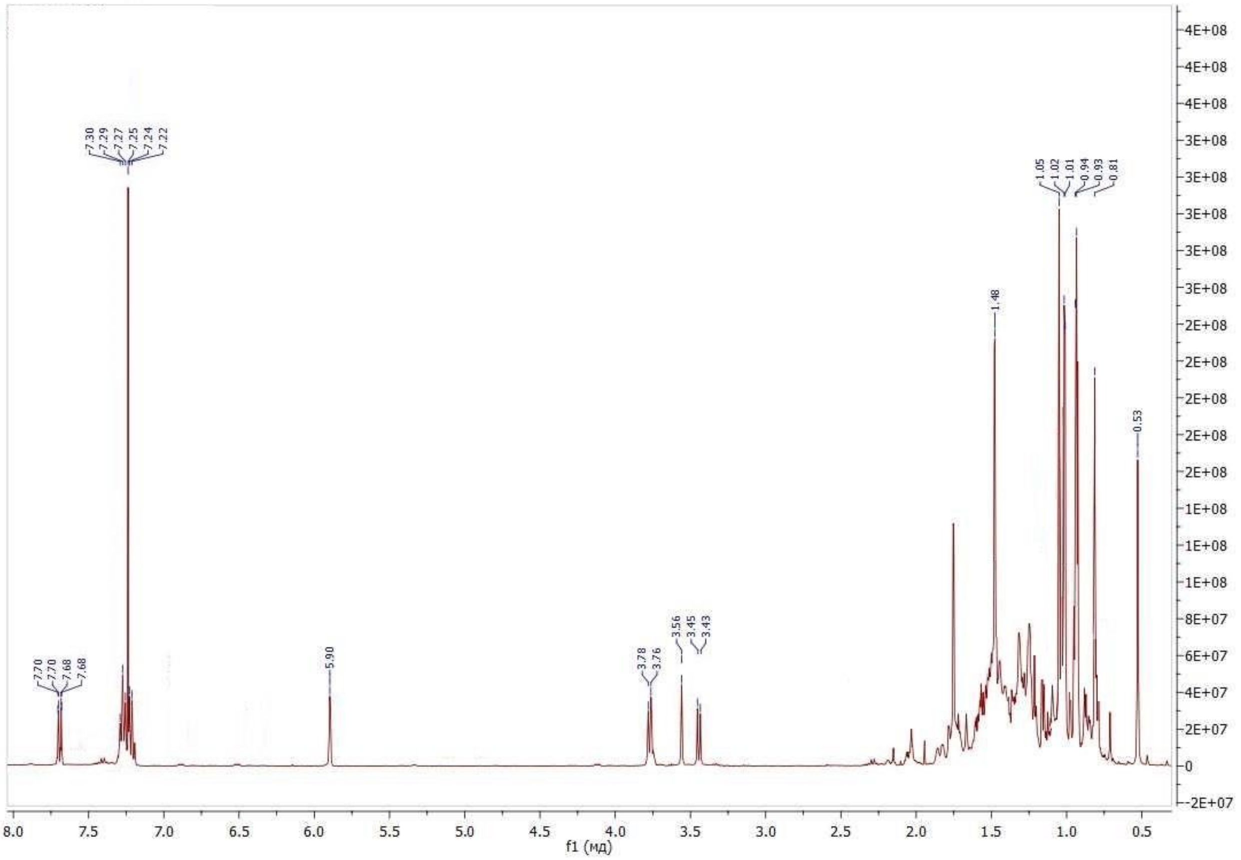

^{13}C NMR ($CDCl_3$) (3*R*)-3β-hydroxy-2-methyl-3-phenyl-19β,28-epoxy-18α*H*-olean-1-ene (9)

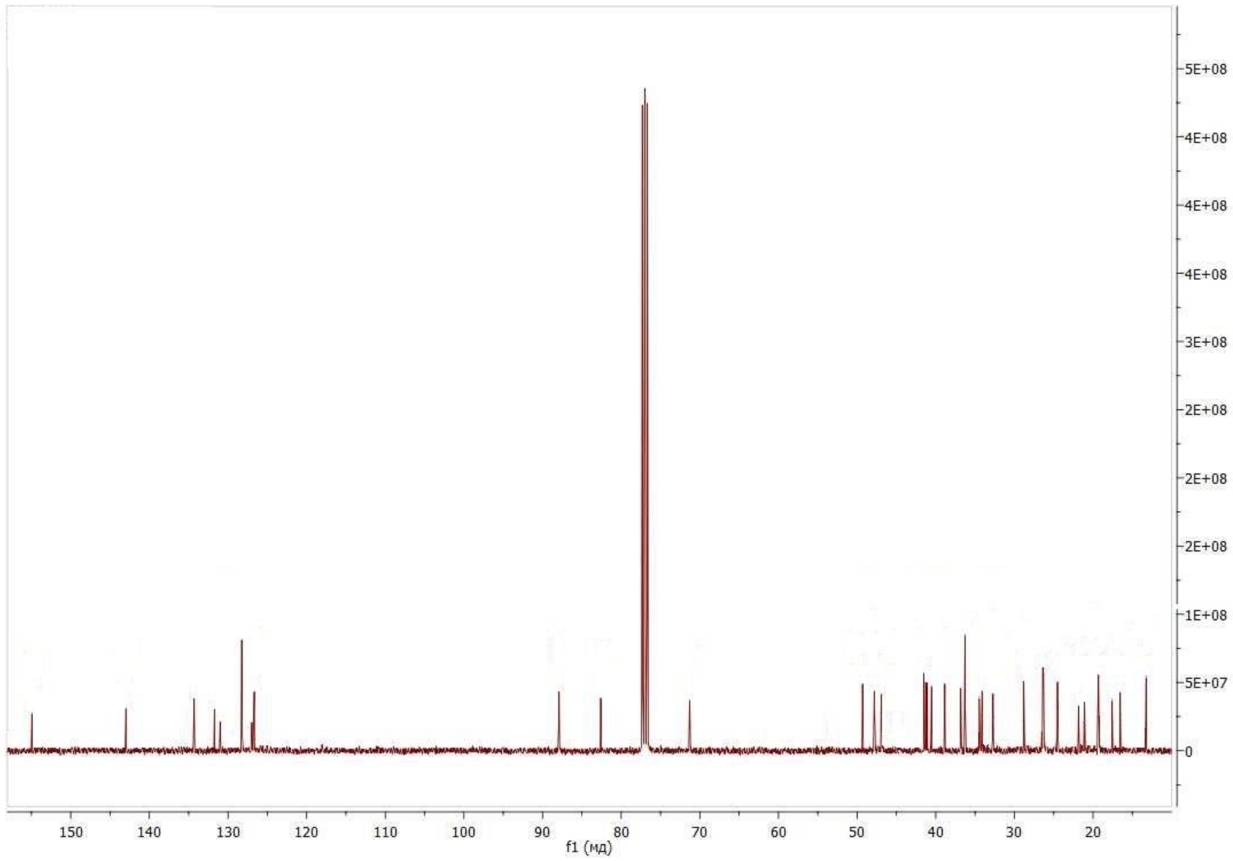

GC-MS (3*R*)-3β-hydroxy-2-methyl-3-phenyl-19β,28-epoxy-18α*H*-olean-1-ene (9)

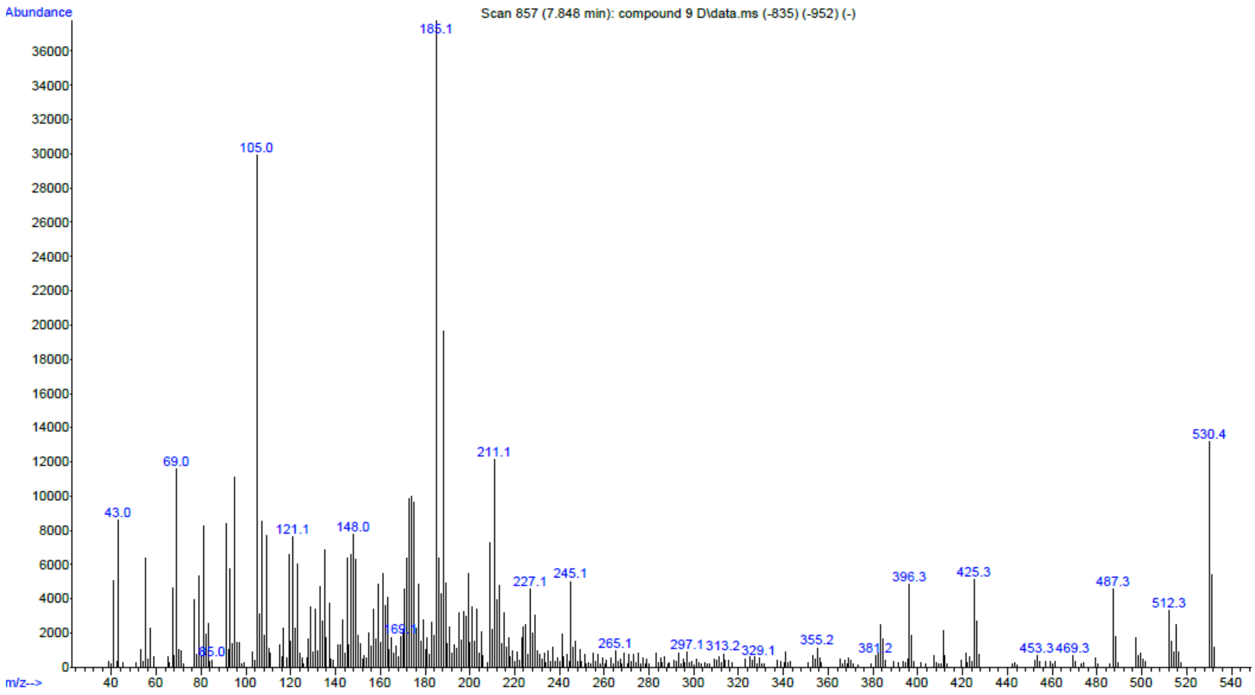

^{1}H NMR (1*S*,2*S*,3*R*)-3β-hydroxy-2,3-dimethyl-(1,2),(19β,28)-diepoxy-18α*H*-oleanane (10)

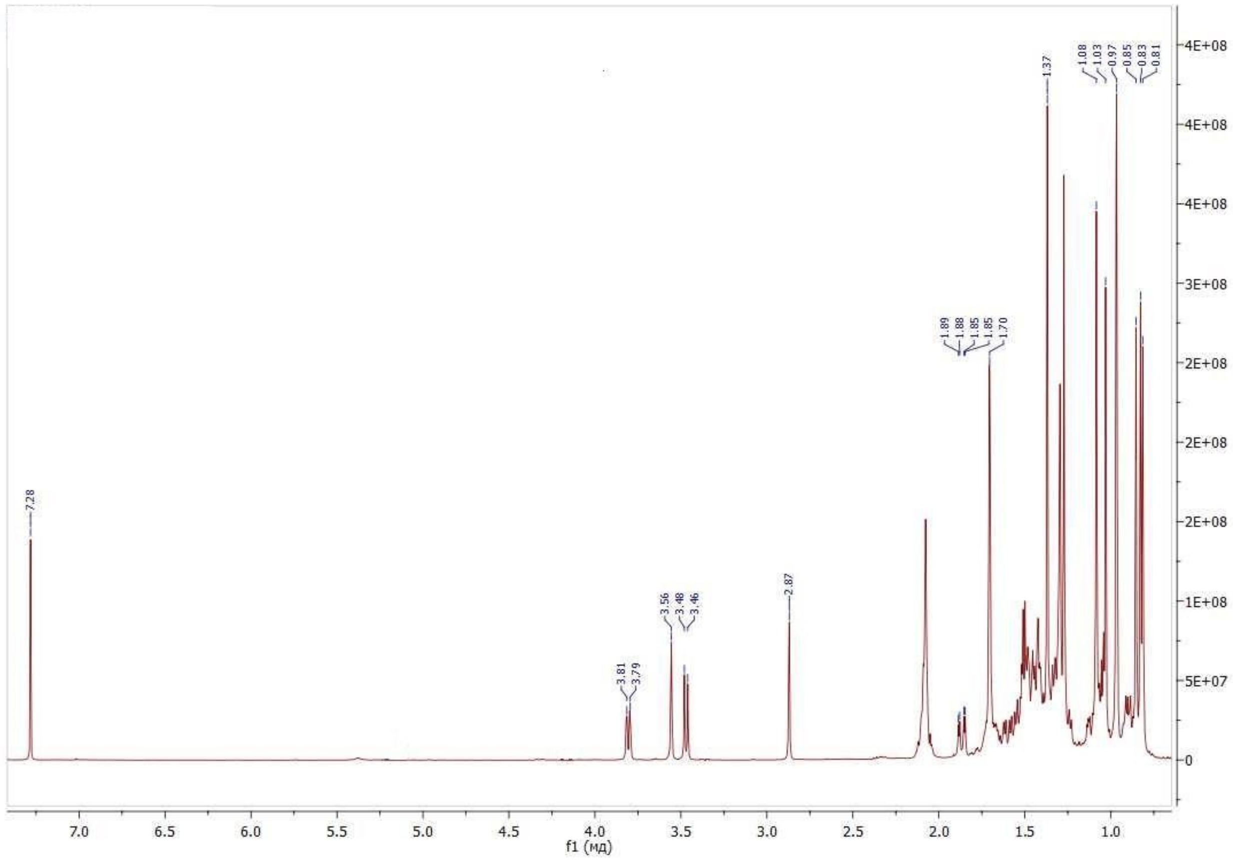

^{13}C NMR ($CDCl_3$) (1*S*,2*S*,3*R*)-3β-hydroxy-2,3-dimethyl-(1,2),(19β,28)-diepoxy-18α*H*-oleanane (10)

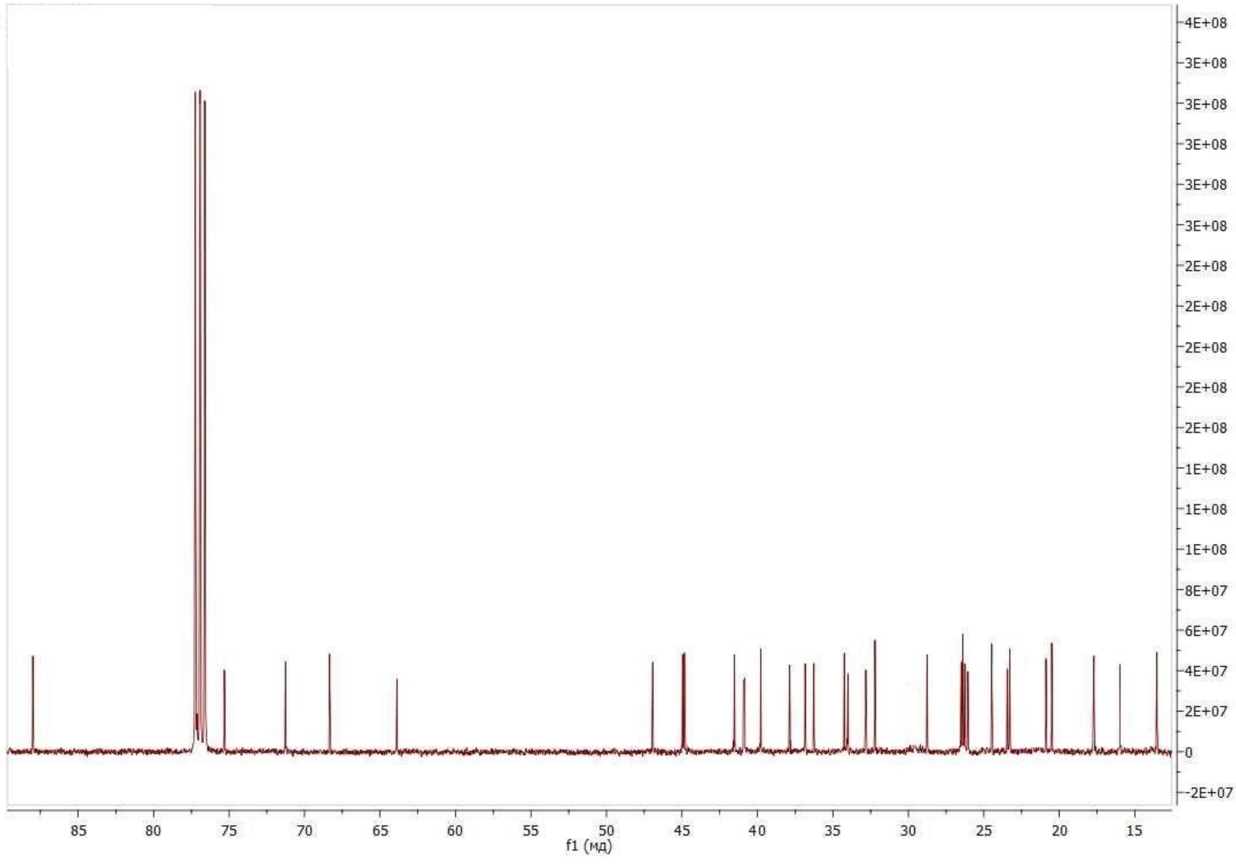

^{1}H–^{1}H COSY ($CDCl_3$) (1*S*,2*S*,3*R*)-3β-hydroxy-2,3-dimethyl-(1,2),(19β,28)-diepoxy-18α*H*-oleanane (10)

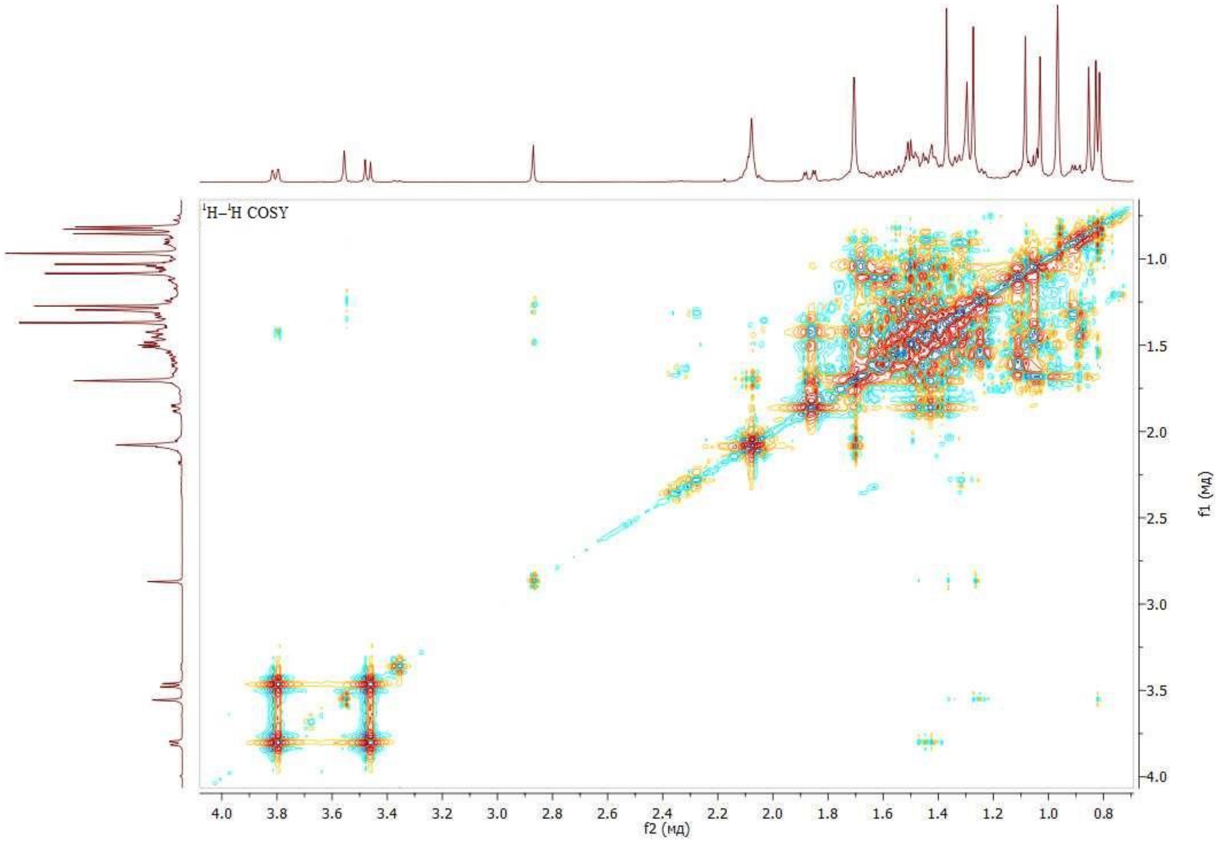

NOESY ($CDCl_3$) (1*S*,2*S*,3*R*)-3β-hydroxy-2,3-dimethyl-(1,2),(19β,28)-diepoxy-18α*H*-oleanane (10)

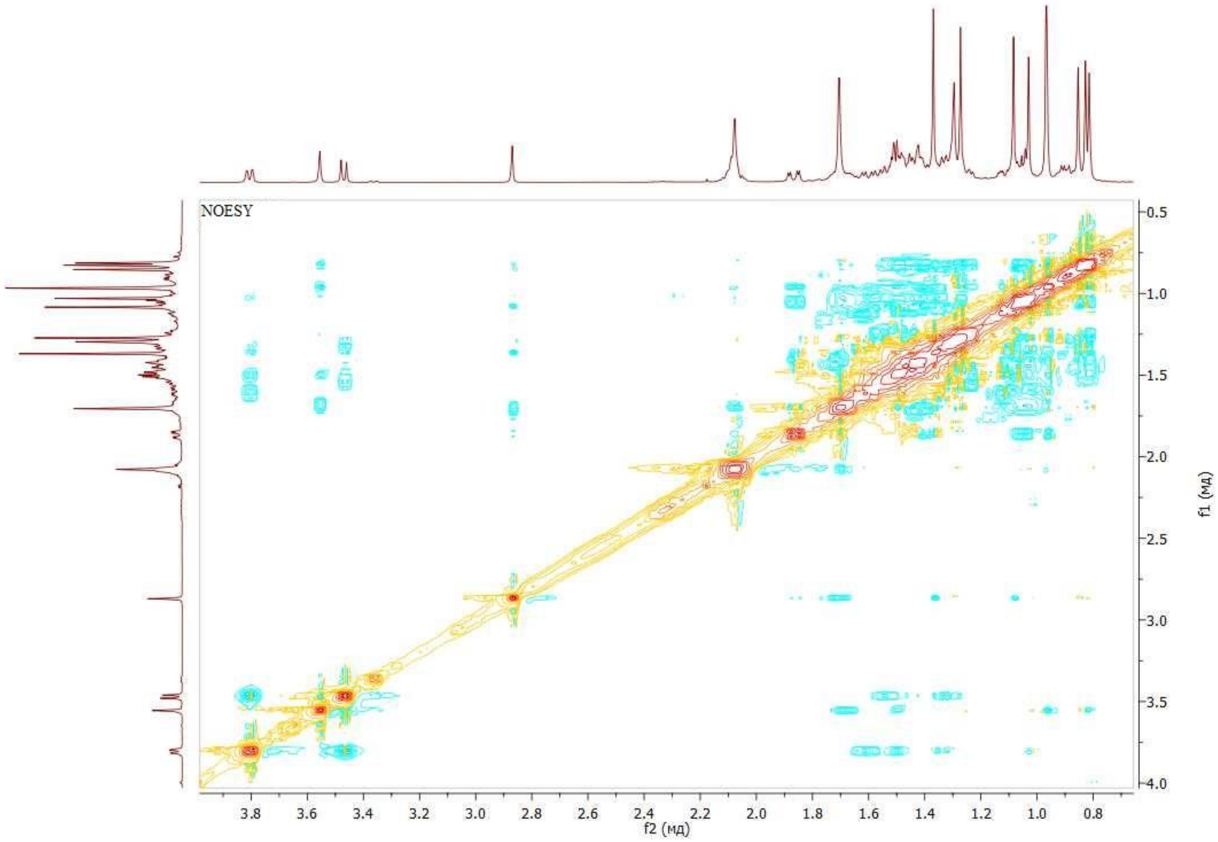

HMBC ($CDCl_3$) (1*S*,2*S*,3*R*)-3β-hydroxy-2,3-dimethyl-(1,2),(19β,28)-diepoxy-18α*H*-oleanane (10)

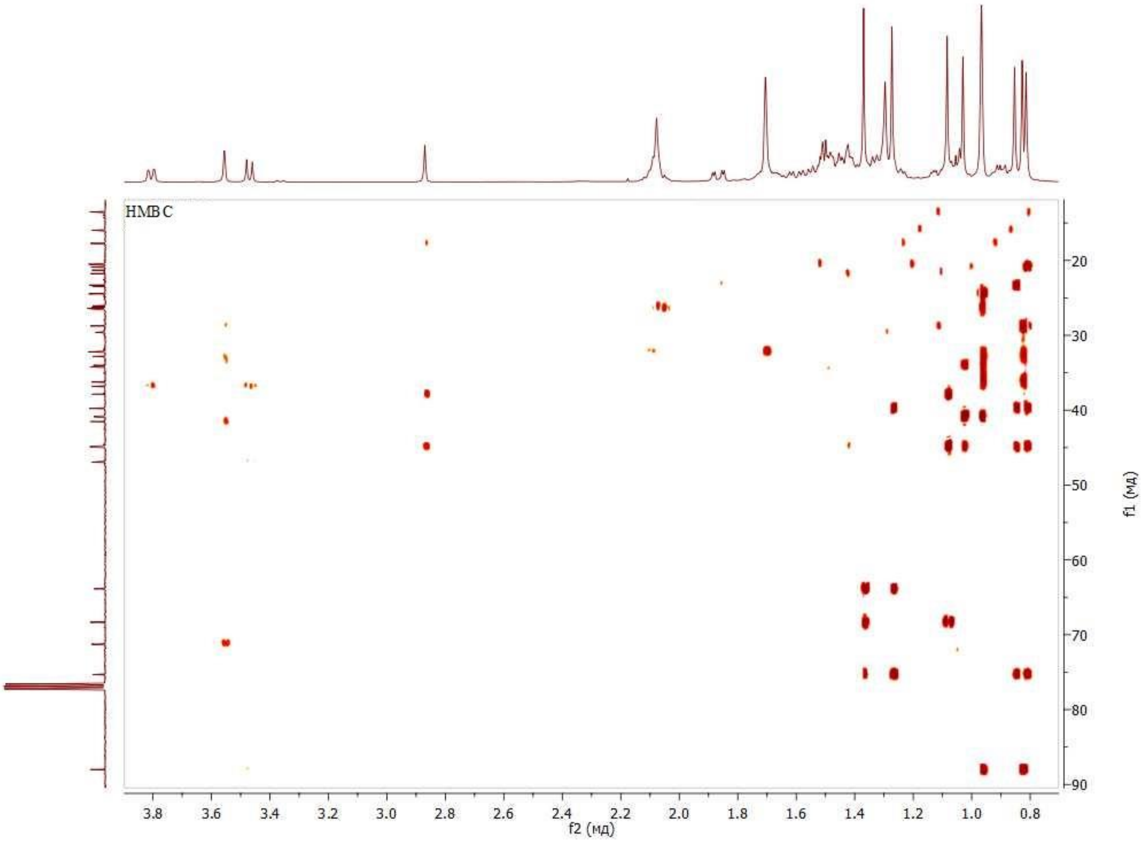

HSQC (CDCl$_3$) (1*S*,2*S*,3*R*)-3β-hydroxy-2,3-dimethyl-(1,2),(19β,28)-diepoxy-18α*H*-oleanane (10)

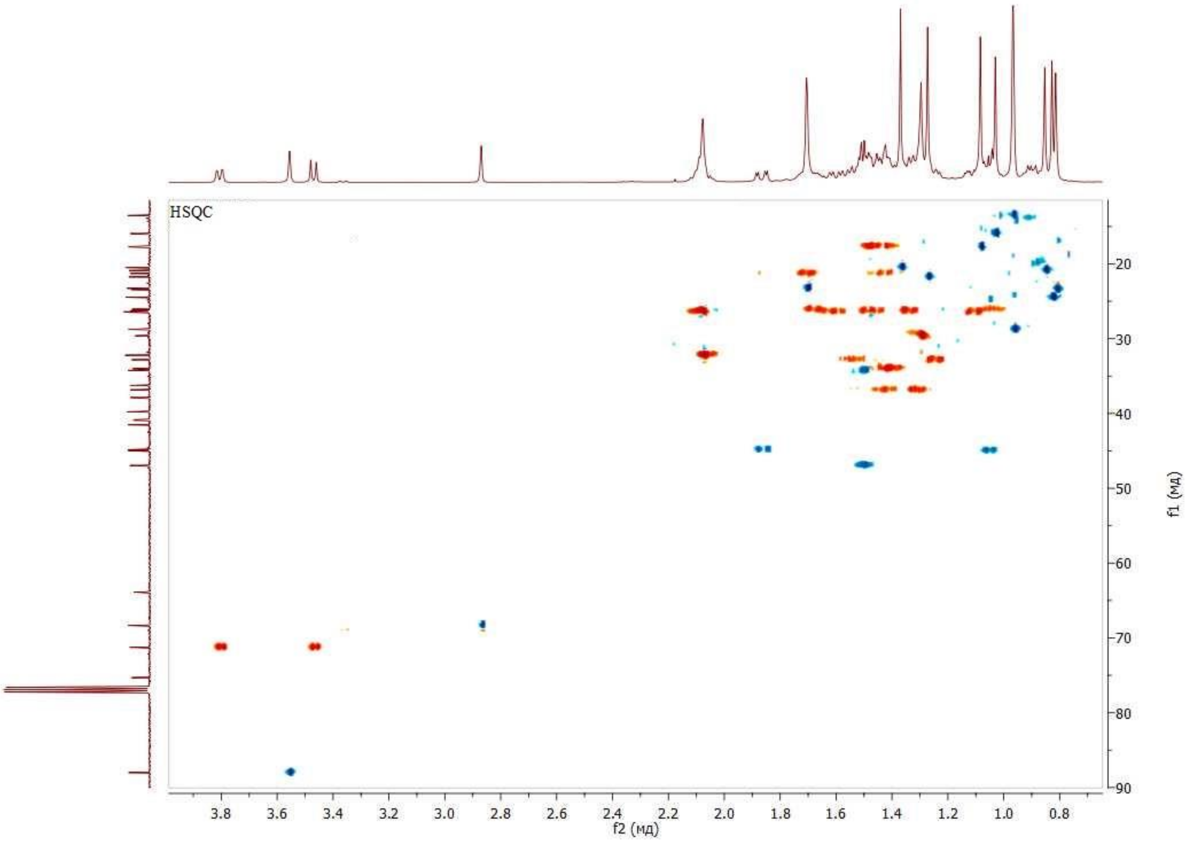

GC-MS (1*S*,2*S*,3*R*)-3β-hydroxy-2,3-dimethyl-(1,2),(19β,28)-diepoxy-18α*H*-oleanane (10)

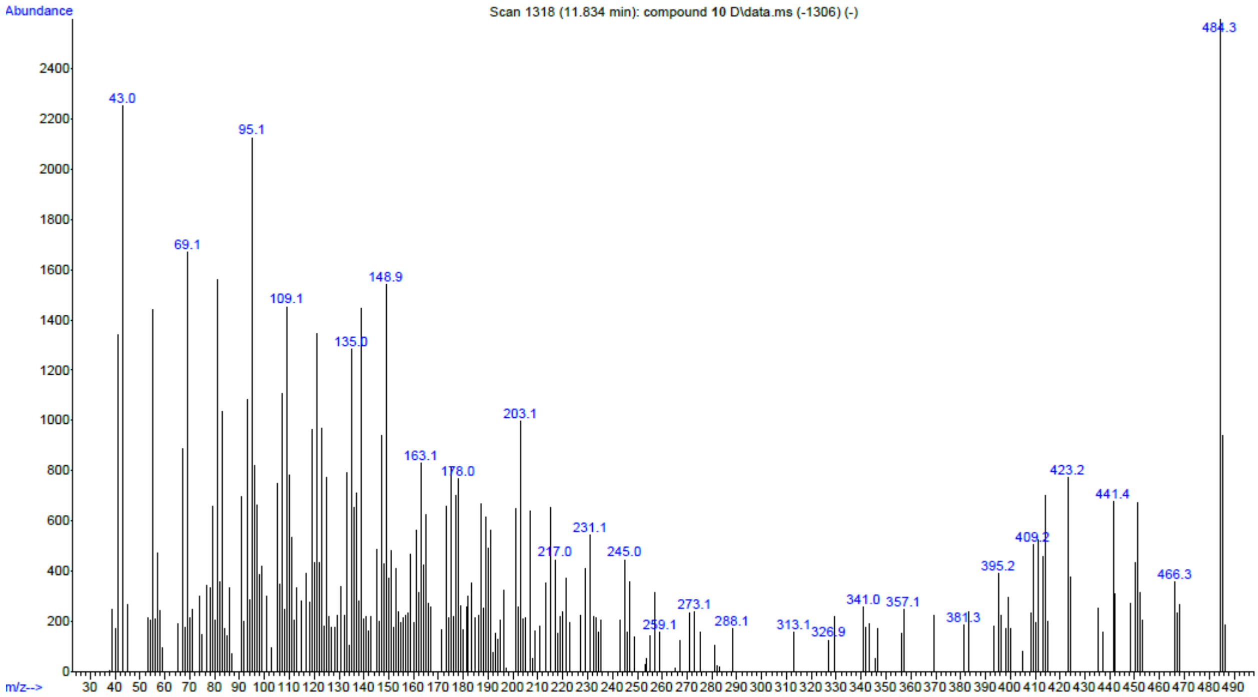

^{1}H NMR (1*S*,2*S*,3*R*)-3-ethyl-3β-hydroxy-2-methyl-(1,2),(19β,28)-diepoxy-18α*H*-oleanane (11)

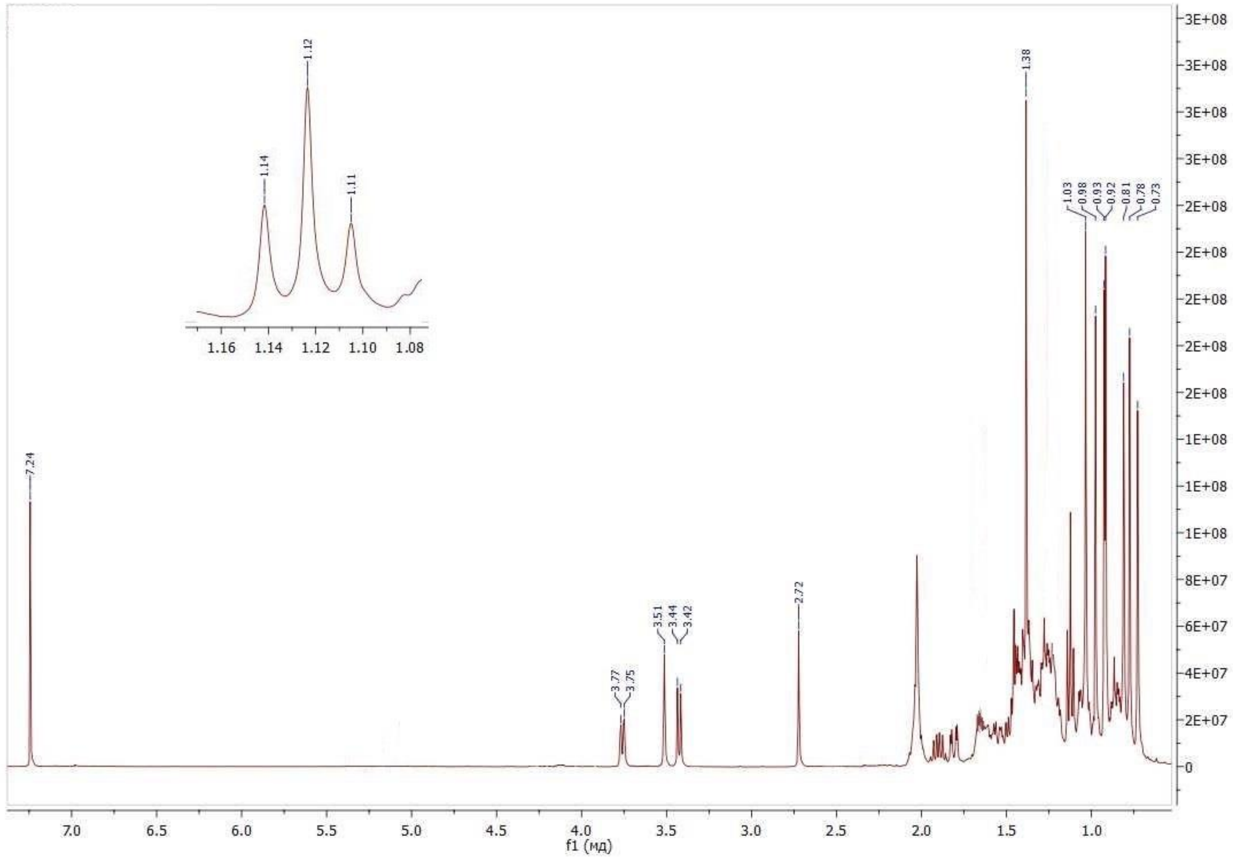

^{13}C NMR ($CDCl_3$) (1*S*,2*S*,3*R*)-3-ethyl-3β-hydroxy-2-methyl-(1,2),(19β,28)-diepoxy-18α*H*-oleanane (11)

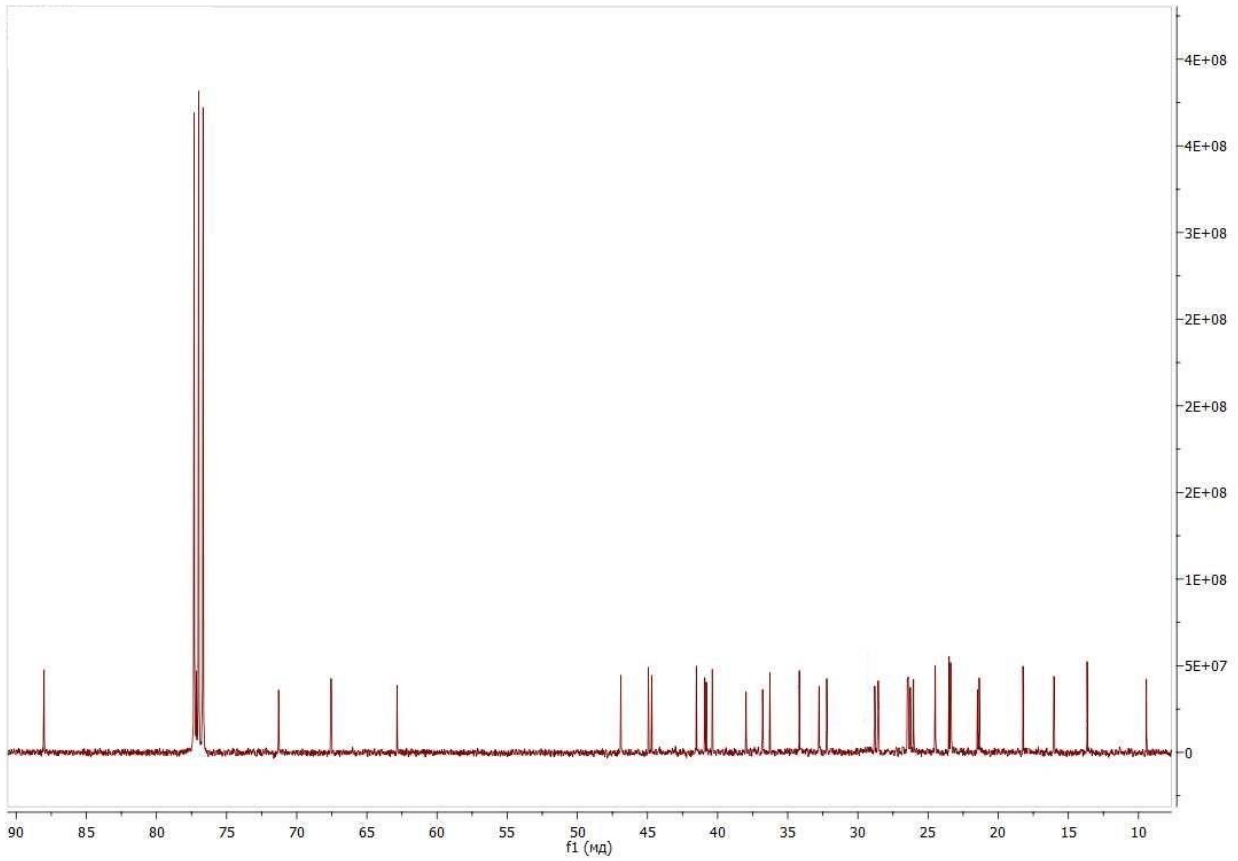

GC-MS (1*S*,2*S*,3*R*)-3-ethyl-3β-hydroxy-2-methyl-(1,2),(19β,28)-diepoxy-18α*H*-oleanane (11)

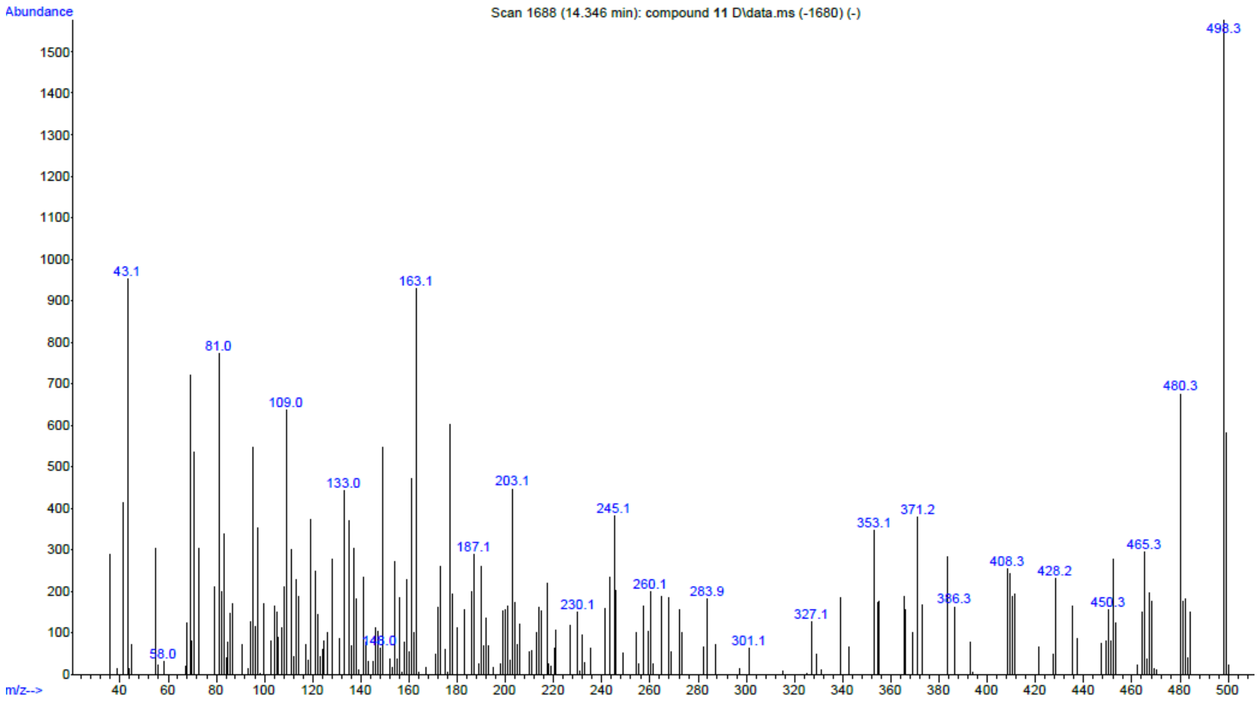

^{1}H NMR (1*S*,2*S*,3*R*)-3β-hydroxy-2-methyl-3-phenyl-(1,2),(19β,28)-diepoxy-18α*H*-oleanane (12)

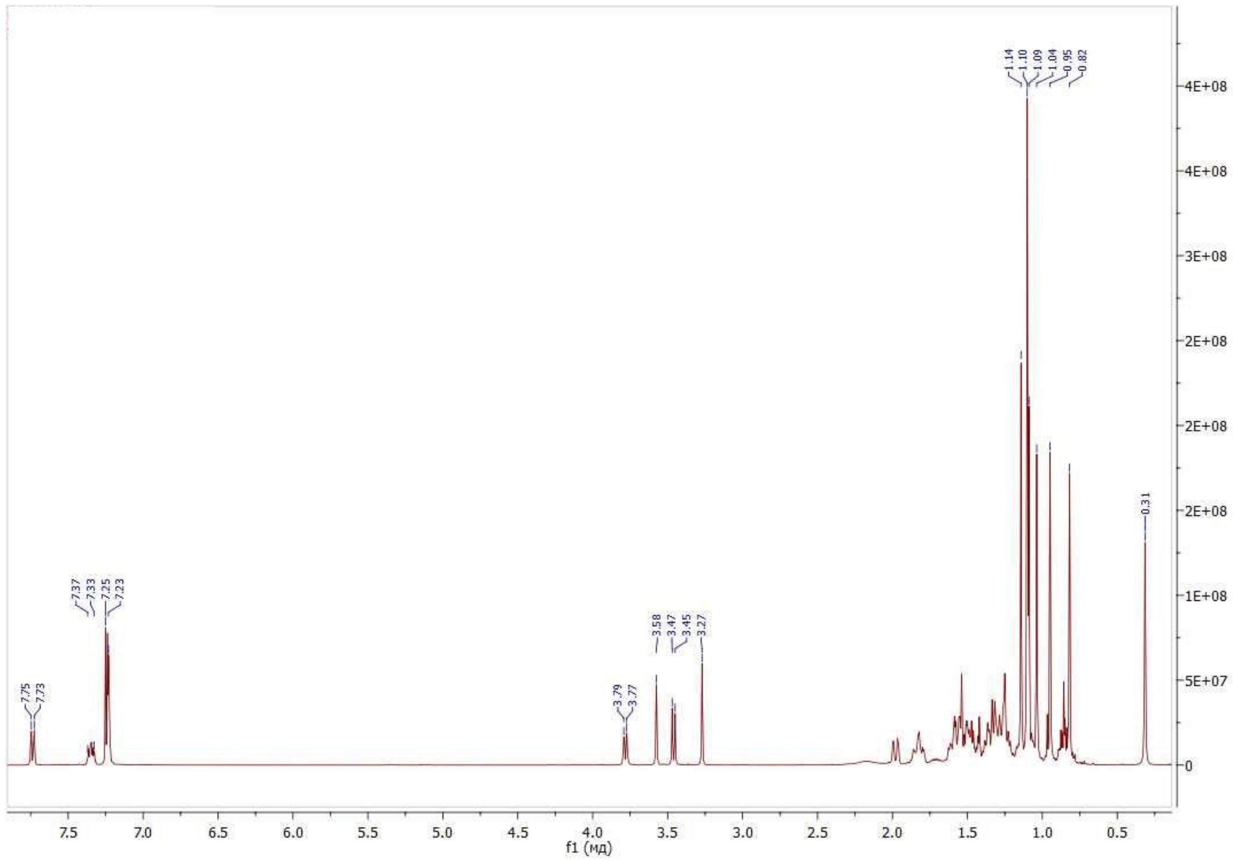

^{13}C NMR ($CDCl_3$) (1*S*,2*S*,3*R*)-3β-hydroxy-2-methyl-3-phenyl-(1,2),(19β,28)-diepoxy-18α*H*-oleanane (12)

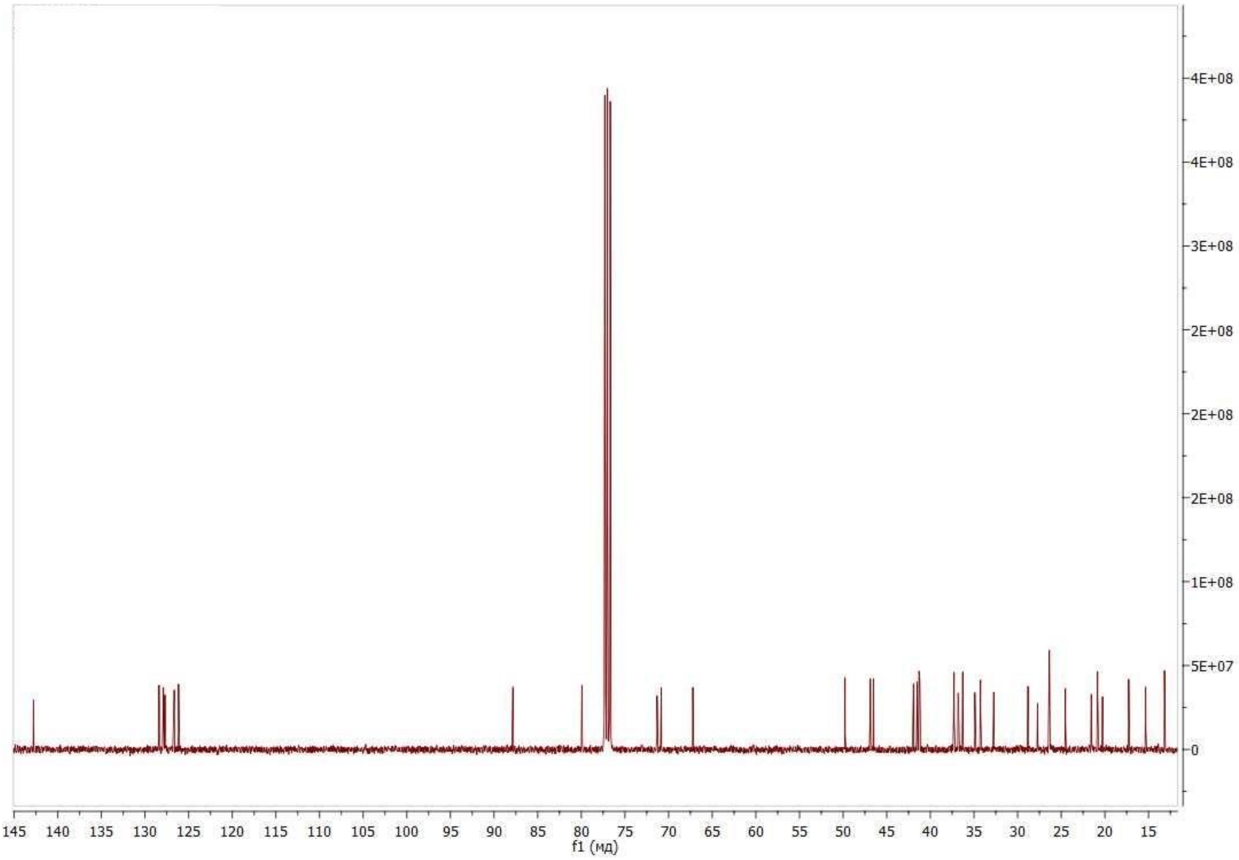

GC-MS (1*S*,2*S*,3*R*)-3β-hydroxy-2-methyl-3-phenyl-(1,2),(19β,28)-diepoxy-18α*H*-oleanane (12)

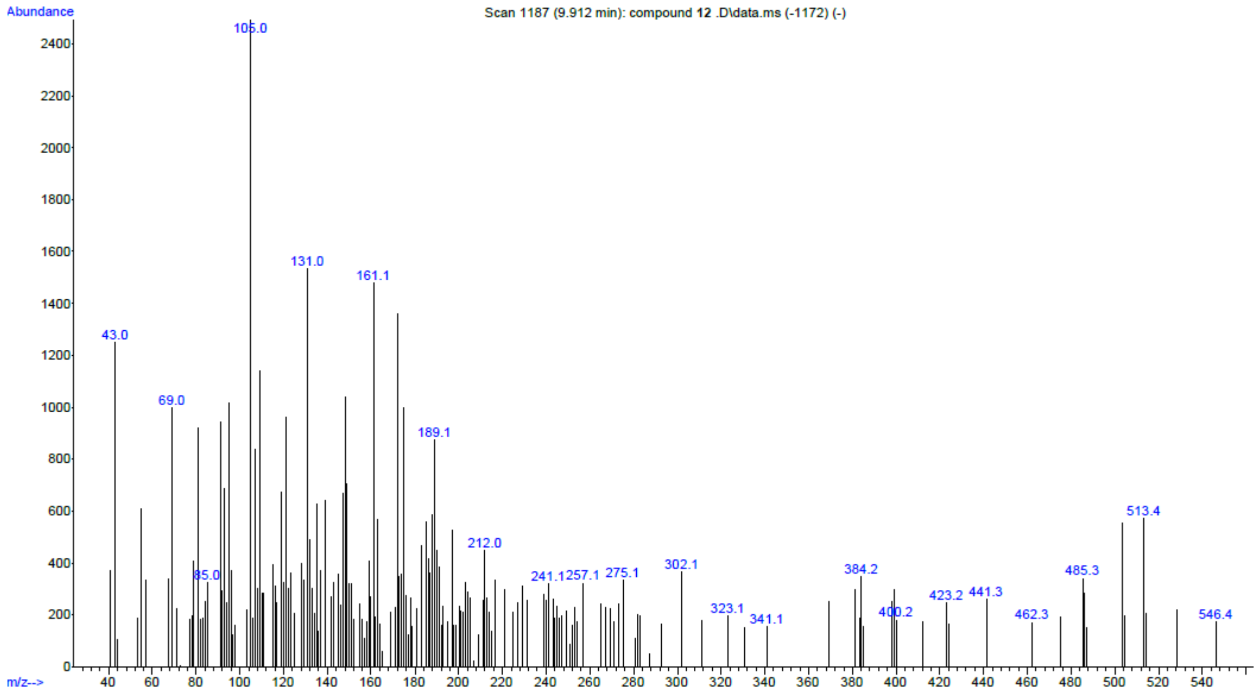

^{1}H NMR (1*S*,2*S*)-1α-hydroxy-2-methyl-3-oxo-19β,28-epoxy-18α*H*-oleanane (13)

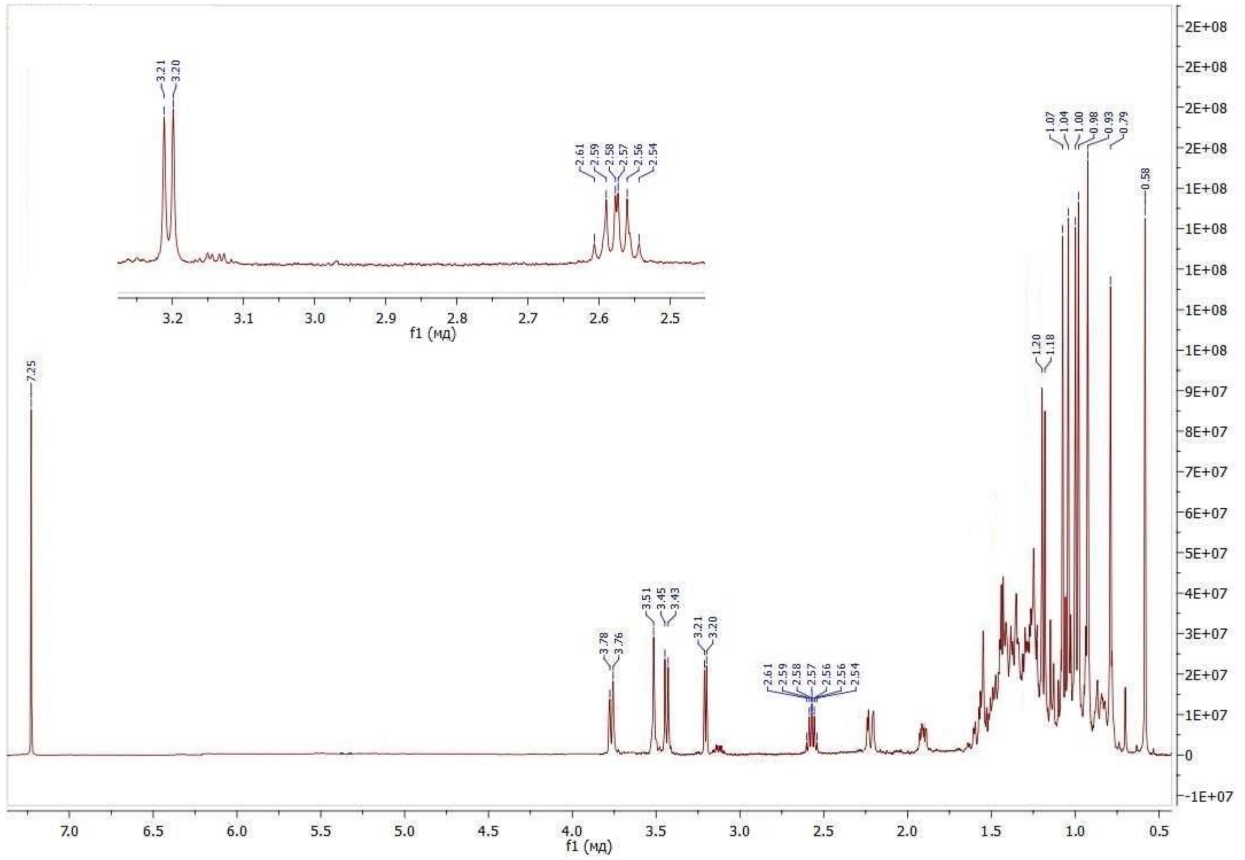

^{13}C NMR ($CDCl_3$) (1*S*,2*S*)-1α-hydroxy-2-methyl-3-oxo-19β,28-epoxy-18α*H*-oleanane (13)

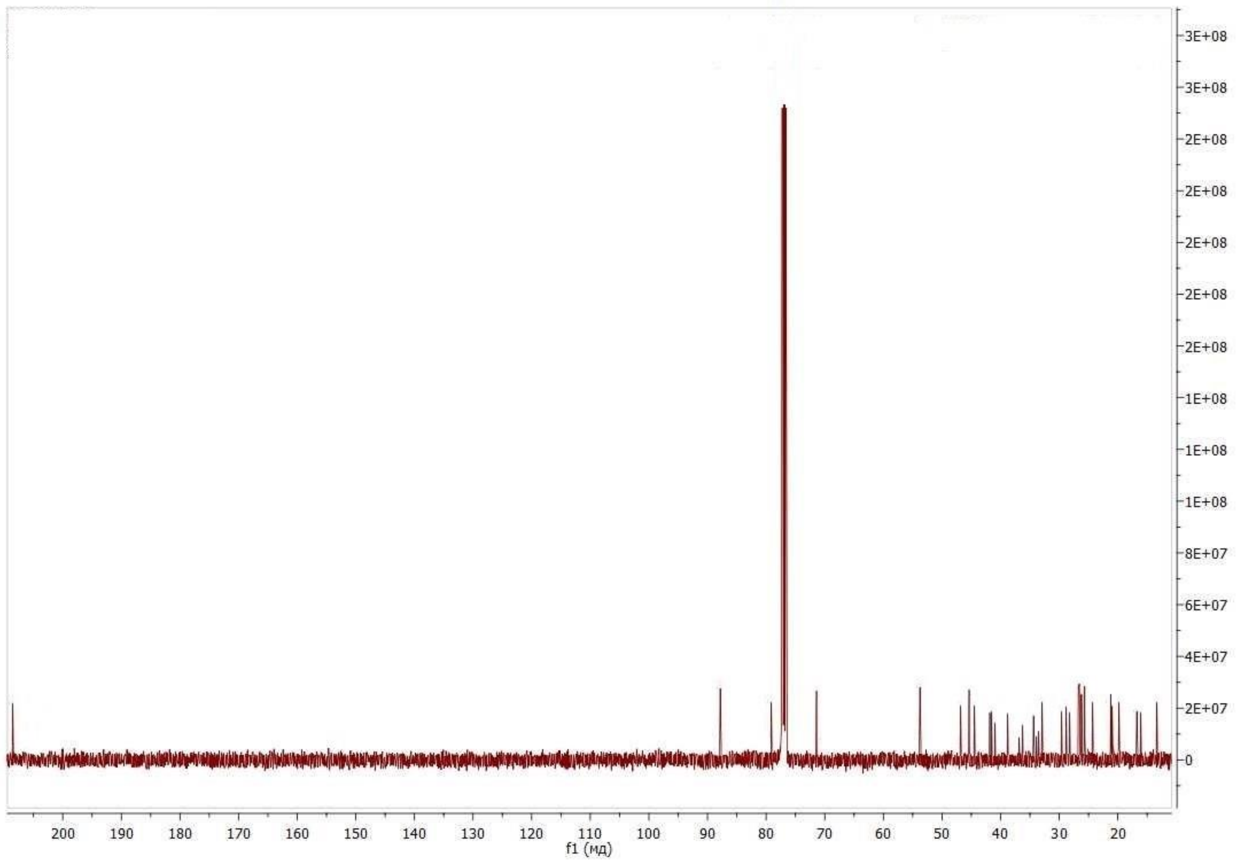

GC-MS (1*S*,2*S*)-1α-hydroxy-2-methyl-3-oxo-19β,28-epoxy-18α*H*-oleanane (13)

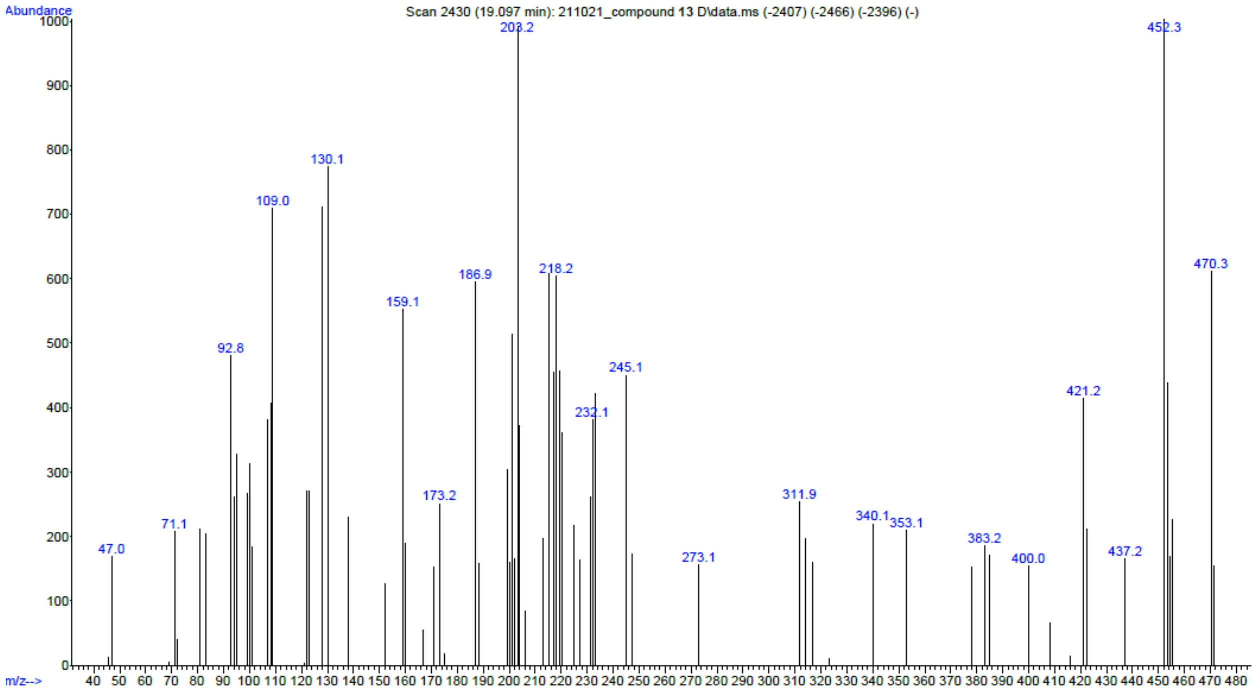

^{1}H NMR (1*S*,2*R*)-1α-hydroxy-2-methyl-3-oxo-19β,28-epoxy-18α*H*-olean-1-ene (14)

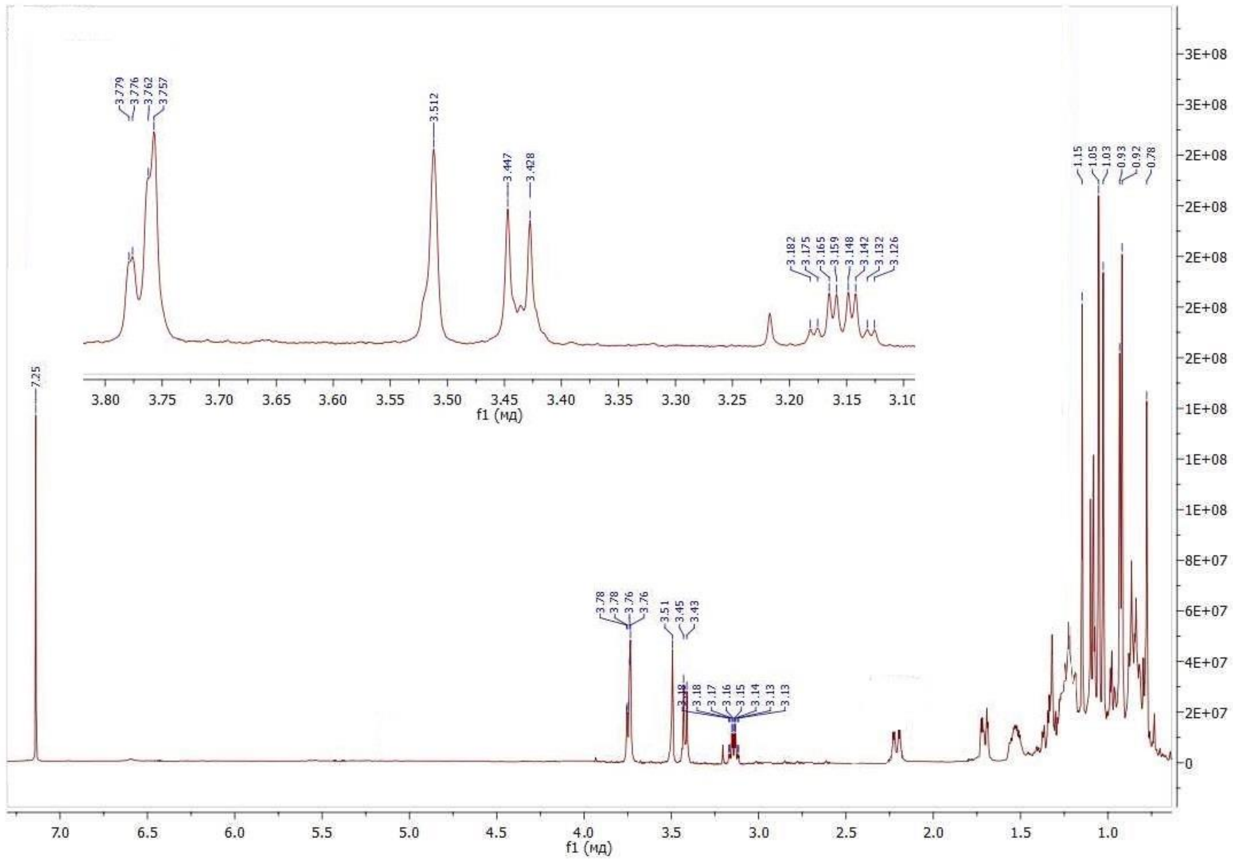

^{13}C NMR (CDCl₃) (1*S*,2*R*)-1α-hydroxy-2-methyl-3-oxo-19β,28-epoxy-18α*H*-olean-1-ene (14)

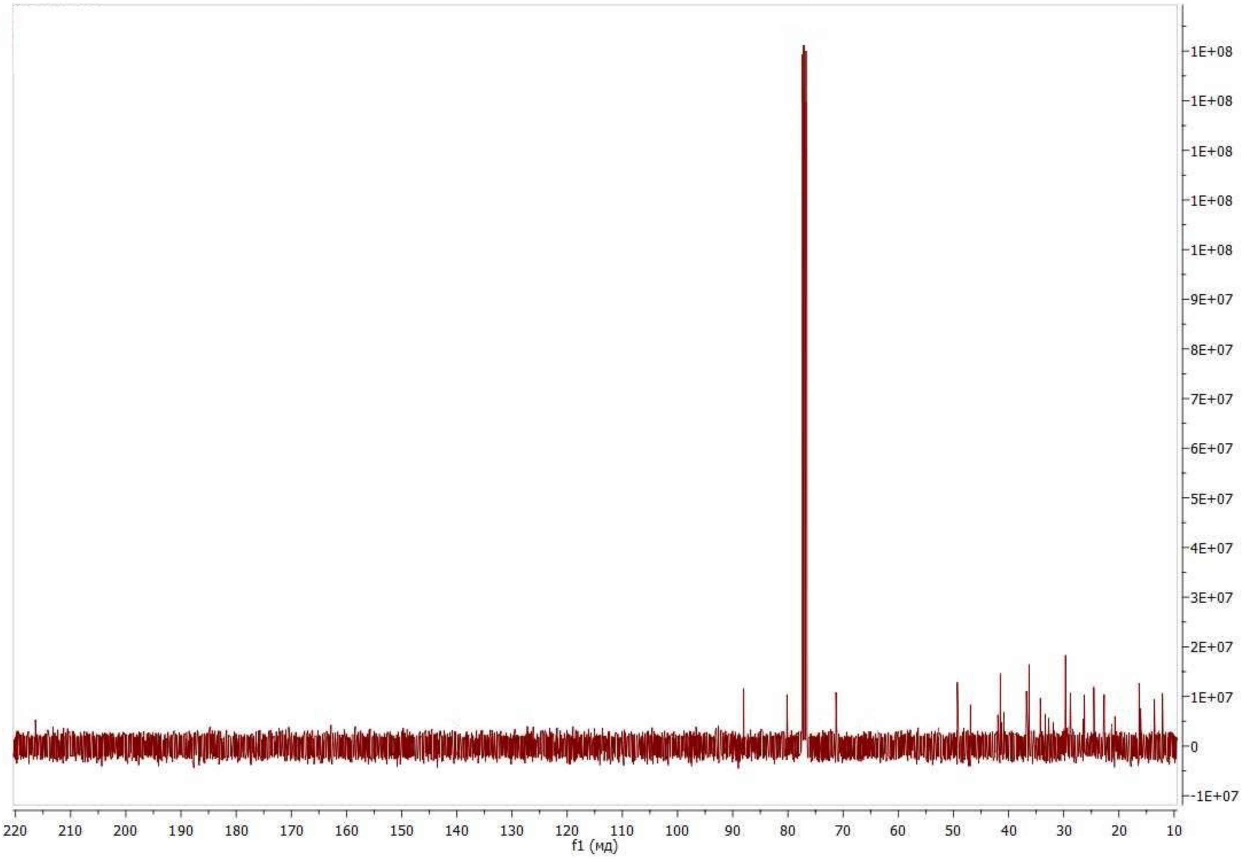

GC-MS (1*S*,2*R*)-1α-hydroxy-2-methyl-3-oxo-19β,28-epoxy-18α*H*-olean-1-ene (14)

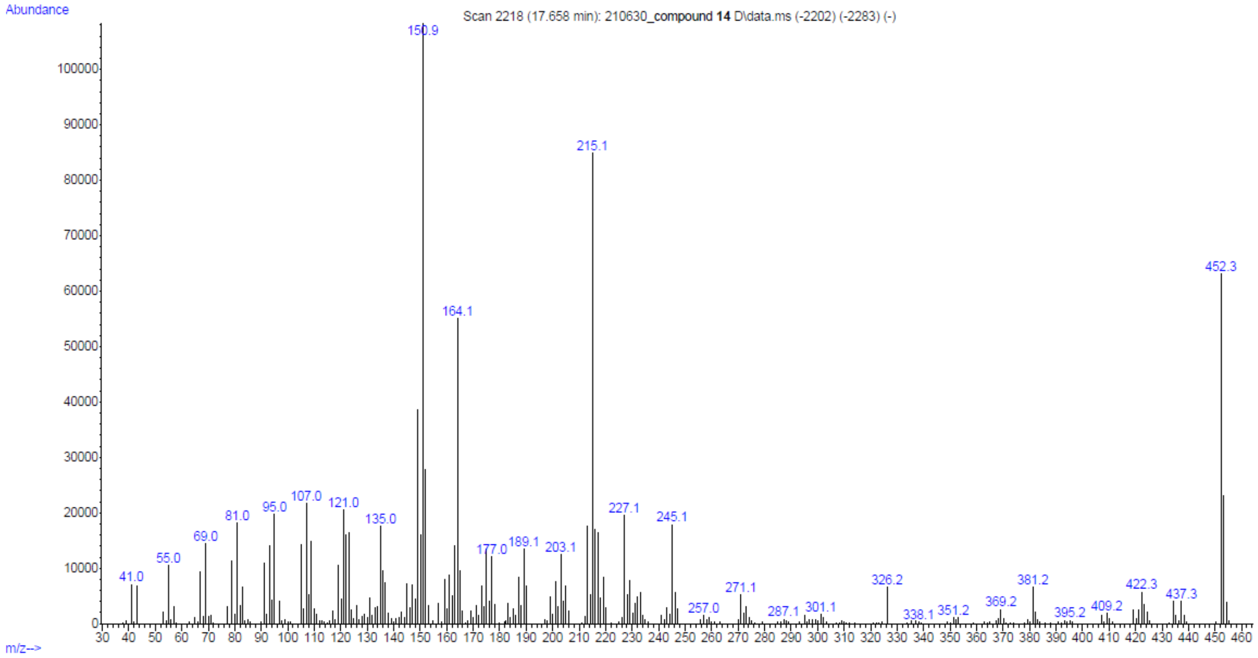

^{1}H NMR (1*S*,3*S*)-1α,3β-dihydroxy-3-ethyl-2-methylene-19β,28-epoxy-18α*H*-oleanane (15)

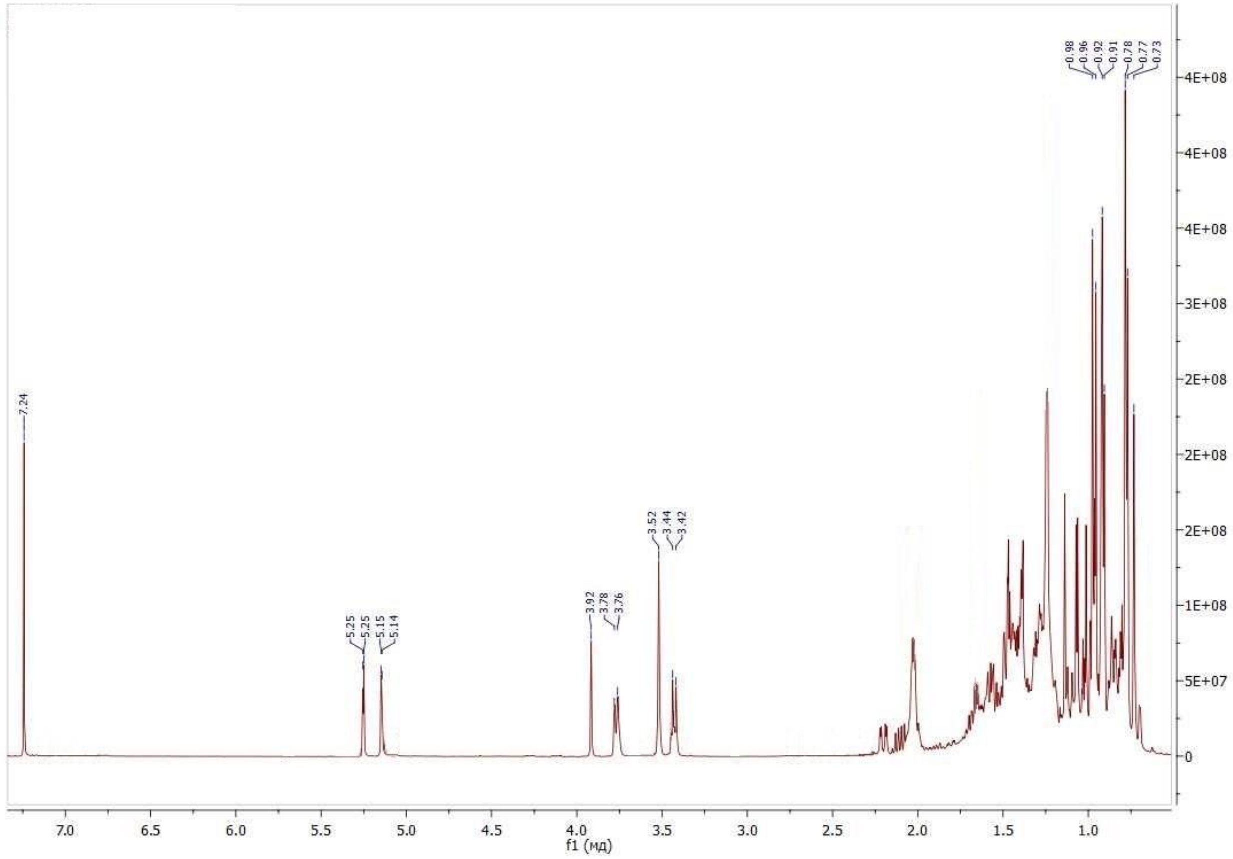

^{13}C NMR ($CDCl_3$) (1*S*,3*S*)-1α,3β-dihydroxy-3-ethyl-2-methylene-19β,28-epoxy-18α*H*-oleanane (15)

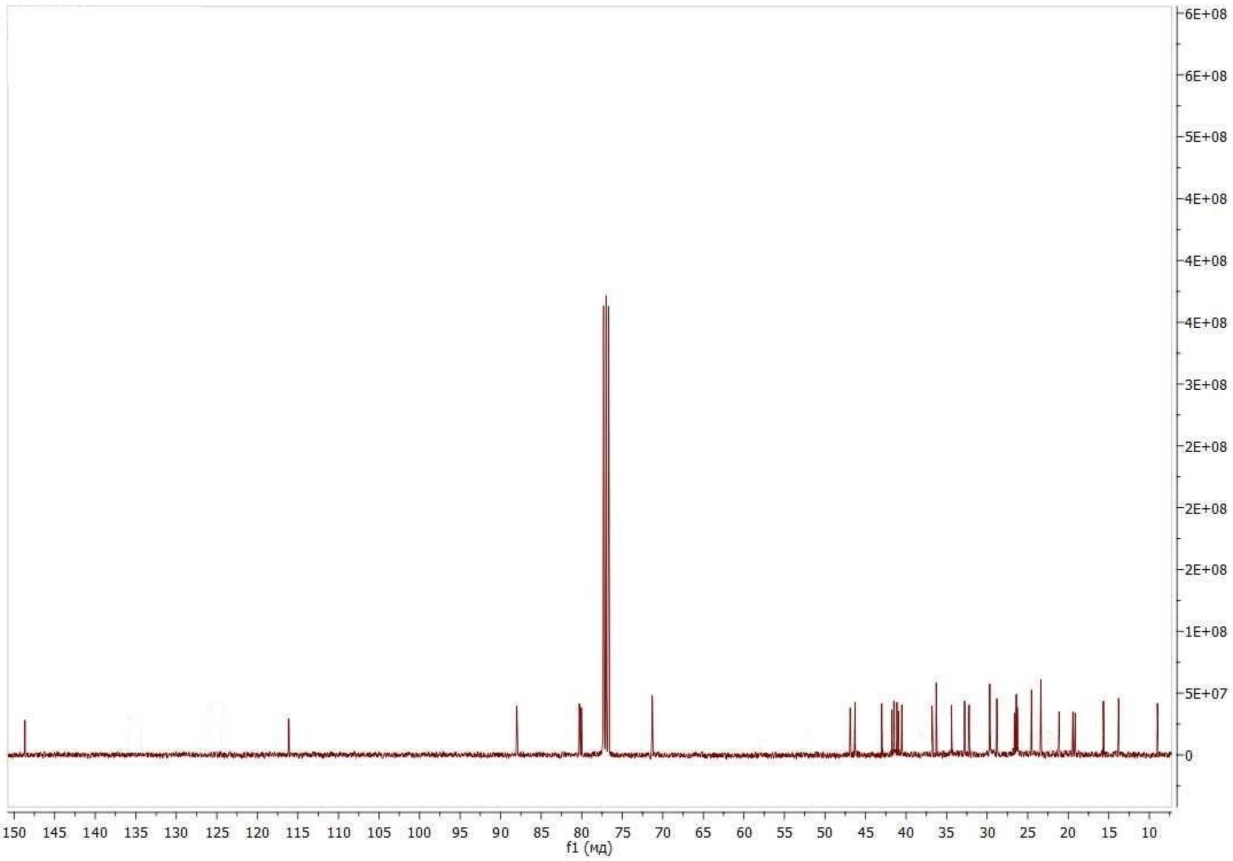

GC-MS (1*S*,3*S*)-1α,3β-dihydroxy-3-ethyl-2-methylene-19β,28-epoxy-18α*H*-oleanane (15)

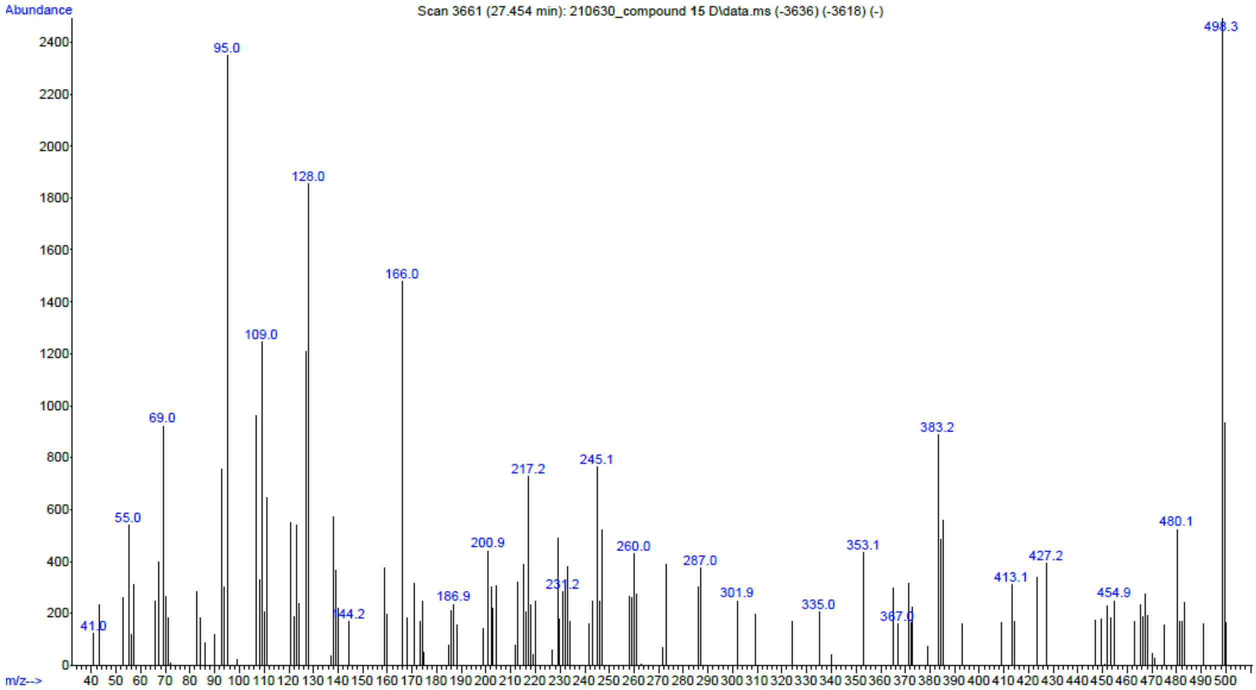

^{1}H NMR (2*S*,3*S*)-2,3-dimethyl-(2,3),(19β,28)-diepoxy-18α*H*-olean-1-one (16)

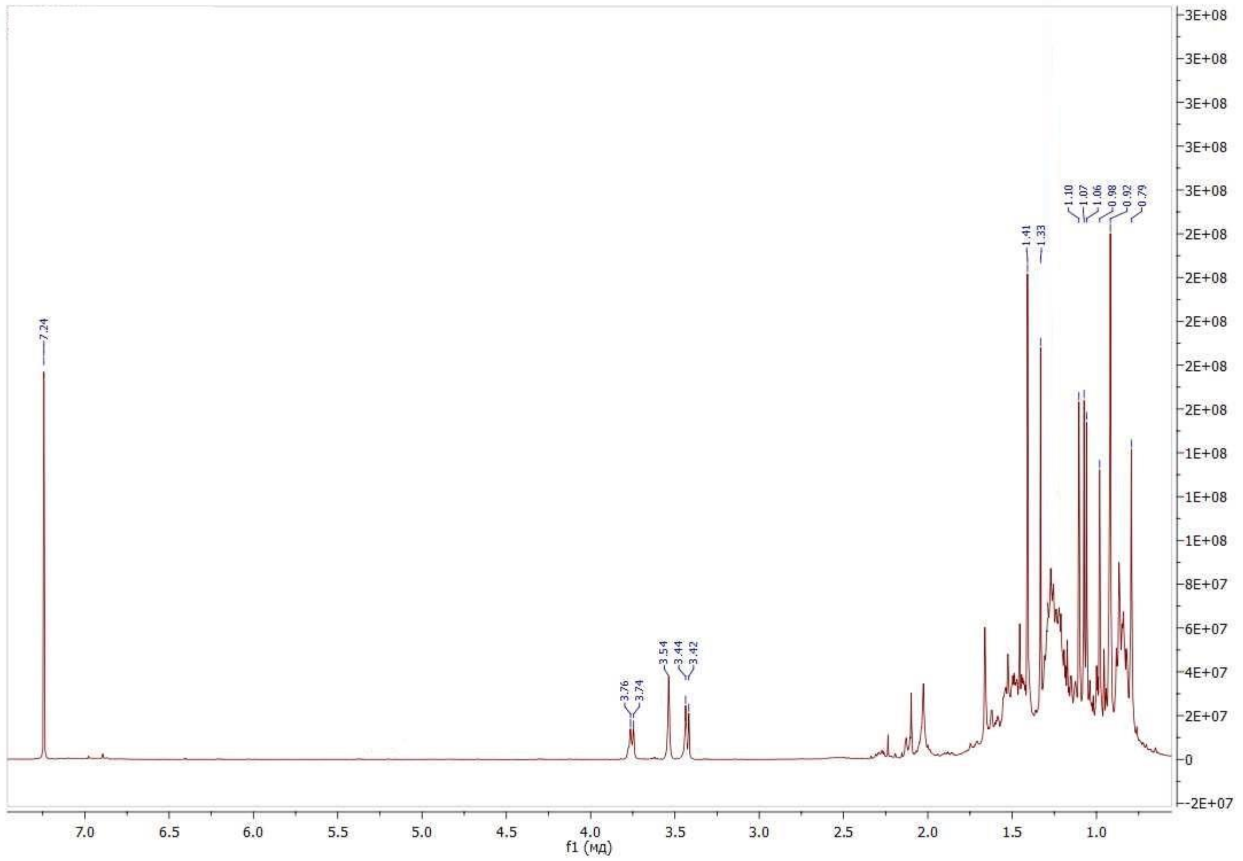

^{13}C NMR ($CDCl_3$) (2*S*,3*S*)-2,3-dimethyl-(2,3),(19β,28)-diepoxy-18α*H*-olean-1-one (16)

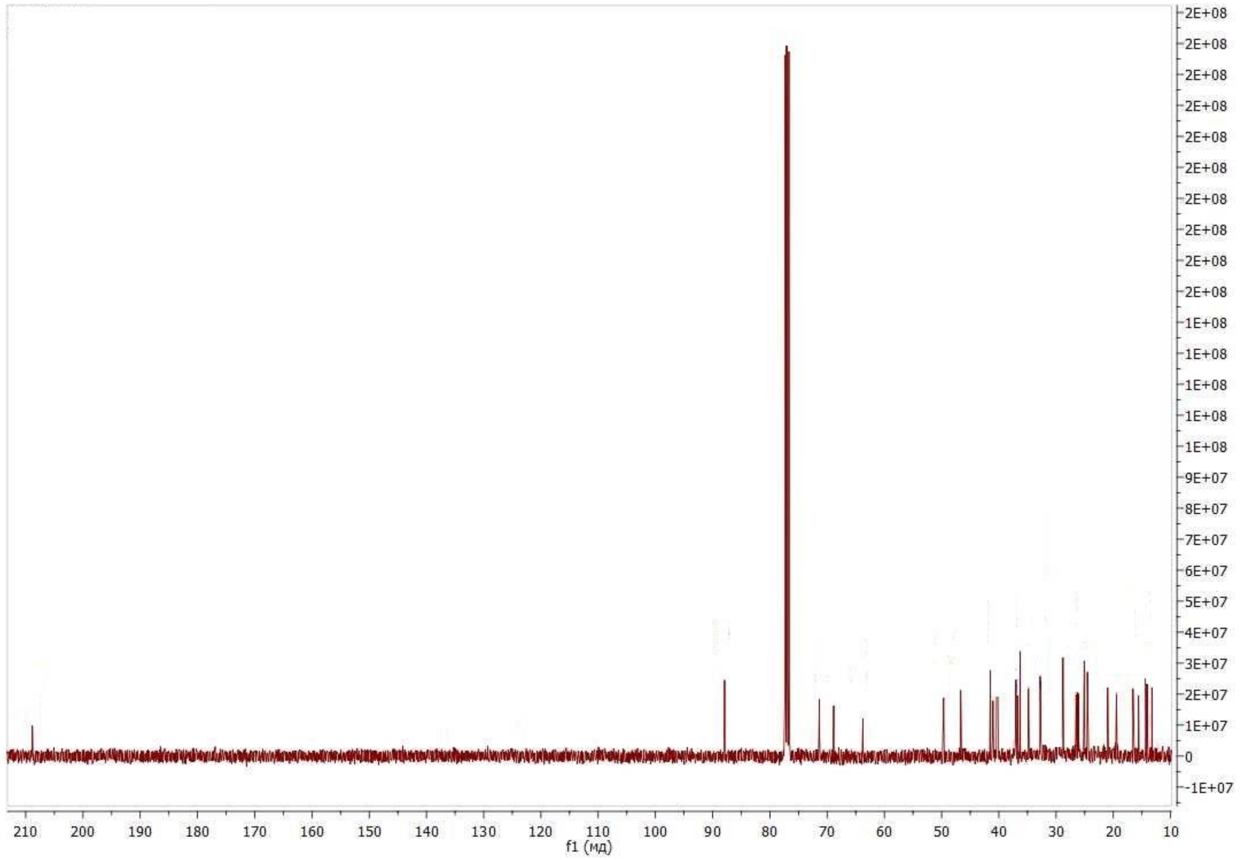

GC-MS (2*S*,3*S*)-2,3-dimethyl-(2,3),(19β,28)-diepoxy-18α*H*-olean-1-one (16)

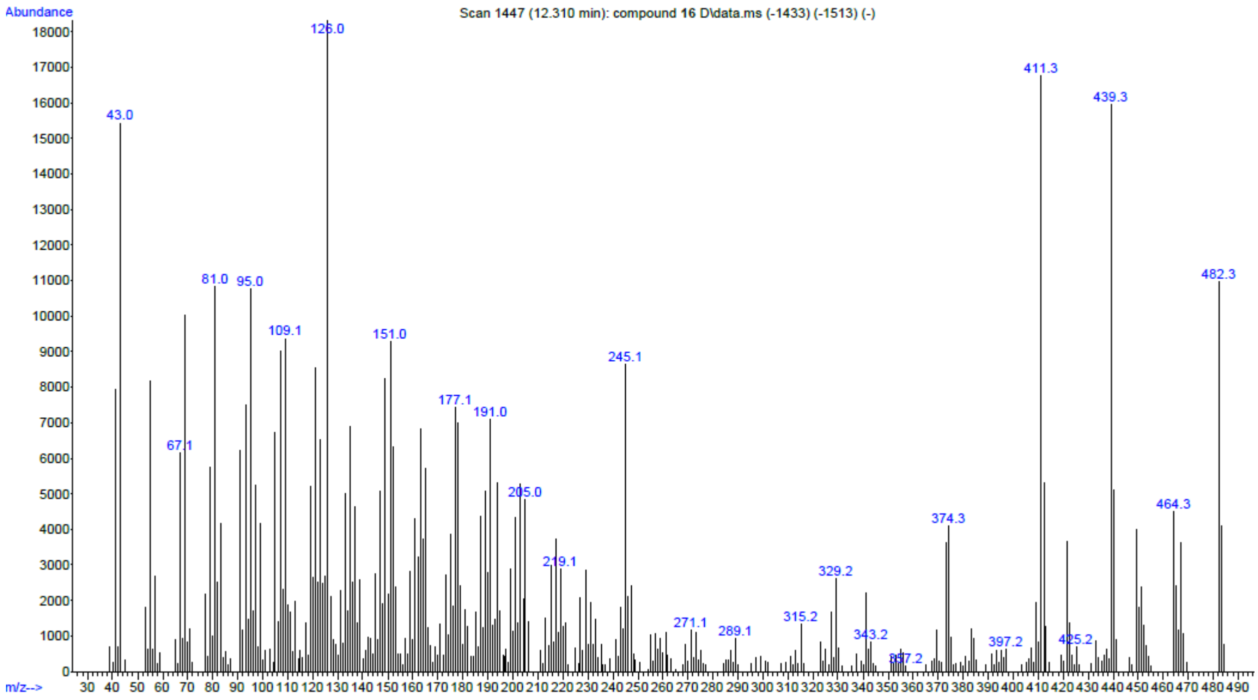

^{1}H NMR (2*S*,3*S*)-3-ethyl-2-methyl-(2,3),(19β,28)-diepoxy-18α*H*-olean-1-one (17)

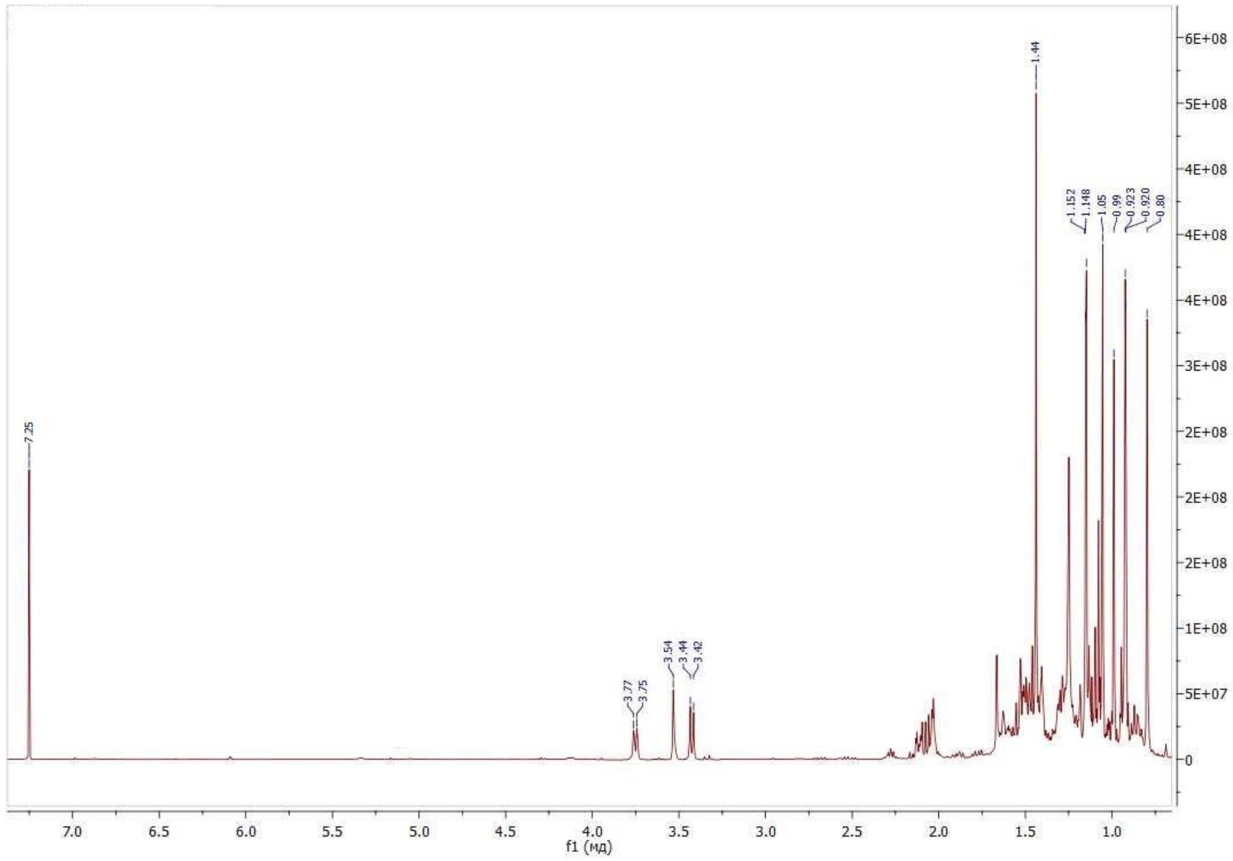

^{13}C NMR ($CDCl_3$) (2*S*,3*S*)-3-ethyl-2-methyl-(2,3),(19β,28)-diepoxy-18α*H*-olean-1-one (17)

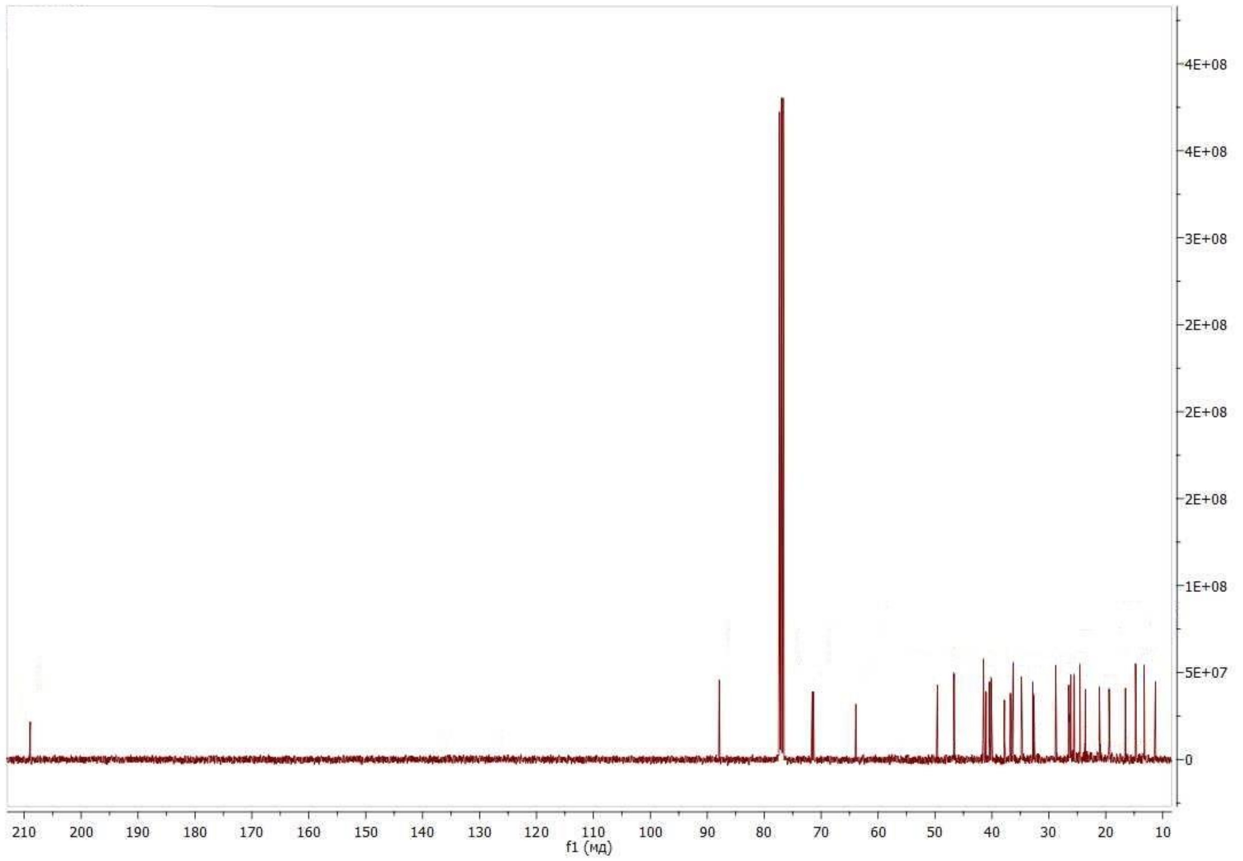

GC-MS (2*S*,3*S*)-3-ethyl-2-methyl-(2,3),(19β,28)-diepoxy-18α*H*-olean-1-one (17)

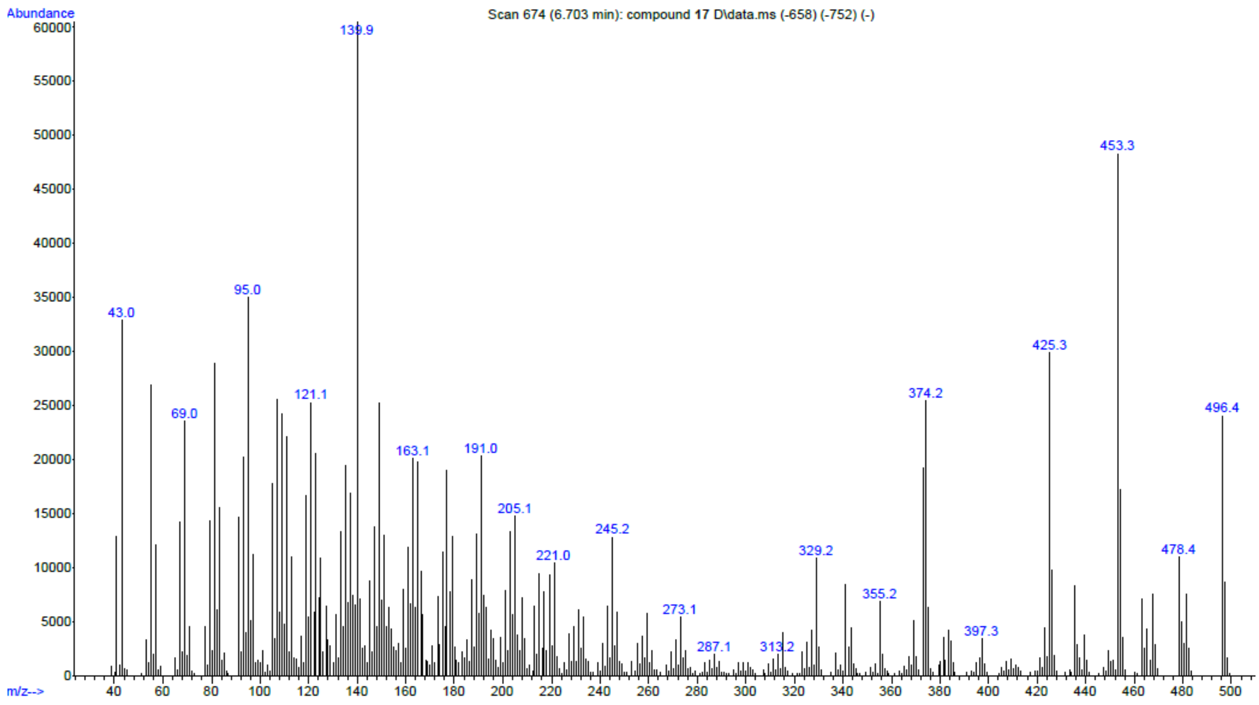

Single crystal X-ray crystallographic analysis

Table S1. Crystal data and structure refinement for **3**.

Empirical formula	$C_{31}H_{50}O_2$
Formula weight	454.71

Temperature, K	295.15
Crystal system	orthorhombic
Space group	$P2_12_12_1$
a, Å	7.8661(9)
b, Å	18.674(2)
c, Å	36.982(6)
Volume, $Å^3$	5432.2(13)
Z	8
Density (calculated), g/cm^3	1.112
Absorption coefficient, mm^{-1}	0.067
F(000)	2016.0
Crystal size, mm^3	0.5 × 0.32 × 0.15
Radiation	MoKα (λ = 0.71073)
2Θ range for data collection, °	5.924 to 58.69
Index ranges	$-10 \le h \le 7$, $-21 \le k \le 25$, $-48 \le l \le 45$
Reflections collected	35617
Independent reflections	13094 [R_{int} = 0.0458, R_{sigma} = 0.0720]
Data/restraints/parameters	13094/2/619
Goodness-of-fit on F^2	1.028
Final R indexes [I>=2σ (I)]	R_1 = 0.0615, wR_2 = 0.1164
Final R indexes [all data]	R_1 = 0.1138, wR_2 = 0.1417
Largest diff. peak/hole, $eÅ^{-3}$	0.19/-0.18

Table S2. Crystal data and structure refinement for **4a**.

Empirical formula	$C_{31}H_{48}O_3$
Formula weight	468.69
Temperature, K	295.15
Crystal system	monoclinic
Space group	$P2_1$
a, Å	6.8715(19)
b, Å	12.581(3)
c, Å	15.706(4)
β, °	99.87(2)
Volume, $Å^3$	1337.7(6)
Z	2
Density (calculated), g/cm^3	1.164
Absorption coefficient, mm^{-1}	0.072
F(000)	516.0
Crystal size, mm^3	0.48 × 0.2 × 0.12
Radiation	MoKα (λ = 0.71073)
2Θ range for data collection, °	6.018 to 58.694
Index ranges	$-9 \le h \le 8$, $-16 \le k \le 17$, $-20 \le l \le 19$
Reflections collected	11784
Independent reflections	6210 [R_{int} = 0.0344, R_{sigma} = 0.0488]
Data/restraints/parameters	6210/1/315
Goodness-of-fit on F^2	1.028

Final R indexes [I>=2σ (I)]	R_1 = 0.0545, wR_2 = 0.1188
Final R indexes [all data]	R_1 = 0.0746, wR_2 = 0.1347
Largest diff. peak/hole, eÅ^{-3}	0.14/-0.21

Table S3. Crystal data and structure refinement for **7**.

Empirical formula	$C_{32}H_{52}O_2$
Formula weight	468.73
Temperature, K	295.15
Crystal system	orthorhombic
Space group	$P2_12_12_1$
a, Å	12.059(3)
b, Å	13.264(3)
c, Å	17.403(3)
Volume, Å^3	2783.6(11)
Z	4
Density (calculated), g/cm^3	1.118
Absorption coefficient, mm^{-1}	0.067
F(000)	1040.0
Crystal size, mm^3	0.56 × 0.45 × 0.22
Radiation	MoKα (λ = 0.71073)
2Θ range for data collection, °	6.144 to 58.632
Index ranges	-15 ≤ h ≤ 11, -15 ≤ k ≤ 17, -15 ≤ l ≤ 23
Reflections collected	10656
Independent reflections	6080 [R_{int} = 0.0272, R_{sigma} = 0.0513]
Data/restraints/parameters	6080/0/320
Goodness-of-fit on F^2	1.039
Final R indexes [I>=2σ (I)]	R_1 = 0.0551, wR_2 = 0.1143
Final R indexes [all data]	R_1 = 0.0801, wR_2 = 0.1300
Largest diff. peak/hole, eÅ^{-3}	0.20/-0.16

Table S4. Crystal data and structure refinement for **8**.

Empirical formula	$C_{33}H_{54}O_2$
Formula weight	482.76
Temperature, K	295.15
Crystal system	orthorhombic
Space group	$P2_12_12_1$
a, Å	12.230(3)
b, Å	13.313(3)
c, Å	17.488(3)
Volume, Å^3	2847.5(11)
Z	4
Density (calculated), g/cm^3	1.126
Absorption coefficient, mm^{-1}	0.067
F(000)	1072.0
Crystal size, mm^3	0.5 × 0.35 × 0.22

Radiation	MoKα (λ = 0.71073)
2Θ range for data collection, °	6.494 to 58.89
Index ranges	-14 ≤ h ≤ 15, -18 ≤ k ≤ 14, -23 ≤ l ≤ 22
Reflections collected	14727
Independent reflections	6740 [R_{int} = 0.0347, R_{sigma} = 0.0544]
Data/restraints/parameters	6740/1/329
Goodness-of-fit on F^2	1.022
Final R indexes [I>=2σ (I)]	R_1 = 0.0623, wR_2 = 0.1413
Final R indexes [all data]	R_1 = 0.0884, wR_2 = 0.1603
Largest diff. peak/hole, eÅ^{-3}	0.40/-0.22

Table S5. Crystal data and structure refinement for **12**.

Empirical formula	$C_{37}H_{54}O_3$
Formula weight	546.80
Temperature, K	295.15
Crystal system	monoclinic
Space group	$P2_1$
a, Å	7.7161(12)
b, Å	20.087(3)
c, Å	23.884(4)
β, °	94.519(15)
Volume, Å^3	3690.5(10)
Z	4
Density (calculated), g/cm^3	0.984
Absorption coefficient, mm^{-1}	0.060
F(000)	1200.0
Crystal size, mm^3	0.46 × 0.28 × 0.12
Radiation	MoKα (λ = 0.71073)
2Θ range for data collection, °	5.518 to 58.798
Index ranges	-9 ≤ h ≤ 10, -25 ≤ k ≤ 27, -30 ≤ l ≤ 32
Reflections collected	42332
Independent reflections	17443 [R_{int} = 0.0596, R_{sigma} = 0.0944]
Data/restraints/parameters	17443/3/745
Goodness-of-fit on F^2	0.933
Final R indexes [I>=2σ (I)]	R_1 = 0.0599, wR_2 = 0.1410
Final R indexes [all data]	R_1 = 0.1365, wR_2 = 0.1820
Largest diff. peak/hole, eÅ^{-3}	0.15/-0.15

Table S6. Crystal data and structure refinement for **13**.

Empirical formula	$C_{31}H_{50}O_3$
Formula weight	470.71
Temperature, K	295.15
Crystal system	monoclinic
Space group	$P2_1$
a, Å	7.102(2)

b, Å	12.082(5)
c, Å	15.784(7)
β, °	95.88(4)
Volume, $Å^3$	1347.2(9)
Z	2
Density (calculated), g/cm^3	1.160
Absorption coefficient, mm^{-1}	0.072
F(000)	520.0
Crystal size, mm^3	0.5 × 0.4 × 0.02
Radiation	Mo Kα (λ = 0.71073)
2Θ range for data collection, °	6.076 to 58.776
Index ranges	$-9 \le h \le 7$, $-16 \le k \le 10$, $-20 \le l \le 17$
Reflections collected	6513
Independent reflections	4585 [R_{int} = 0.0587, R_{sigma} = 0.1026]
Data/restraints/parameters	4585/1/319
Goodness-of-fit on F^2	1.025
Final R indexes [I>=2σ (I)]	R_1 = 0.0761, wR_2 = 0.1794
Final R indexes [all data]	R_1 = 0.1293, wR_2 = 0.2248
Largest diff. peak/hole, $eÅ^{-3}$	0.25/-0.26

Table S7. Crystal data and structure refinement for **14**.

Empirical formula	$C_{31}H_{50}O_3 \cdot 2CHCl_3$
Formula weight	709.44
Temperature, K	295.15
Crystal system	orthorhombic
Space group	$P2_12_12_1$
a, Å	10.753(2)
b, Å	11.3197(17)
c, Å	29.710(7)
Volume, $Å^3$	3616.2(12)
Z	4
Density (calculated), g/cm^3	1.303
Absorption coefficient, mm^{-1}	0.506
F(000)	1504.0
Crystal size, mm^3	0.45 × 0.35 × 0.2
Radiation	Mo Kα (λ = 0.71073)
2Θ range for data collection, °	5.902 to 58.562
Index ranges	$-13 \le h \le 14$, $-15 \le k \le 9$, $-25 \le l \le 40$
Reflections collected	17455
Independent reflections	8303 [R_{int} = 0.0429, R_{sigma} = 0.0778]
Data/restraints/parameters	8303/0/388
Goodness-of-fit on F^2	1.023
Final R indexes [I>=2σ (I)]	R_1 = 0.0679, wR_2 = 0.1544
Final R indexes [all data]	R_1 = 0.1453, wR_2 = 0.1940
Largest diff. peak/hole, $eÅ^{-3}$	0.28/-0.28
Flack parameter	0.02(4)

Table S8. Crystal data and structure refinement for **15**.

Empirical formula	$C_{33}H_{54}O_3$
Formula weight	498.76
Temperature, K	295.15
Crystal system	orthorhombic
Space group	$P2_12_12_1$
a, Å	7.2239(17)
b, Å	12.282(3)
c, Å	32.677(7)
Volume, $Å^3$	2899.1(12)
Z	4
Density (calculated), g/cm^3	1.143
Absorption coefficient, mm^{-1}	0.070
F(000)	1104.0
Crystal size, mm^3	0.5 × 0.15 × 0.1
Radiation	Mo Kα (λ = 0.71073)
2Θ range for data collection, °	5.776 to 59.188
Index ranges	$-6 \leq h \leq 9$, $-16 \leq k \leq 10$, $-30 \leq l \leq 43$
Reflections collected	11280
Independent reflections	6564 [R_{int} = 0.0374, R_{sigma} = 0.0640]
Data/restraints/parameters	6564/0/338
Goodness-of-fit on F^2	1.028
Final R indexes [I>=2σ (I)]	R_1 = 0.0701, wR_2 = 0.1667
Final R indexes [all data]	R_1 = 0.1005, wR_2 = 0.1948
Largest diff. peak/hole, $eÅ^{-3}$	0.44/-0.23

Table S9. Crystal data and structure refinement for **16**.

Empirical formula	$C_{32}H_{50}O_3$
Formula weight	482.72
Temperature, K	295.15
Crystal system	monoclinic
Space group	$P2_1$
a, Å	6.7002(16)
b, Å	15.587(3)
c, Å	13.665(4)
β, °	103.50(3)
Volume, $Å^3$	1387.7(6)
Z	2
Density (calculated), g/cm^3	1.155
Absorption coefficient, mm^{-1}	0.072
F(000)	532.0
Crystal size, mm^3	0.55 × 0.36 × 0.24
Radiation	MoKα (λ = 0.71073)
2Θ range for data collection, °	6.06 to 58.75
Index ranges	$-8 \leq h \leq 9$, $-20 \leq k \leq 20$, $-18 \leq l \leq 11$

Reflections collected	7208
Independent reflections	5266 [R_{int} = 0.0391, R_{sigma} = 0.0549]
Data/restraints/parameters	5266/1/325
Goodness-of-fit on F^2	1.032
Final R indexes [I>=2σ (I)]	R_1 = 0.0585, wR_2 = 0.1389
Final R indexes [all data]	R_1 = 0.0788, wR_2 = 0.1595
Largest diff. peak/hole, eÅ^{-3}	0.23/-0.22

Table S10. Crystal data and structure refinement for **17**.

Empirical formula	$C_{33}H_{52}O_3$
Formula weight	496.74
Temperature, K	295.15
Crystal system	monoclinic
Space group	$P2_1$
a, Å	8.011(3)
b, Å	15.691(4)
c, Å	11.978(4)
β, °	107.98(4)
Volume, Å^3	1432.1(8)
Z	2
Density (calculated), g/cm^3	1.152
Absorption coefficient, mm^{-1}	0.071
F(000)	548.0
Crystal size, mm^3	0.56 × 0.44 × 0.35
Radiation	MoKα (λ = 0.71073)
2Θ range for data collection, °	5.944 to 58.522
Index ranges	$-10 \le h \le 10$, $-20 \le k \le 21$, $-15 \le l \le 14$
Reflections collected	7225
Independent reflections	5551 [R_{int} = 0.0463, R_{sigma} = 0.0757]
Data/restraints/parameters	5551/1/334
Goodness-of-fit on F^2	1.022
Final R indexes [I>=2σ (I)]	R_1 = 0.0724, wR_2 = 0.1681
Final R indexes [all data]	R_1 = 0.1003, wR_2 = 0.2028
Largest diff. peak/hole, eÅ^{-3}	0.28/-0.26
